# Supplementary material for: Multiplex gene quantification as digital markers for extremely rapid evaluation of chemo-drug sensitivity
Source: Patterns (N Y). 2021 Sep 29;2(10):100360. doi: 10.1016/j.patter.2021.100360 (PMC8515010; doi:10.1016/j.patter.2021.100360)
Supplement: Document S1. Figures S1–S16 and Tables S1–S7 [file mmc1.pdf]

Patterns, Volume *2*

Supplemental information

Multiplex gene quantification as digital markers for extremely rapid evaluation of chemo-drug sensitivity

Jiaqi Fan, Yilin Feng, Yifan Cheng, Zitian Wang, Haoran Zhao, Edgar A. Galan, Quanxing Liao, Shuzhong Cui, Weijie Zhang, and Shaohua Ma

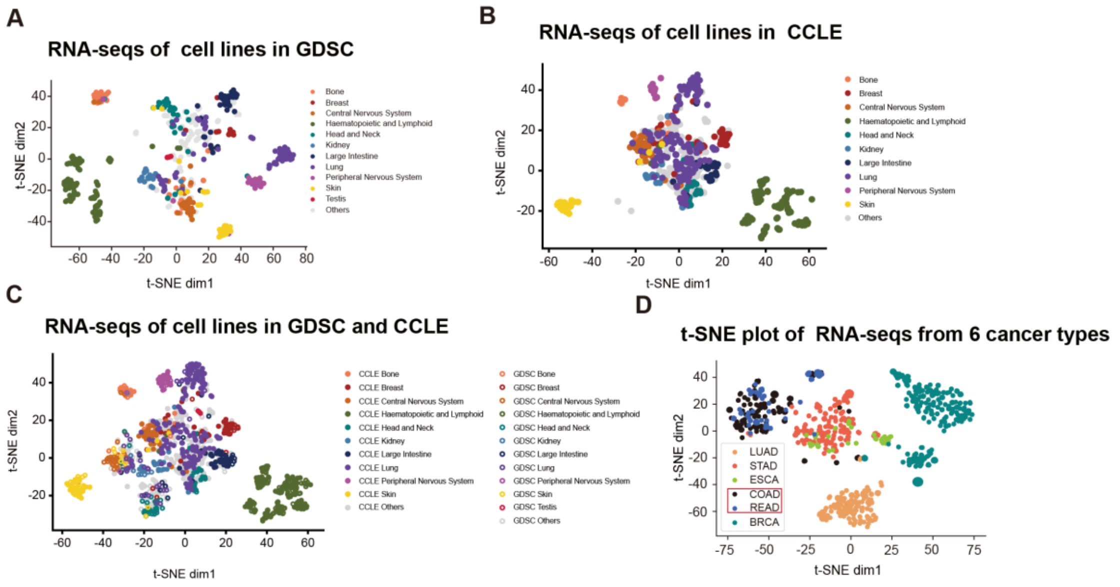

Figure S1. Two-dimensional t-SNE plot of the RNA-seq data of cancer cell lines in CCLE and GDSC, and patient samples in TCGA. (A, B) Two-dimensional t-SNE plot of the RNA-seq data of cancer cell lines in GDSC (A) and CCLE (B). Colors denote cancer types. Each dot represents a cell line. (C) Two-dimensional t-SNE plot of the RNA-seq data of cancer cell lines in CCLE and GDSC. Colors denote cancer types. Each dot represents a cell line. The filled circles denote cell lines from CCLE, and the hollow circles denote cell lines from GDSC. (D) Two-dimensional t-SNE plot of the RNA-seq data of 6 types of tumors. Each color represents a tumor type. Each dot represents a tumor sample.

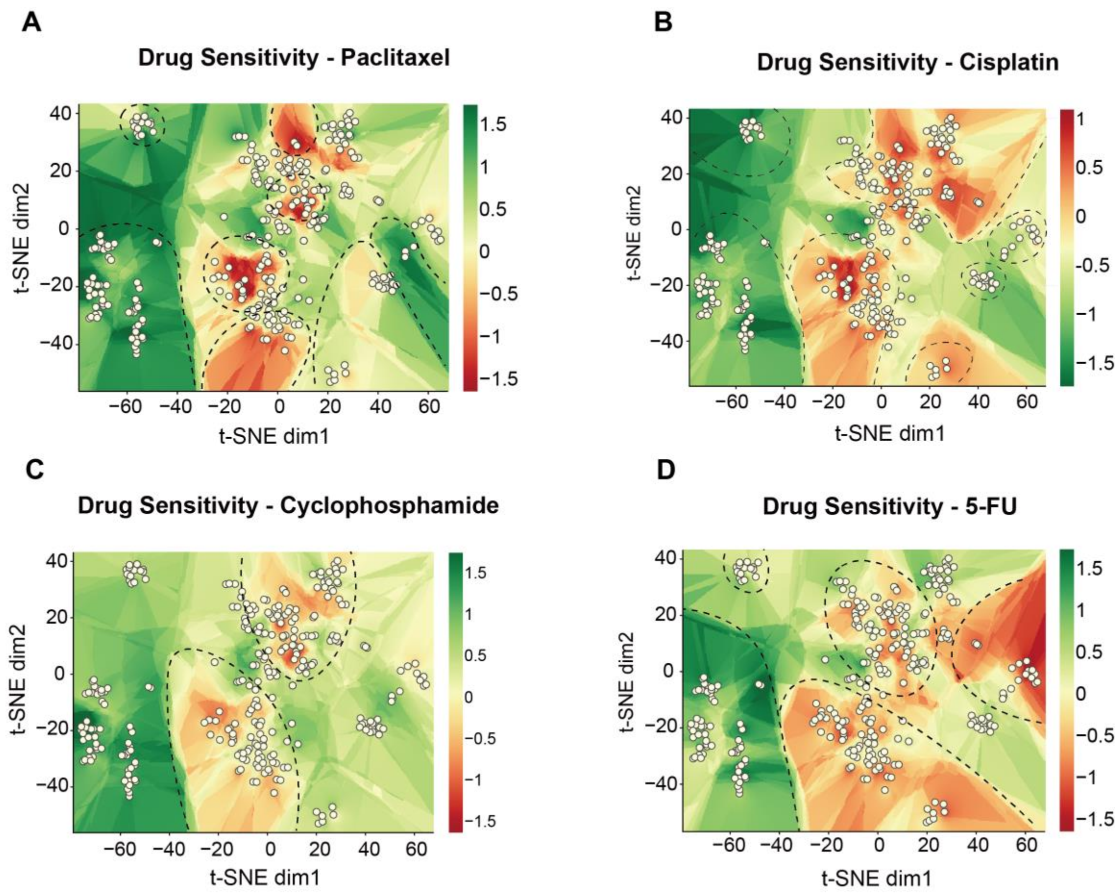

Figure S2. Cancer type-dependent drug sensitivity distribution. Drug sensitivity distribution toward Paclitaxel(A), Cisplatin(B), Cyclophosphamide(C) and 5-FU(D). The dots represent cancer cell lines mapped by t-SNE as in **Supplementary Fig. 1A**. Colors indicate the sensitivity magnitude (z-score of IC50; green, sensitive; red, non-sensitive) predicted by KNN regression in the two-dimensional area. For KNN regression, k=7.

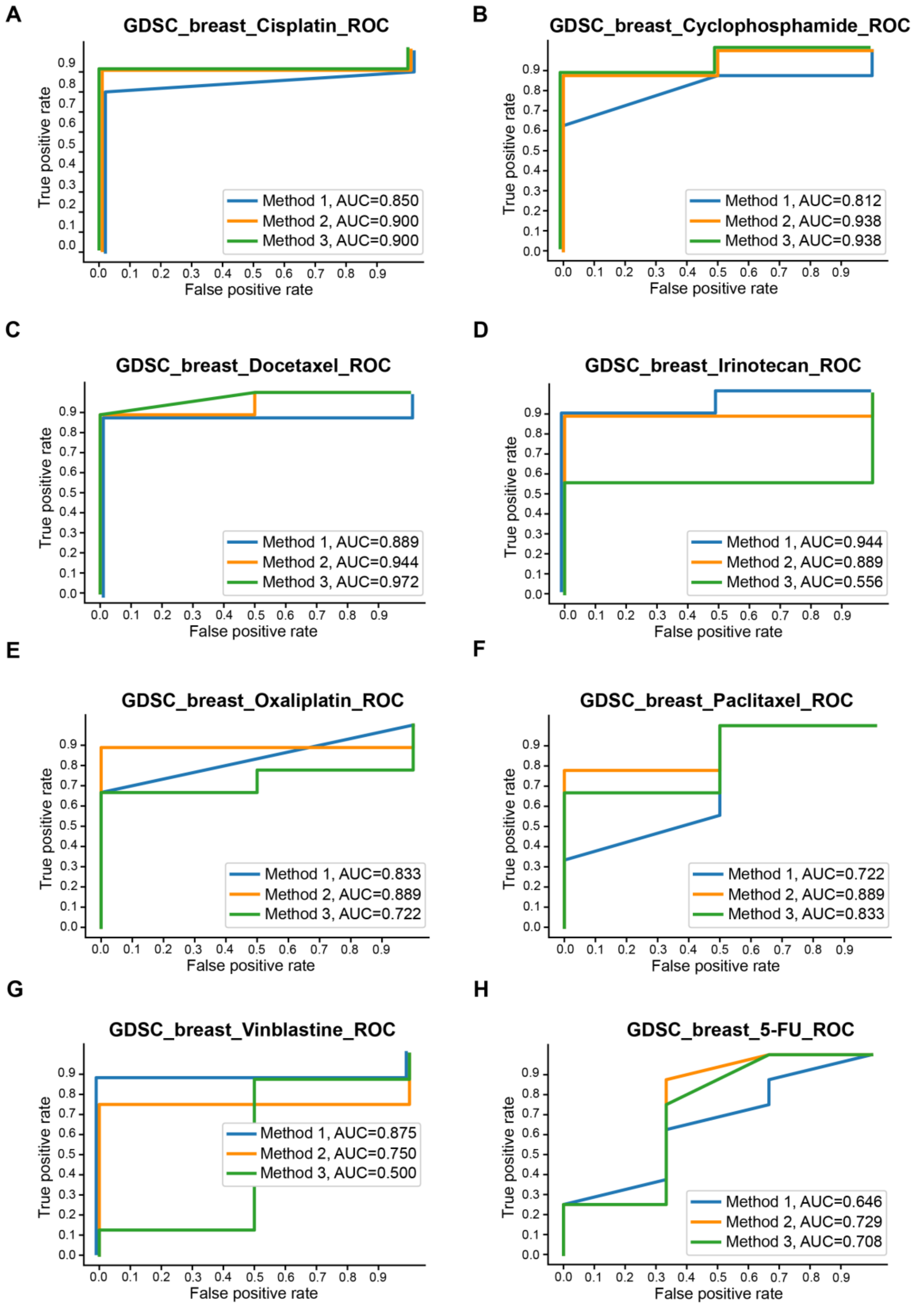

Figure S3. ROC curves for cross-validation of drug sensitivity prediction on breast cancer cell lines in GDSC. The three curves in each plot refer to filtering and scoring genes on both datasets (Method 1), on single but one same dataset (Method 2), and on single but a different dataset (Method 3). IC50 z-score threshold = -1.0. Results for breast cancer and Cisplatin (A), Cyclophosphamide (B), Docetaxel (C), Irinotecan (D), Oxaliplatin (E), Paclitaxel (F), Vinblastine (G) and 5-FU (H) are shown.

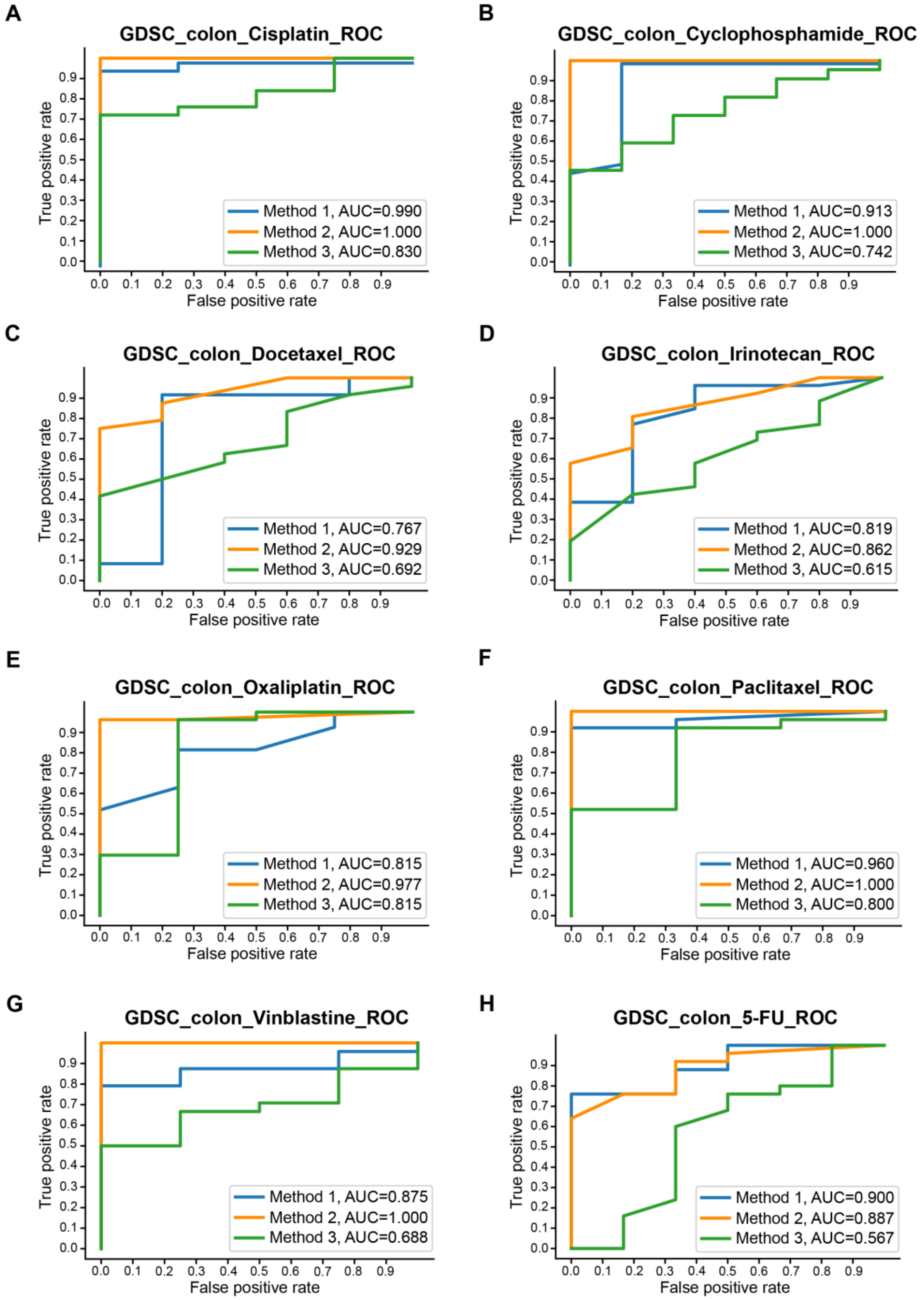

Figure S4. ROC curves for cross-validation of drug sensitivity prediction on colorectal cancer cell lines in GDSC. The three curves in each plot refer to filtering and scoring genes on both datasets (Method 1), on single but one same dataset (Method 2), and on single but a different dataset (Method 3). IC50 z-score threshold = -1.0. Results for colorectal cancer and Cisplatin (A), Cyclophosphamide (B), Docetaxel (C), Irinotecan (D), Oxaliplatin (E), Paclitaxel (F), Vinblastine (G) and 5-FU (H) are shown.

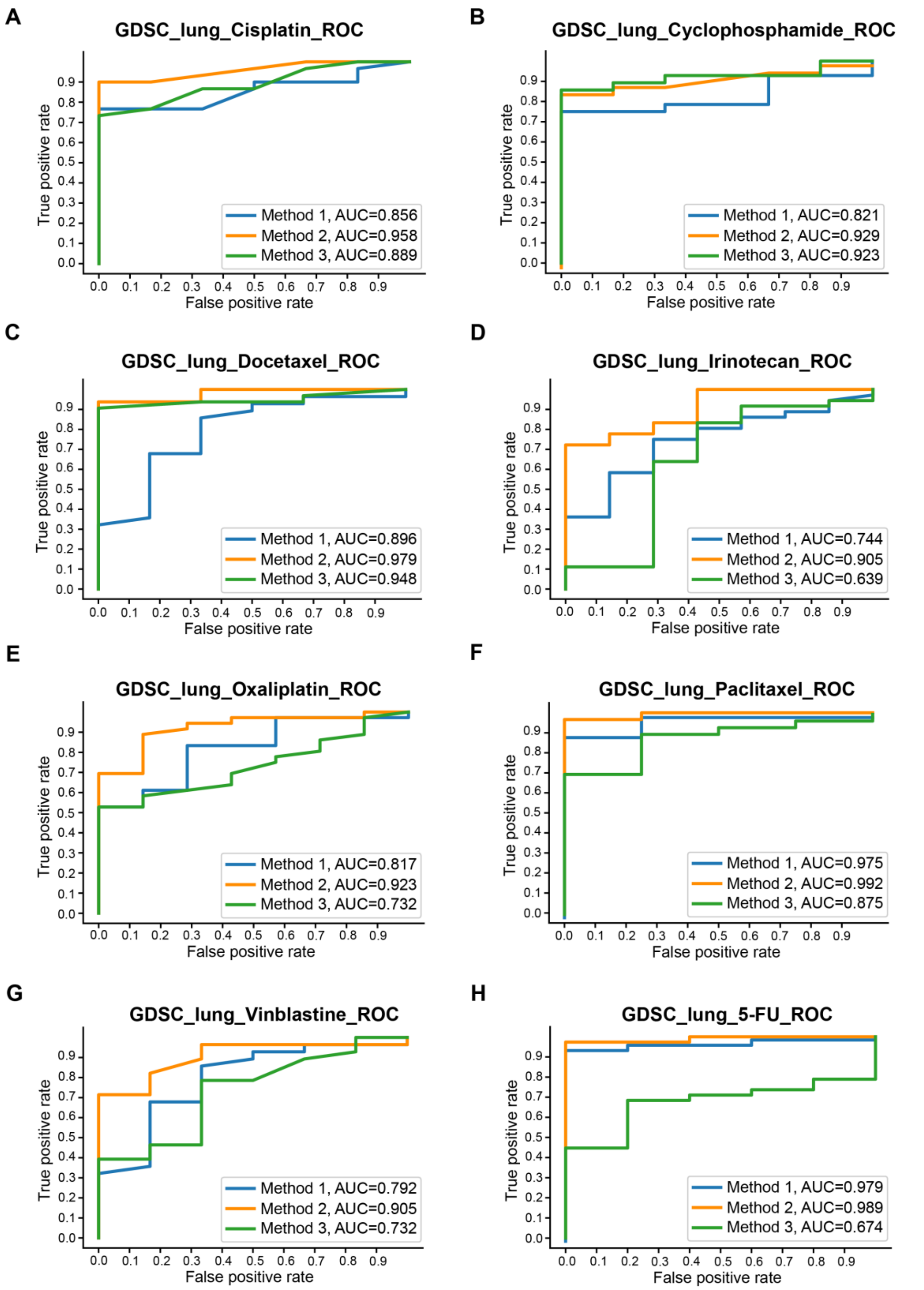

Figure S5. ROC curves for cross-validation of drug sensitivity prediction on lung cancer cell lines in GDSC. The three curves in each plot refer to filtering and scoring genes on both datasets (Method 1), on single but one same dataset (Method 2), and on single but a different dataset (Method 3). IC50 z-score threshold = -1.0. Results for lung cancer and Cisplatin (A), Cyclophosphamide (B), Docetaxel (C), Irinotecan (D), Oxaliplatin (E), Paclitaxel (F), Vinblastine (G) and 5-FU (H) are shown.

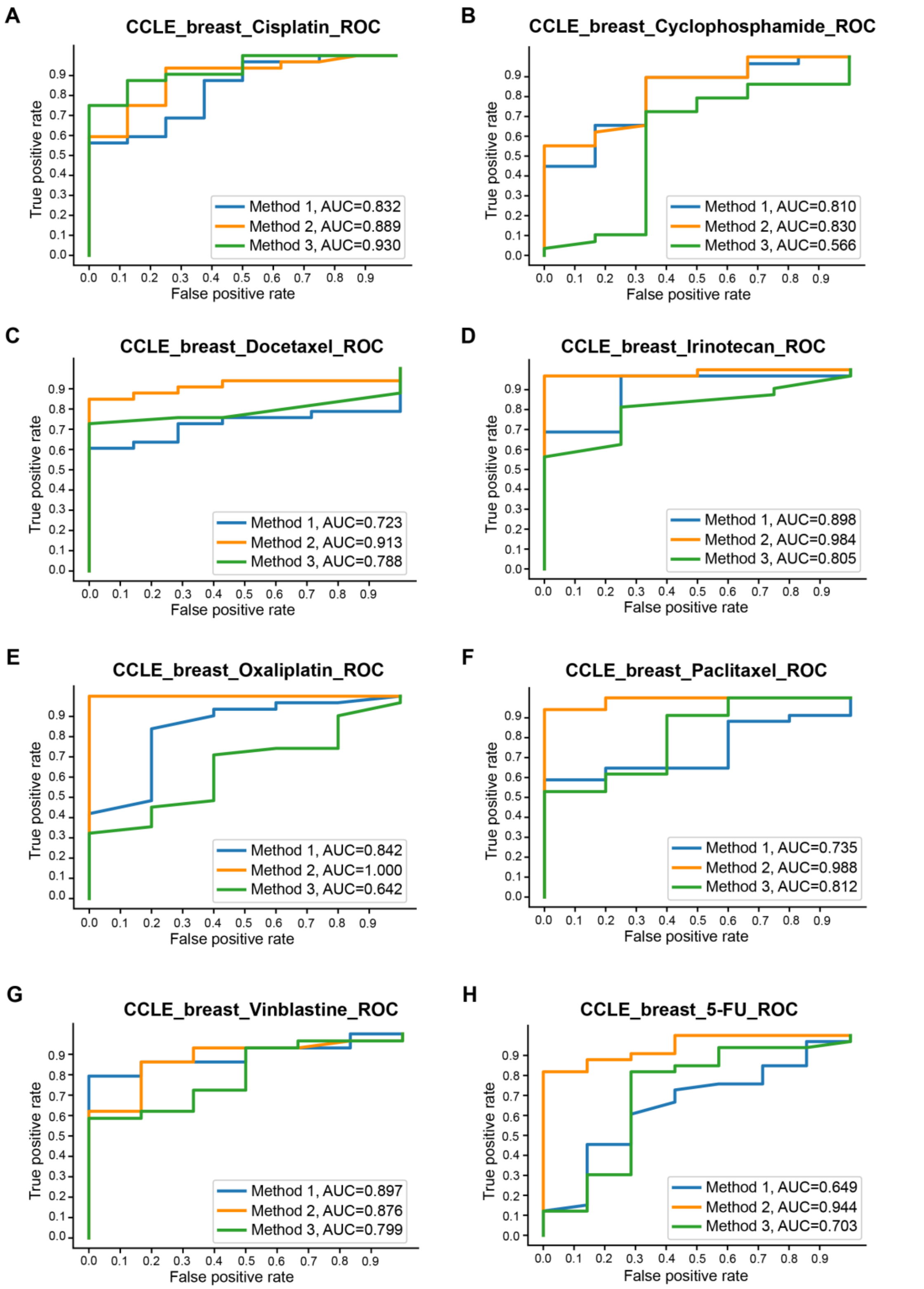

Figure S6. ROC curves for cross-validation of drug sensitivity prediction on breast cancer cell lines in CCLE. The three curves in each plot refer to filtering and scoring genes on both datasets (Method 1), on single but one same dataset (Method 2), and on single but a different dataset (Method 3). IC50 z-score threshold = -1.0. Results for breast cancer and Cisplatin (A), Cyclophosphamide (B), Docetaxel (C), Irinotecan (D), Oxaliplatin (E), Paclitaxel (F), Vinblastine (G) and 5-FU (H) are shown.

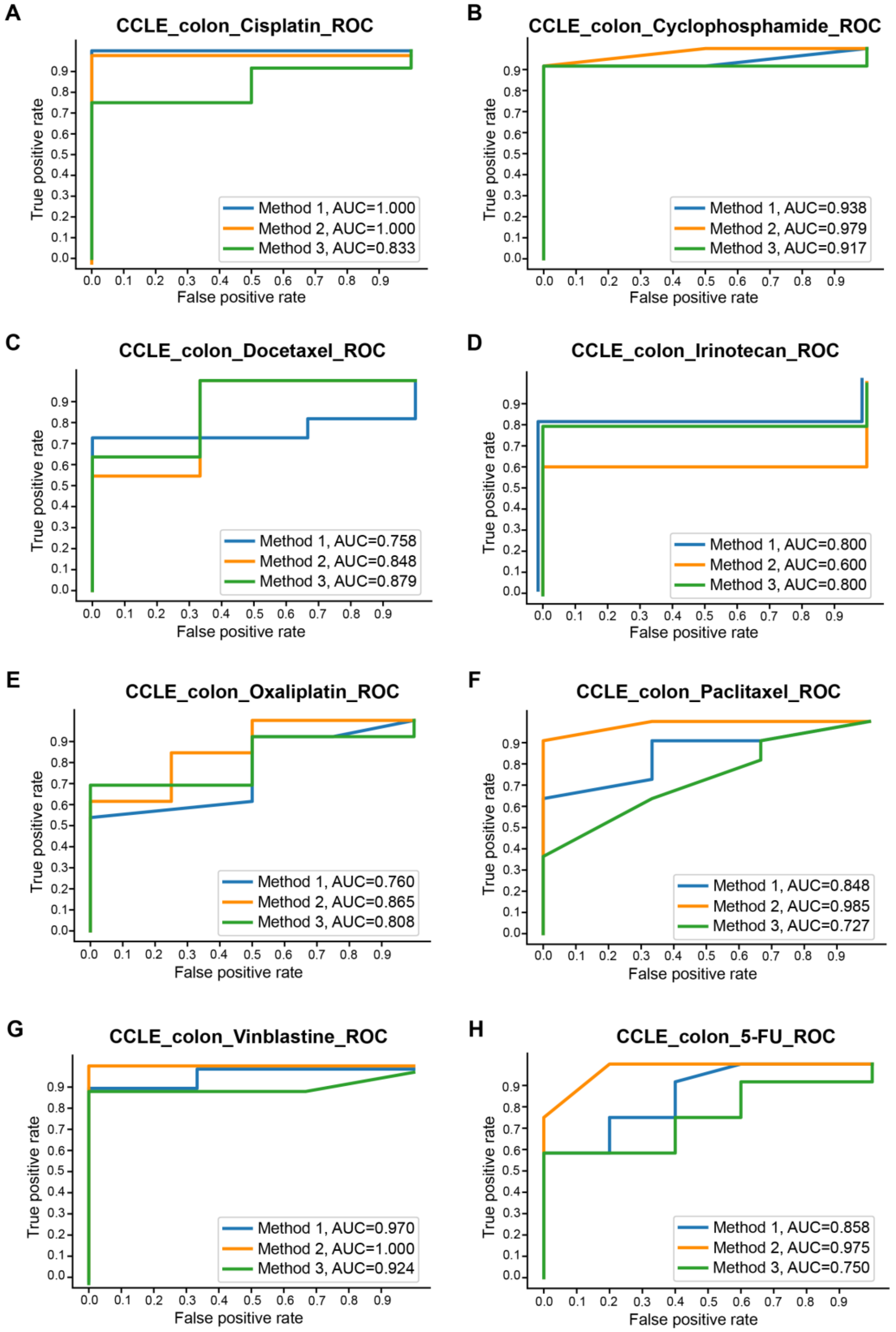

Figure S7. ROC curves for cross-validation of drug sensitivity prediction on colorectal cancer cell lines in CCLE. The three curves in each plot refer to filtering and scoring genes on both datasets (Method 1), on single but one same dataset (Method 2), and on single but a different dataset (Method 3). IC50 z-score threshold = -1.0. Results for colorectal cancer and Cisplatin (A), Cyclophosphamide (B), Docetaxel (C), Irinotecan (D), Oxaliplatin (E), Paclitaxel (F), Vinblastine (G) and 5-FU (H) are shown.

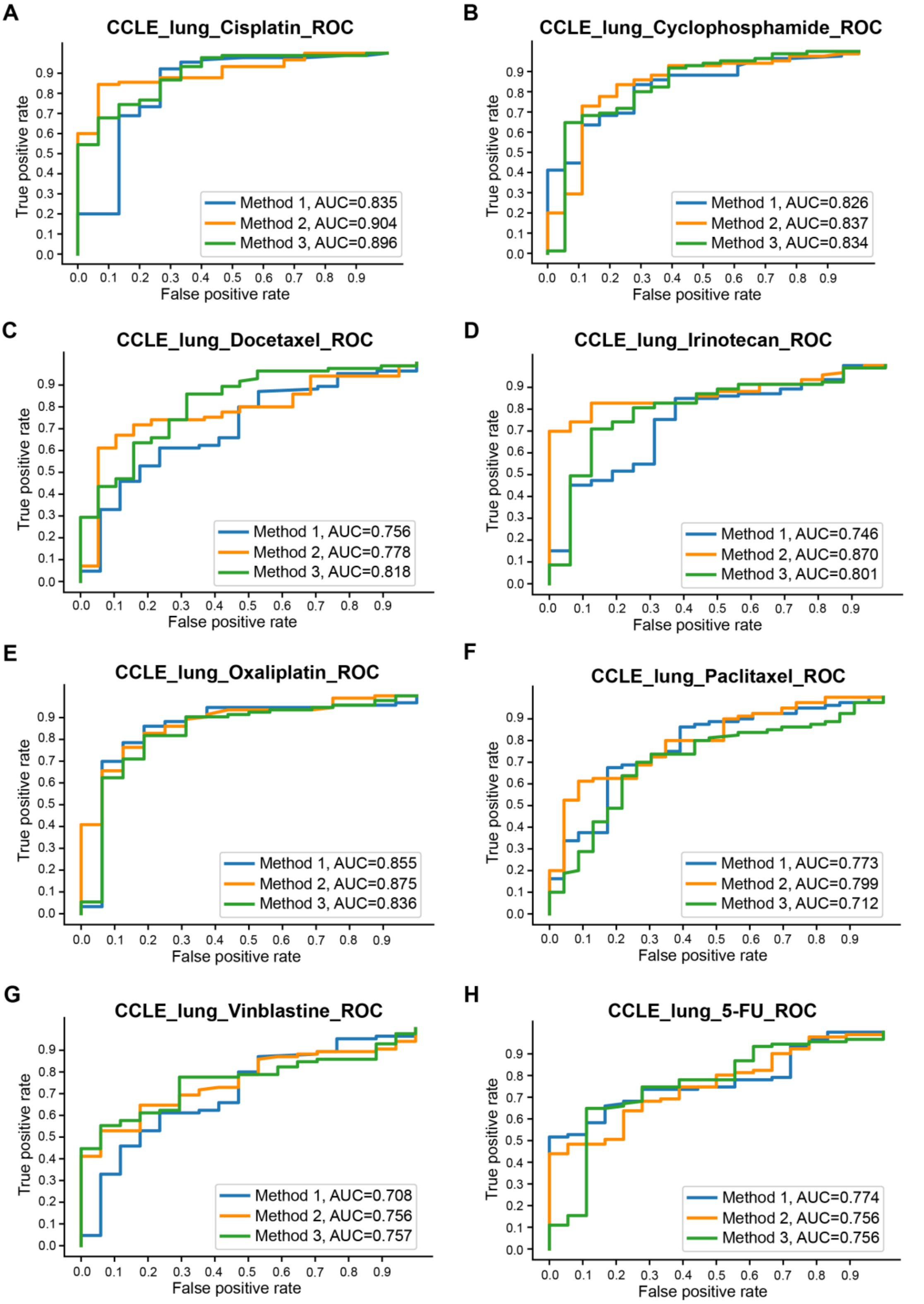

Figure S8. ROC curves for cross-validation of drug sensitivity prediction on lung cancer cell lines in CCLE. The three curves in each plot refer to filtering and scoring genes on both datasets (Method 1), on single but one same dataset (Method 2), and on single but a different dataset (Method 3). IC50 z-score threshold = -1.0. Results for lung cancer and Cisplatin (A), Cyclophosphamide (B), Docetaxel (C), Irinotecan (D), Oxaliplatin (E), Paclitaxel (F), Vinblastine (G) and 5-FU (H) are shown.

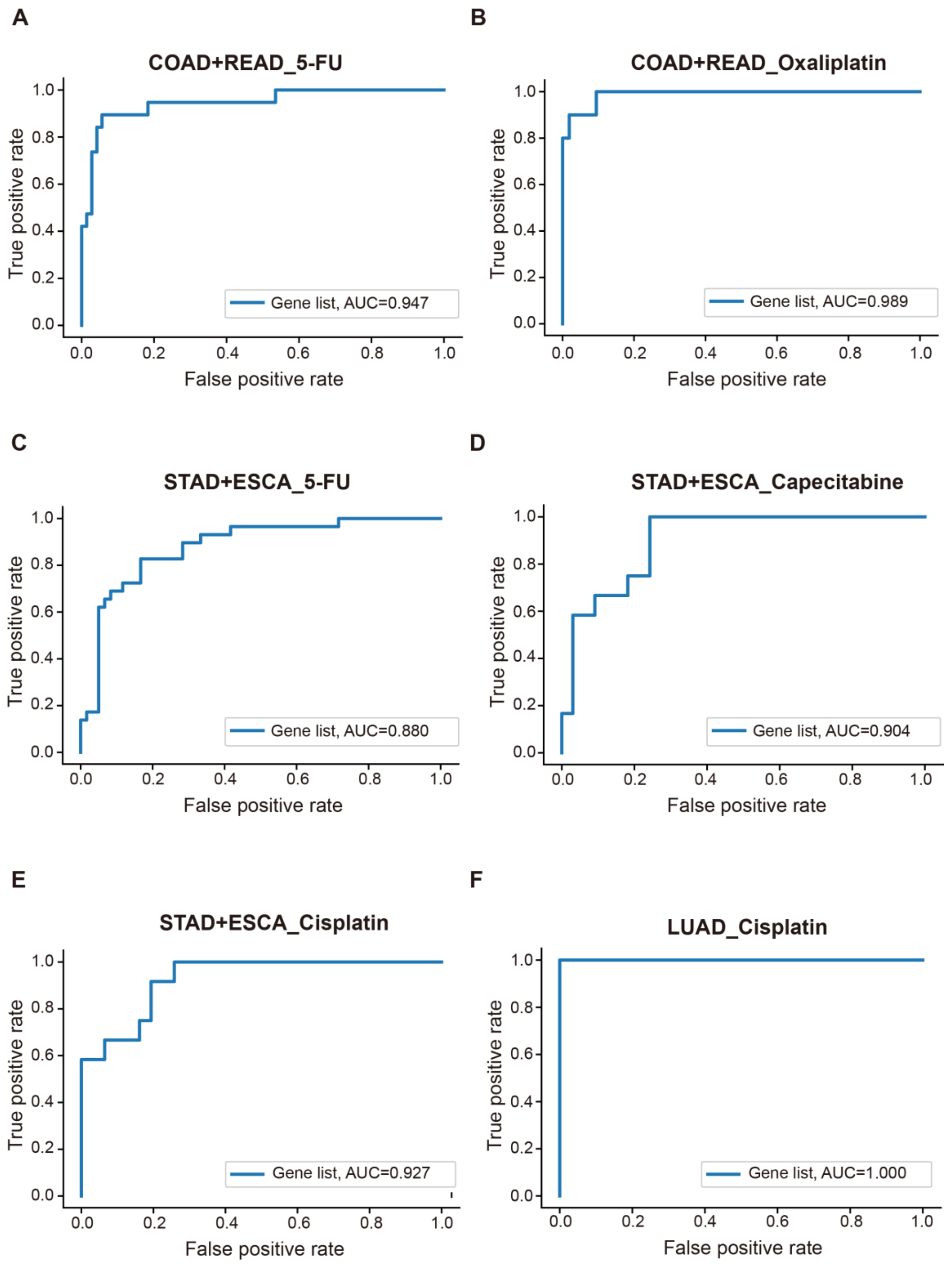

Figure S9. ROC curves for cross-validation of drug sensitivity prediction on 6 cancer type-drug pairs in TCGA. (A) COAD, READ and 5-FU. (B) COAD, READ and Oxaliplatin. (C) STAD, ESCA and 5-FU. (D) STAD, ESCA and Capecitabine. (E) STAD, ESCA and Cisplatin. (F) LUAD and Cisplatin.

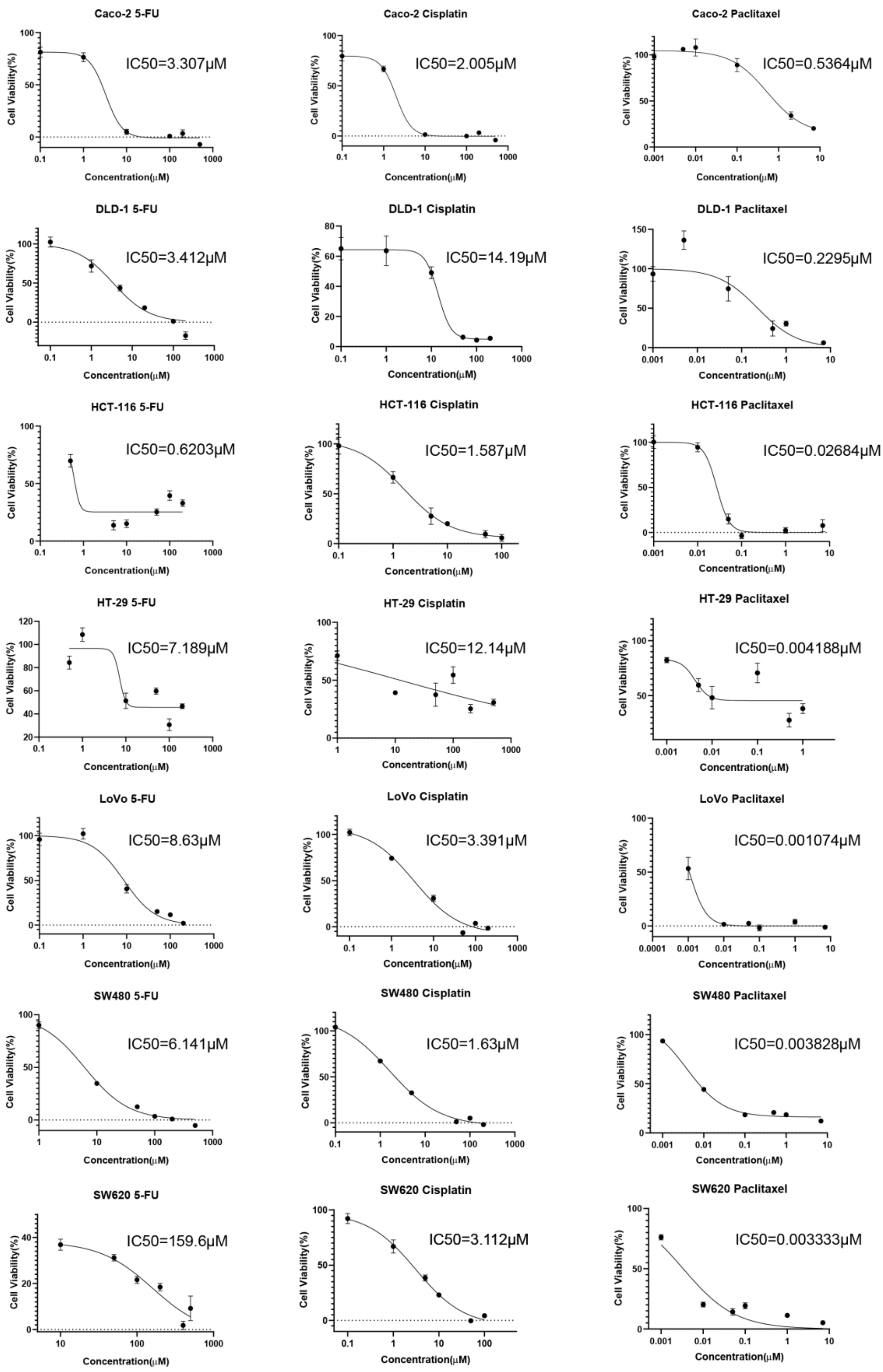

Figure S10. Drug response curves of the cancer cell lines towards 5-FU, Cisplatin and Paclitaxel. The drug response curves and IC50 values of the experimentally tested 7 colorectal cancer cell lines are shown, towards 5-FU, Cisplatin and Paclitaxel.

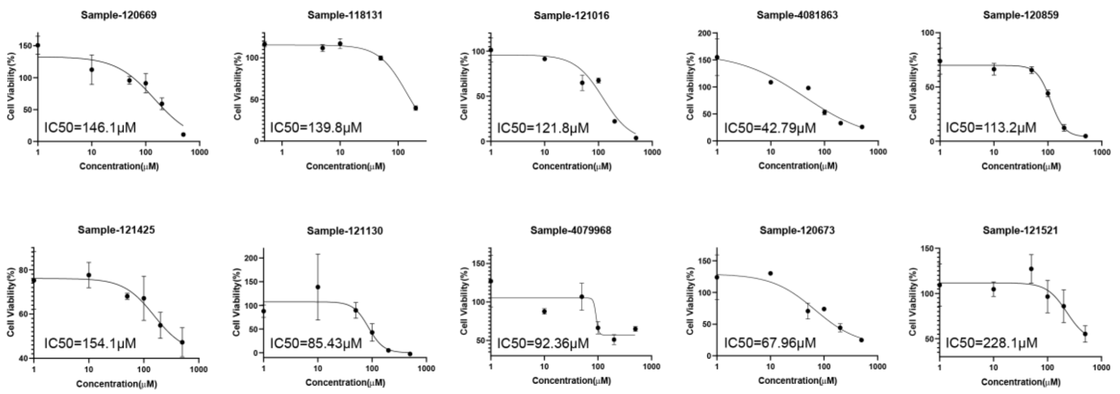

Figure S11. Drug response curves of patient tumor derived organoids towards 5-FU. The drug response curves and IC50 values towards 5-FU of the experimentally tested 10 patient tumor derived organoids, representing responses of their parental tumors, are shown.

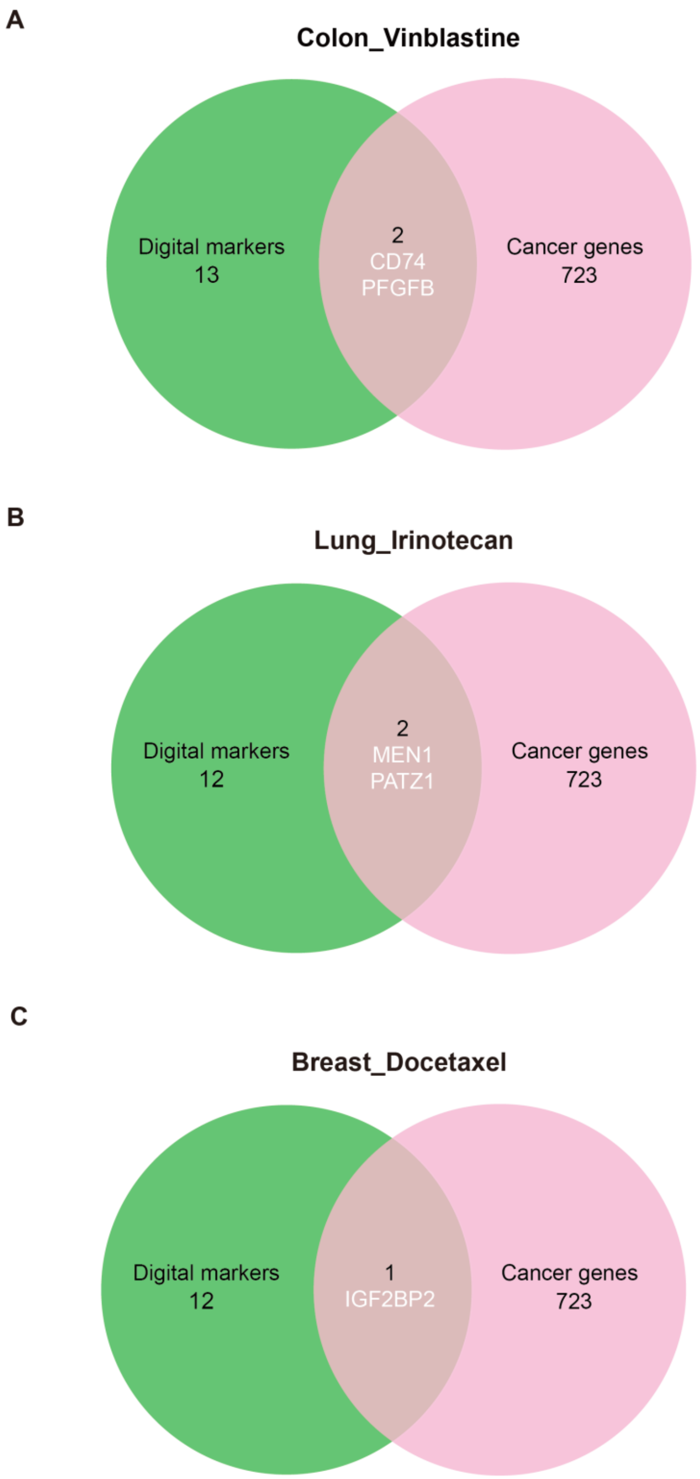

Figure S12. The Venn diagrams showing overlapping of the digital markers of cell lines and cancer genes. The overlapping of digital markers of several cancer type-drug pairs, including colon cancer and Vinblastine(A), lung cancer and Irinotecan(B), and breast cancer and Irinotecan(C) and cancer genes are shown.

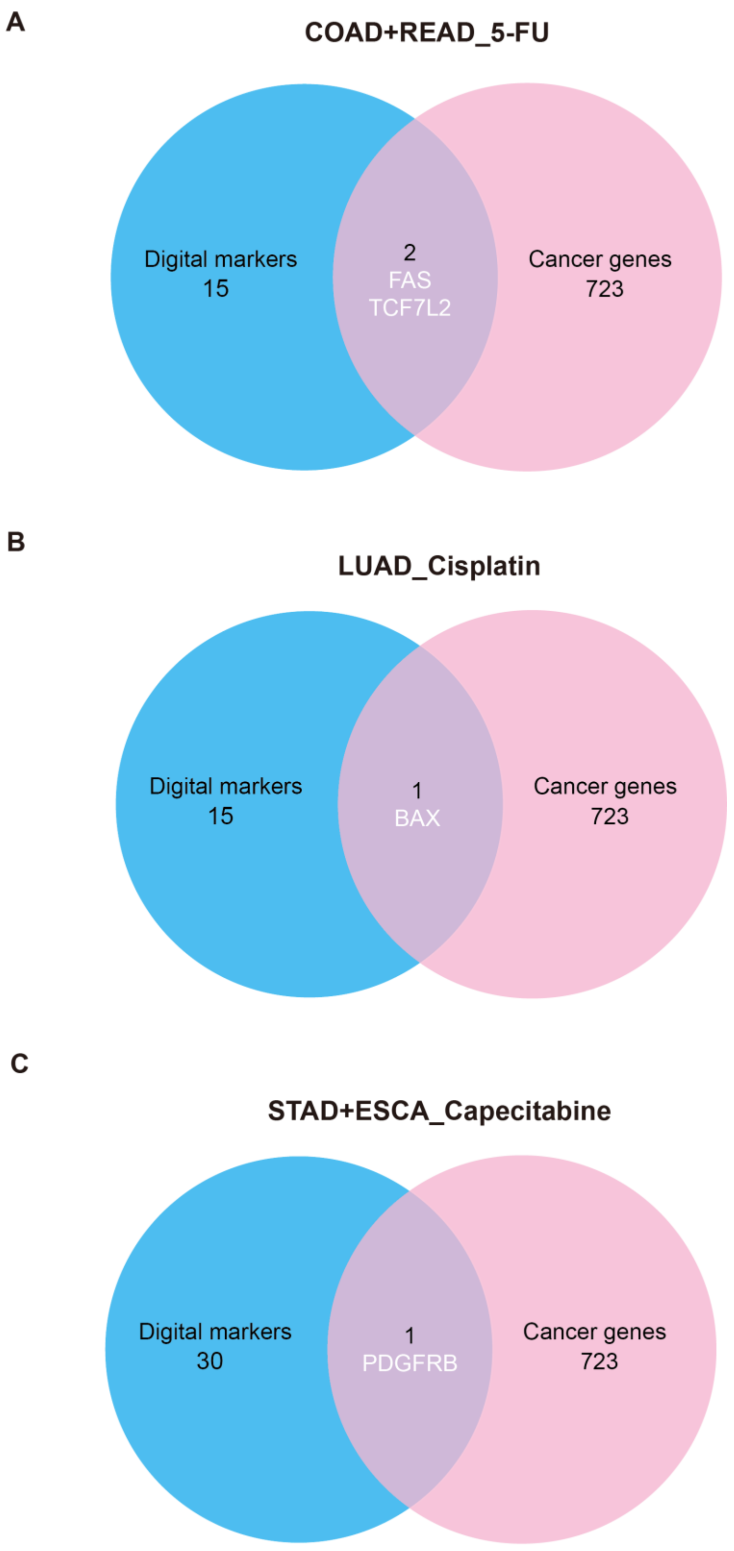

Figure S13. The Venn diagrams showing overlapping of the digital markers of patient samples and cancer genes. The overlapping of digital markers of several cancer type-drug pairs, including COAD, READ and 5-FU(A), LUAD and Ciaplatin (B), and STAD, ESCA and Capecitabine(C) and cancer genes are shown.

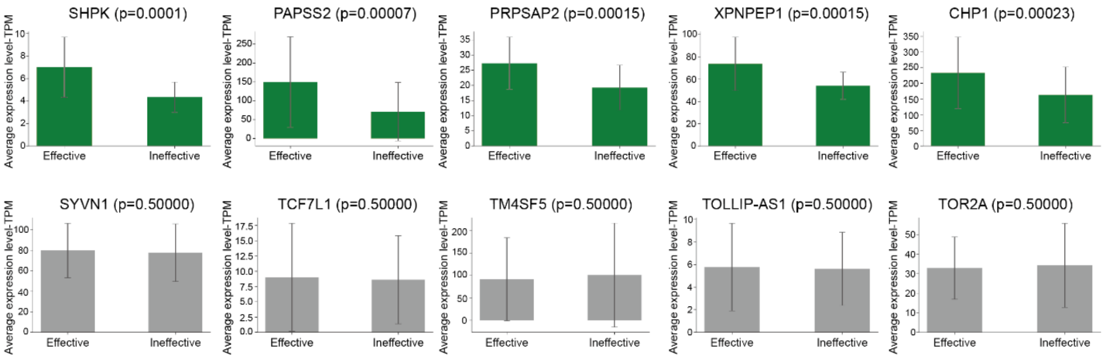

Figure S14. The retained and filtered-out genes of the Mann-Whitney U test. The illustration of Mann-Whitney U test filter. The transcription levels of the genes across all drug-sensitive and non-sensitive samples are tested by Mann-Whitney U test. Genes with p-values less than 0.1 are retained and rated (by the p-values). The top 5 figures characterize the retained genes, and the bottom 5 figures characterize the filtered-out genes. (Drug: 5-FU; cancer type: COAD and READ)

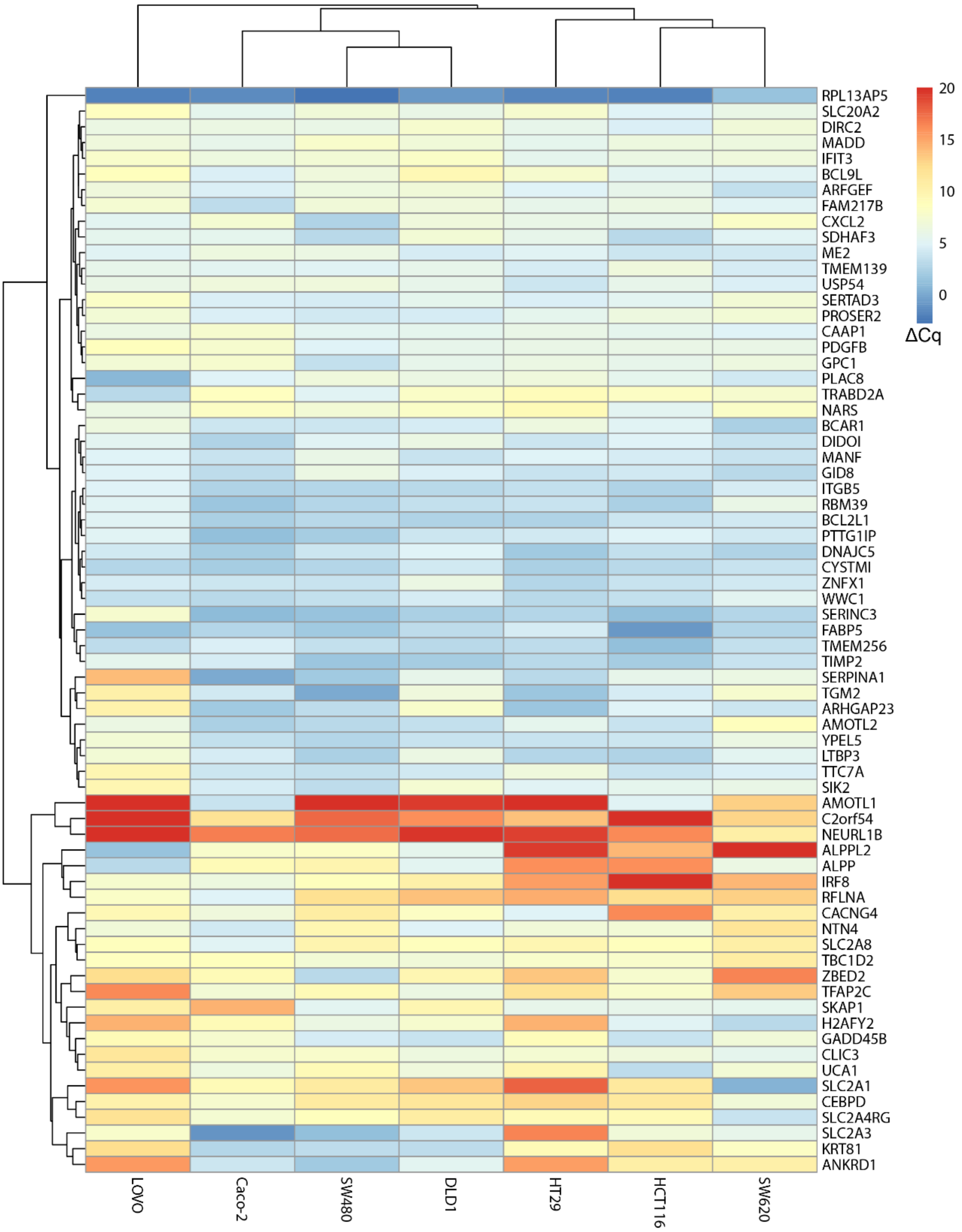

Figure S15. Gene transcription levels of the digital marker genes of cancer cell lines. The columns represent the samples (cell lines). The rows represent the digital markers (for Cisplatin, 5-FU and Paclitaxel and colorectal cancer cell line). The colors denote the transcription levels.

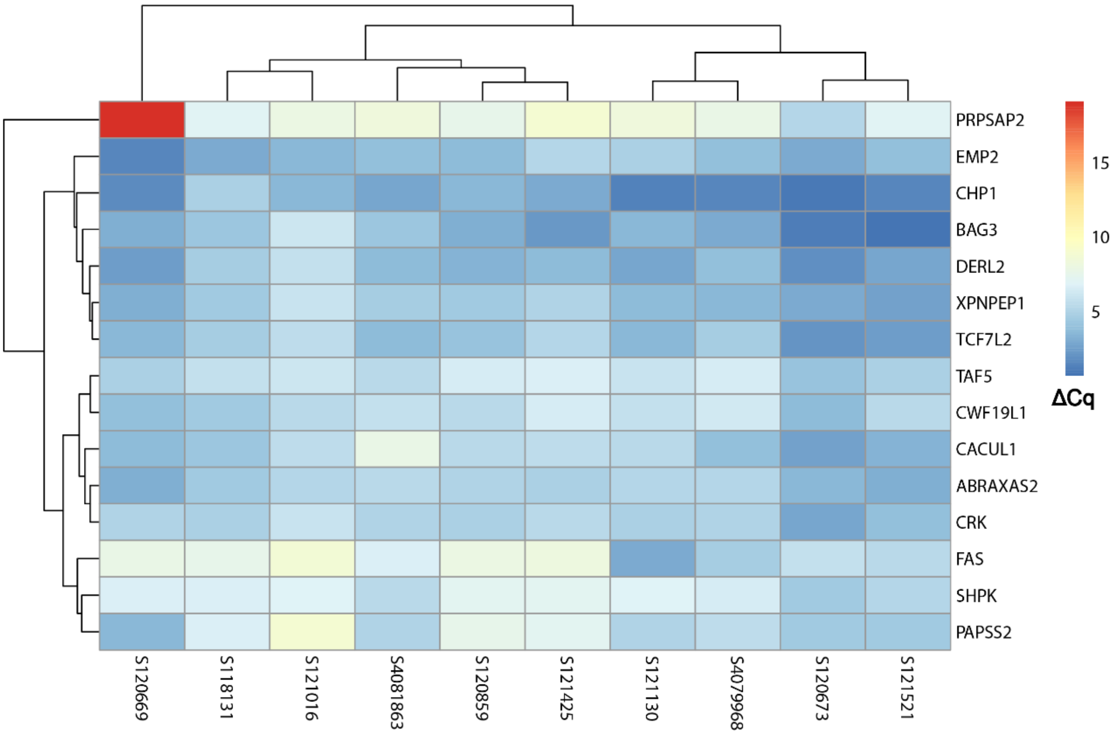

Figure S16. Gene transcription levels of the digital marker genes of patient derived tumors for effectiveness prediction of 5-FU. The columns represent the samples (tumor organoids). The rows represent the digital marker (for 5-FU and colorectal cancer tumor). The colors denote the transcription levels.

Table S1. Drug efficacy classification of prognostic data in TCGA.

In TCGA, prognostic information has four classes, CR, PR, SD and PD. In this study, we group CR, PR and SD as “effective” treatment, and PD as “ineffective” treatment.

**Considered as Effective/Ineffective**	**Treatment best response**	**Explanation**
Effective	Complete response(CR)	All target lesions disappeared without new lesions, and tumor markers were normal for at least 4 weeks.
	Partial response(PR)	The sum of the maximum diameter of the target lesion decreased by at least 30% for at least 4 weeks.
	Stable disease(SD)	The sum of the maximum diameter of the target lesion was not reduced to PR or increased to PD.
Ineffective	Progressive disease(PD)	The sum of the maximum diameter of the target lesion increased by at least 20%, or new lesions appear.

Table S2. The analyzed cancer type-drug pairs in this study.

Cell line/Patient sample	Cancer type	Drug	Dataset
Cell line	Colorectal cancer	5-FU, Cisplatin, Cyclophosphamide, Paclitaxel, Vinblastine, Docetaxel, Oxaliplatin, Irinotecan	CCLE & GDSC
	Lung cancer		
	Breast cancer		
Patient sample	Colon Adenocarcinoma (COAD), Rectum Adenocarcinoma (READ)	5-FU, Oxaliplatin	TCGA
	Lung Adenocarcinoma (LUAD)	Cisplatin	
	Stomach Adenocarcinoma (STAD)	5-FU, Capecitabine, Cisplatin	

Table S3. The digital markers for cell line drug sensitivity prediction. For most cases, the digital markers are screened by using both datasets, CCLE and GDSC. For breast cancer cell lines, the digital markers of 5-FU and Paclitaxel are screened by using CCLE only, due to a sharp decrease in performance of cross-validation using both datasets.

Cancer type	Drug	Digital markers
Colon	5-FU	ARHGDIB, TRAF5, CDC42EP3, RFLNA, CNNM4, SHC1, PAM16, CHID1, ZBED2, PAK4, CSRNP1, L1CAM, SFXN4, ZNF200, NPIPB15, C6orf203, SLC35F5, SLC18B1, SERTAD3, UPK2, CACNG4 (21 genes)
	Cisplatin	PLBD1-AS1, NOP9, MANF, IRF8, PIWIL4, GID8, SLC20A2, TSPAN3, USP54, TMEM164, KIAA0232, ATR, SLC30A1, SENP2, LINC01133, TAF4, TMEM139, CLIC3, YTHDF1, U2SURP, TMEM256, PTTG1IP, SLC2A1, DNAJC5, WWC1, KRT81,HEPH, PNKD (28 genes)
	Paclitaxel	MPP1, ALPPL2, FAM81A, PLAC8, RFLNA, POFUT1, DYNLRB1, SLC2A3, FOXP1, SBK1 (10 genes)
	Cyclophosphamide	CLDN2, SYT13, SLC6A9, ATP5MPL, CEBPD, DYNLRB1, SLC2A4RG, CNN2, ABHD12, SERPINA1, TRABD2A, RALY, BCL9L, STAU1 (14 genes)
	Vinblastine	FOLR1, PLXND1, EIF4BP6, CD74, PDGFB, PDGFA, SLC2A3, LINC00909, INTS6L, PFAS, MPP1, CTDNEP1, EGR1 (13 genes)
	Docetaxel	MPP1, SFXN5, POFUT1, GID8, DYNLRB1, SLC30A1, PGM2, SLC2A3, OSBPL2, NTN4, KLHDC2 (11 genes)
	Oxaliplatin	CEBPA-DT, CEBPD, GAD1, FBXL18, RC3H2, PDZD11, DSN1, IFIT3, CIZ1, PLBD1-AS1, FKBP4, MARCKS, DNAJC5, ABCD1, CTNNBL1, TLE1, PRKD2, CSTF1, EIF4G1, ADNP, ZNF106, FBXL8, PIK3R4, HPS5, ACTR1A, WNK4 (26 genes)
	Irinotecan	RPL34, PALD1, UBE2V1, CXorf56, TSC22D2, EEF1A2, POLR1C, ZFP64,

		HCG11, MPP1, HIKESHI, PXDC1, KLHDC2, TFAP2C, A4GALT, SPATA2 (16 genes)
Lung	5-FU	FAM3A, PDZD11, PLEKHM1, NEU1, TNFAIP2, SMPD1, MEA1, SNX2, BRPF3, NAGK, C17orf51, ACTR1A, AGK, RCN3, SPAG9, FAM50A, DENND4A, MMGT1, CYC1, PDE6D, DHX40, TMEM178B (22 genes)
	Cisplatin	IL18, MIR4435-2HG, ORAI3, NT5E, CPNE8, SQOR, CYTOR, GNG4, LIF, CPSF6 (10 genes)
	Paclitaxel	CTSZ, IL18, C19orf33, LIF, GPRC5A, FAM214B, NT5E, OSMR, SP100, MET, KRT80, GBP3, KRT18, CAV2, NCEH1, VSIR, PPARG, AHNAK, LAPTM4A, TTLL12, ALDH3B1 (21 genes)
	Cyclophosphamide	C19orf33, IL18, GPRC5A, NT5E, SYT11, CAV2, KRT80, OSMR, DFFB, VSIR, ADIRF-AS1, MYH9, AHNAK, UBE2H, MYOF, ALDH3B1, CAST, CAVIN1, ATP2A1-AS1, CTSZ, CCDC28B, MARK1, BEX1 (23 genes)
	Vinblastine	OIP5, CBX2, CAPN2, VSIR, GCDH, COQ3, NABP1, HERC2, TEDC2, BUB1B, GPRC5A (11 genes)
	Docetaxel	LIF, RUNX1, SP100, C19orf33, CAVIN3, TMCO4, GBP3, CBX2, CAV2, TTLL12, NT5E (11 genes)
	Oxaliplatin	GADD45B, RRAS, HERC2, LGALS1, MARK1, AHNAK, CAV2, SLC10A3, S100A10, 1-Mar, FAN1, RMND5A, FLII, FAM117B, MIR22HG, PXN, DRAP1, CALU, MYADM, TMEM200B (20 genes)
	Irinotecan	HKDC1, MEN1, PATZ1, PLA2G16, PDGFC, HEXB, SRSF7, PSAP, SCAF4, HNRNPUL2, MTA2, ABCC3 (12 genes)
Breast	5-FU	INTS7, CENPF, SIRPA, ZNF229, SLC25A12, SS18L2, RFX5, RAB18, GSN, JAG1, CHST14 (11 genes)
	Cisplatin	TFF3, KRT5, TFF1, CACNG4, CBR1, JAG1, TSPYL5, GREB1, MPP7, PRRT3, SOAT1, COL12A1, ACSL5, TMEM51, STC2, ABAT, ESR1, PPM1K, HOXC8 (19 genes)

	Paclitaxel	TFF1, CRYBG3, ESR1, TFF3, ABAT, GHR, RGL1, TSC1, FTLP14, STAC, TMEM116, ING1, HIST1H2BF, RACK1 (14 genes)
	Cyclophosphamide	MGST1, C19orf33, ZSCAN31, GNB4, PTK7, ZNF185, MOV10, CACNA1H, RIN2, EVC, MIR4458HG, EEF1A2, NHLRC1, HIST1H2BG, TLE1, GALC, APBB1, SLC7A2 (18 genes)
	Vinblastine	AREG, ELMO3, CDH1, ABCA12, PARD6A, POF1B, FGD1, CRABP2, EPPK1, ZNF513, TMEM191A, SLC29A4, HR, MGP, GAL, DGKE, ENPP5, SHISA2, EGLN2, RNU1-106P (20 genes)
	Docetaxel	IGFBP5, TFF3, FXYD5, FSCN1, CA12, TFF1, IGF2BP2, TRIM29, CACNG4, SYCP2, AGR3, PRR15L(12 genes)
	Oxaliplatin	TRPS1, TFF1, CACNG4, SYCP2, PRLR, GREB1, MPP7, ATF3, GAL, APOE, ABAT, ENG, PPM1K, ESR1, HOXC8, ZNF512, OPTN, PREX1, NECTIN1, LMCD1, ST6GALNAC4 (21 genes)
	Irinotecan	TFF1, CACNG4, PRLR, PPARG, JAG1, PROM1, SH3BP1, TRIL, AR, CENPV, HSPB8, NEBL, C3orf14 (13 genes)

Table S4. The digital markers for patient drug effectiveness prediction. The genes colored red are digital markers selected by different drugs for a certain cancer type. The genes colored blue are digital markers that are also cancer genes.

Cancer type	Drug	Digital markers
COAD, READ	5-FU	SHPK, PAPSS2, PRPSAP2, XPNPEP1, CHP1, TAF5, ABRAXAS2, DERL2, EMP2, CACUL1, CWF19L1, FAS, BAG3, TCF7L2, CRK, MYEOV, CTDNEP1, MICU1, SCO1, DUT, SEMA4G, MCU, ALKBH5, EXOC6, GLOD4, GLUD1 **(26 genes)**
COAD, READ	Oxaliplatin	SHPK, MYEOV, DGKA, GPRC5A, KCTD5, LINC01273, MCU, SH3PXD2A-AS1, RASL10B, EMP2, LPAR5, XXYLT1, SLCO1B3, BAG3, IER3-AS1, RHOQP1, CHM, DPY19L2P2, FUNDC2P1, CLCF1, ZXDA, NBAT1, AZGP1P1, BCAS4, XRRA1, CASP7, LINC02012, ALKBH5 **(28 genes)**
LUAD	Cisplatin	MAMSTR, DBP, C2orf70, MGARP, THADA, BAX, CNPY3, FOXO6, ISYNA1, SSBP4, CNN3, RPIA, CYP2F2P, KCND1, TEAD1 , ZMAT2, PCP4 **(17 genes)**
STAD, ESCA	5-FU	HIST3H2A, FAHD1, VCX2, SEPHS2, FIBP, ZSWIM1, PRKACB, PRR7, PQBP1, ASPHD1, PAM16, GNPTG, SCAMP3, RPP25, LINC00605, VCX3A, SEZ6L2, TSPAN5, NAXE, PFDN2, CFL1, LINC01979, KLF2P4, NMRAL1, MPV17L, RPUSD1, TUFM **(27 genes)**
STAD, ESCA	Capecitabine	GNRHR2, GRIN2D, PGM2L1, PAIP2B, HOTTIP, BCAP31P1, CETP, ABAT, DAB2, GJA4, GJA5 **(11 genes)**
STAD, ESCA	Cisplatin	IDUA, SNHG5, GSPT1, ACHE, MBD2, UBE2L3, BBOX1, LINC02041, ATP5F1A, SHROOM1 **(10 genes)**

Table S5. Overlapped genes among the 1~200 ranked genes screened independently by CCLE and GDSC. Numbers in parentheses index the genes in the lists screened by CCLE and GDSC, respectively. The genes colored red are digital markers overlapped.

Cancer type	Drug	Overlapped genes
Colon	5-FU	ARHGDIB(1,80), RFLNA(97,1), ZBED2(107,132), PAM16(109,39), CSRNP1(126,167), ZNF200(197,116)
	Cisplatin	MANF(61,102), GID8(90,65), PIWIL4(97,51), SLC20A2(101,185), USP54(122,140), TMEM164(124,165), TSPAN3(126,87), ATR(178,72)
	Paclitaxel	DYNLRB1(66,43), FOXP1(75,99), RAB11B(96,151), CEBPD(97,37), ALPP(111,16), EIF3I(124,103), FGD4(132,46), PODXL(173,68)
	Cyclophosphamide	CLDN2(1,41), SYT13(24,160), SLC2A4RG(115,70), BCL9L(196,39)
	Vinblastine	FOLR1(11,157), CD74(30,16), SLC2A3(60,128), LINC00909(93,13), RPL6P27(180,189), KRT81(185,188)
	Docetaxel	MPP1(12,49), POFUT1(50,45), GID8(62,190), SLC30A1(64,192), DYNLRB1(66,119), OSBPL2(78,115), KLHDC2(82,98), PGM2(90,16), MED22(136,146)
	Oxaliplatin	CEBPD(34,12), DSN1(118,180), FBXL18(125,5), IFIT3(139,94)
	Irinotecan	PALD1(60,193), UBE2V1(63,157), EEF1A2(118,5), MPP1(165,133)
Lung	5-FU	FAM3A(58,24), PDZD11(89,44), TNFAIP2(103,154)
	Cisplatin	KIF5C(2,91), ORAI3(9,4), SQOR(19,22), MARCKSL1(25,65), CAV2(28,66), CAV1(31,94), CTSA(34,43), MYOF(41,61), CPSF6(47,30), H3F3A(53,80), OSMR(56,38), SP100(68,166), RRAS(96,131), FOSL1(97,143), CAVIN1(104,37), GNG4(106,18),

		CSTF3(115,55), CAST(117,123), ADAMTSL5(121,108), RAB3A(123,104), NT5E(124,2), VMP1(130,127), AXL(143,100), SP140L(145,29), LGALS1(150,158), MARK1(169,48), RPS6KL1(172,60), CYTOR (181,14), CBX2(184,76), LINC00857(198,172)
	Paclitaxel	TTLL12(13,152), CBX2(29,188), GBP3(38,47), DNAJC12(41,185), CAPN2(59,190), SGMS2(181,173), IL18(199,2)
	Cyclophosphamide	CAV2(4,33), C19orf33(6,2), AHNAK(12,70), ATP2A1-AS1(16,92), MYOF(22,79), CAVIN1(32,81), AHNAK2(39,178), SGMS2(45,116), CCDC28B(46,106), CTSZ(53,97), ALDH3B1(67,75), SYT11(72,13), ADIRF-AS1 (84,59), ZNFX1(86,141), VSIR(91,52), NCEH1(94,136), NT5E(125,7), MEX3A (136,126), BEX1(140,94), CAV1(156,135), SP100(159,118), CAPN2(169,123), OSMR(175,35), MAST1(184,183), IL18(185,3), ITGA3(198,95)
	Vinblastine	CBX2(9,135), CAPN2(22,125), GCDH(40,137), BUB1B(41,184), GPRC5A(102,160), PHLDA3(152,147), NABP1(165,84), ADSL(167,170), VSIR(173,72)
	Docetaxel	CBX2(1,47), CYR61(12,192), CAPN2(30,140), TTLL12(75,63), MIR17HG(76,110), CTSZ(86,86), CTSA(109,122), SAMM50(111,170), IL6ST(113,182), MIR221(127,82), CAV2(180,56)
	Oxaliplatin	GADD45B(45,22), MARK1(48,120), LGALS1(50 ,115), CALU(124,166), PXN(139,137), DRAP1(147,136), 1-Mar(167,78), MAML3 (173,145), MYADM(183,126)
	Irinotecan	PLA2G16(95,138), MEN1(193,82)
Breast	5-FU	ESR1(12,170), INPP4B(41,100), MPP7(45,79), TFF1(48,10), TMPRSS3(70,185), RANGRF(96,166),

		TFF3(146,3)
	Cisplatin	KRT5(19,4), TFF1(23,25), ACSL5(69,198), PRRT3(125,151), GREB1(167,127), CBR1(178,69)
	Paclitaxel	TFF1(0,18), TFF3(3,3), FXYD5(17,74), TMC4(42,71), SEC63(61,135), AGR3(67,46), ABCC11(85,98), GREB1(100,99), UST(131,157), MINDY1(140,162)
	Cyclophosphamide	C19orf33(176,47)
	Vinblastine	TMEM191A(24,124), POF1B(41,68), ABCA12(44,42), SLC29A4(47,164), HR(58,185), ELMO3(76,12), EPPK1(183,103), CDH1(197,28)
	Docetaxel	TFF1(0,26), TFF3(5,3), FXYD5(9,8), ABCC11(26,121), SOAT1(56,184), CA12(58,25), AR(66,168), DEGS2(84,169), SPATS2L(97,185), SYCP2(99,53), FKBP10(136,89), IGF2BP2(149,31), LYN(158,181), ALDH3B2(165,66), CACNG4(180,52)
	Oxaliplatin	ATF3(64,155), MPP7(65,153)
	Irinotecan	PROM1(4,136), PPARG(65,111), CACNG4(142,51), SH3BP1(144,150), PRLR(153,68)

Table S6. Top 20 important genes for drug sensitivity prediction on Colorectal, Lung and Breast cancer cell lines screened by gene filters across all clinical drugs available in GDSC and CCLE.

We screened and rated the genes important for drug sensitivity prediction on Colorectal, Lung and Breast cancer cell lines toward all clinical drugs. We found some genes with high predictive importance but not contained in the oncogene list(Thomas R K et al. Nature genetics, 2007, 39(3): 347-351). The examples are TGM2, SLC2A1, NT5E, ACKR3 and TFF1.

**Colorectal cancer**	**Lung cancer**	**Breast cancer**
TGM2	NT5E	ACKR3
SLC2A1	RGS16	TFF1
UPK2	C19orf33	ESR1
CEBPD	DPYSL5	TFF3
CD74	KIF5C	GREB1
BCL2L1	KIF1A	ELF5
DYNLRB1	AXL	EMB
ARHGAP23	MIR221	DSCAM-AS1
SLC2A4RG	FZD3	CYP2T1P
TMEM139	EYA2	C19orf57
DNAJC5	NAV3	DEGS2
LBH	INSM1	SHISA2
PTTG1IP	SYP	PROM1
CLIC3	GBP3	CCDC113
BLCAP	SAMD4A	RGL1
ME2	OXTR	CACNA1H
SLC20A2	HRH1	ELOVL2
MANF	SNAP25	WASF3
ITGB5	RAB3A	ABAT
TM9SF4	SLFN12	SLC7A2

Table S7. Cancer genes among the 1~200 ranked genes screened by the gene filters (using both datasets CCLE and GDSC). Numbers in parentheses index the genes in the lists. The genes colored red are cancer genes among digital markers.

Cancer type	Drug	Cancer genes
Colon	5-FU	LATS2(34), SH3GL1(41), POT1(54), MLLT3(81), HNF1A(105), LEPROTL1(148), MECOM(175), PDGFB(176), PRKAR1A(181), TSC2(196)
	Cisplatin	ATR(11), GATA3(56), RPL5(92), NCOA1(98), CREB3L1(112), FOXP1(116), CHD4(135)
	Paclitaxel	FOXP1(8), SDC4(37), FGFR1(68), GATA3(83), MECOM(94), CREB3L1(95), MGMT(134), MNX1(173)
	Cyclophosphamide	BCL9L(12), TMEM127(35), ASPSCR1(104), POU5F1(121), FOXP1(124), RPL5(153), CHD4(166), TNC(169), CDKN2A(194)
	Vinblastine	CD74(3), PDGFB(4), SDC4(28), FGFR1(83), CHEK2(87), FOXP1(95), VTI1A(135)
	Docetaxel	SDC4(27), FGFR1(59), GATA3(75), MECOM(84), CREB3L1(85), FOXP1(114), MGMT(124), MNX1(166)
	Oxaliplatin	KAT6A(28), RBM15(32), MECOM(74), FBXO11(111), MNX1(118), NCOA1(139)
	Irinotecan	CXCR4(85), MNX1(116), NUP214(190), EWSR1(196)
Lung	5-FU	BRAF(32), MYO5A(49), NCKIPSD(52), CRNKL1(56), PTPN11(83), FLI1(120), CYLD(129)
	Cisplatin	H3F3A(25), CAMTA1(35), CXCR4(56), EGFR(92), EZH2(95), BCOR(102), BCL9L(121), ACVR1(139), MYH9(150), CNTNAP2(153), TFE3(162), IL6ST(170), TMEM127(179)
	Paclitaxel	MET(9), PPARG(16), EPAS1(31), LASP1(33), IL6ST(44), SDC4(77), EGFR(92), WNK2(93), PDGFB (105), RUNX1(112), CARD11(139), TFE3(193)
	Cyclophosphamide	MYH9(11), CAMTA1(47), EGFR(48), IDH2(62), MYB(66), EPAS1(72), IL6ST(88),

		PATZ1(101), CARD11(135), TBX3(144), SBDS(161), PPARG(167), MET(178), TFE3(183), NT5C2(196)
	Vinblastine	BUB1B(9), NTHL1(18), WDCP(32), LASP1(37), MAML2(51), TFE3(57), CAMTA1(58), PATZ1(62), TSC1(104), IL6ST(106), SBDS(118), KNL1(119), ERCC3(132), AFF4(137), PMS2(155), DDB2(158), CLIP1(183)
	Docetaxel	RUNX1(1), IL6ST(28), EGFR(37), LASP1(46), WNK2(63), MET(79), PPARG (83), CASP8(87), CBFA2T3(93), CARD11(105), TFE3(115), TNFAIP3(127), PATZ1(156), ACVR1(166), NT5C2(167), POLE(168), PDGFB (169), CAMTA1(196)
	Oxaliplatin	LMNA(40), LASP1(41), PATZ1(49), RUNX1(100), TFE3(117), WNK2(132), ACVR1(140), SBDS(151), MNX1(152), CARD11(164), IL7R(168)
	Irinotecan	MEN1(1), PATZ1(2), PBRM1(37), NUMA1(68), TGFBR2(74), LATS2(76), VHL(86), PIK3CB(92), CHD4(94), NCOA2(103), GPHN(114)
Breast	5-FU	ESR1(13), CBLC(119), MLLT6(160), FOXO3(173), PTPRK(176)
	Cisplatin	ESR1(16), SIRPA(25), CD74(62), IGF2BP2(63), MET(66), EGFR(71), MSN(80), FGFR3(102), PIM1(108), CREB3L1(117), PPARG(138), CBLC(152), ERBB2(158), AR(196)
	Paclitaxel	ESR1(23), RET(33), CXCR4(60), CREB3L1(128), ERBB2(163), GATA2(175), CBLC(176)
	Cyclophosphamide	CHCHD7(28), FGFR2(33), FAT1(99), SYK(101), ETV4(119), STAG2(126), SIRPA(152), ALDH2(168), FBLN2(181), MYB(190), ETV5(199)
	Vinblastine	CDH1(2), FGFR3(89), ACKR3(169)
	Docetaxel	IGF2BP2(6), PIM1(13), TGFBR2(20), AR(26), ACKR3(37), ESR1(43), MAML2(49), SIRPA(56), MET(110), EGFR(115), MSN(124), FGFR3(142), CREB3L1(156), CBLC(184), ERBB2(192)
	Oxaliplatin	ESR1(13), CD74(55), IGF2BP2(56),

		MET(59), EGFR(64), MSN(73), FGFR3(96), PIM1(102), CREB3L1(112), PPARG(137), CBLC(154), TGFBR2(159), ERBB2(162)
	Irinotecan	PPARG(3), AR(8), CHST11(16), ESR1(19), MAML2(27), SIRPA(31), PPM1D (33), CD74(77), IGF2BP2(79), MET(82), EGFR(86), FGFR3(116), PIM1(122), CREB3L1(132), CBLC(168)
