# Supplementary material for: Multiplex gene quantification as digital markers for extremely rapid evaluation of chemo-drug sensitivity
Source: Patterns (N Y). 2021 Sep 29;2(10):100360. doi: 10.1016/j.patter.2021.100360 (PMC8515010; doi:10.1016/j.patter.2021.100360)
Supplement: Document S2. Article plus supplemental information [file mmc5.pdf]

Patterns

Article

Multiplex gene quantification as digital markers for extremely rapid evaluation of chemo-drug sensitivity

Graphical abstract

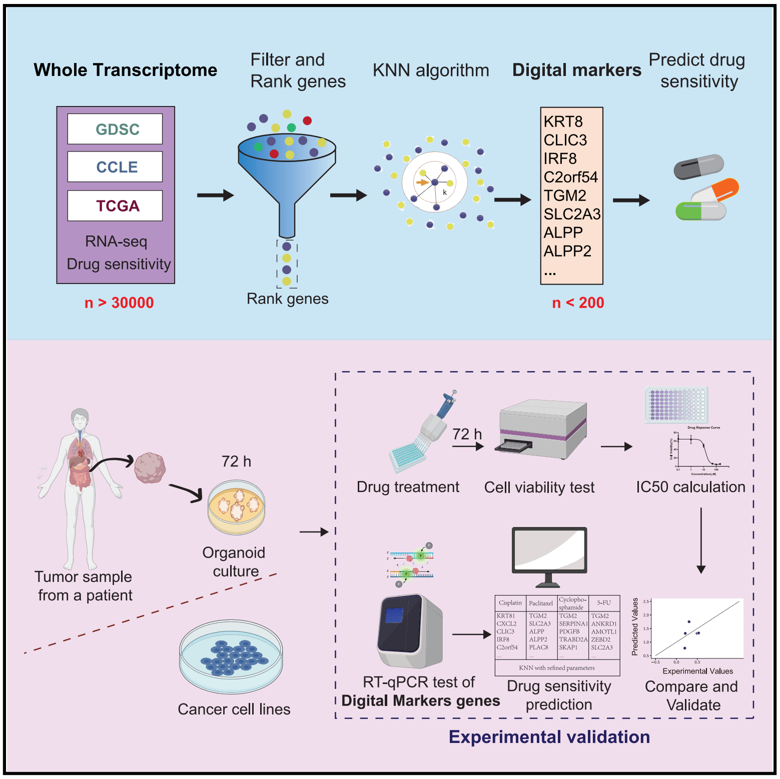

Highlights

- Non-targeted multiplex genes are screened as digital markers for drug sensitivity
- Transcription level cohort of 10s to 100s genes predicts drug sensitivity
- Digital markers are quantified using qRT-PCR within 1–3 h
- Digital markers guide extremely rapid chemo-drug uses after patient hospitalization

Authors

Jiaqi Fan, Yilin Feng, Yifan Cheng, ..., Shuzhong Cui, Weijie Zhang, Shaohua Ma

Correspondence

ma.shaohua@sz.tsinghua.edu.cn

In brief

Fan et al. developed computational algorithms to establish multiplex digital markers that can evaluate chemotherapy drugs for personalized medicine. The evaluation is completed in up to 3 h and satisfies the needs for immediate chemotherapy after patient hospitalization.

Fan et al., 2021, Patterns *2*, 100360
October 8, 2021 © 2021 The Author(s).
https://doi.org/10.1016/j.patter.2021.100360

CellPress

Patterns

CellPress
OPEN ACCESS

Article

Multiplex gene quantification as digital markers for extremely rapid evaluation of chemo-drug sensitivity

Jiaqi Fan,[1,2,3,6] Yilin Feng,[1,2,6] Yifan Cheng,[1,2] Zitian Wang,[1,2] Haoran Zhao,[1,2] Edgar A. Galan,[1,2] Quanxing Liao,[4] Shuzhong Cui,[4] Weijie Zhang,[5] and Shaohua Ma[1,2,3,7,*]
[1]Tsinghua University, Shenzhen International Graduate School (SIGS), Shenzhen 518055, China
[2]Tsinghua-Berkeley Shenzhen Institute (TBSI), Shenzhen 518055, China
[3]Institute for Brain and Cognitive Sciences (THUIBCS), Tsinghua University, Beijing 100084, China
[4]Department of Abdominal Surgery, Affiliated Cancer Hospital and Institute of Guangzhou Medical University, Guangzhou 510095, China
[5]Department of Oncology, The First Affiliated Hospital of Zhengzhou University, Zhengzhou 450052, China
[6]These authors contributed equally
[7]Lead contact
*Correspondence: ma.shaohua@sz.tsinghua.edu.cn
https://doi.org/10.1016/j.patter.2021.100360

THE BIGGER PICTURE In clinical cancer medicine, many patients require immediate chemotherapy after hospitalization. Current administrations for precision drug uses are limited in evaluation speed, including genomic sequencing and tumor organoid evaluation. An extremely rapid evaluation protocol is in high demand to realize drug recommendation within a few hours after tumor sampling. In this work, we have proposed an approach for extremely rapid and personalized drug recommendation.

1 2 3 4 5 **Development/Pre-production:** Data science output has been rolled out/validated across multiple domains/problems

SUMMARY

Current administrations for precision drug uses are limited in evaluation speed. Here, we propose the use of multiplex gene-based digital markers for the extremely rapid personalized prediction of individual sensitivity to cancer drugs. We first screen the transcriptional profiles by applying two to three gene filters and scoring genes by their impact on drug sensitivity and finalize the gene lists by K-nearest neighbors cross-validation. The digital markers are cancer type dependent, are composed of tens to hundreds of gene expressions, and are rapidly quantified by reverse transcription quantitative real-time PCR (qRT-PCR) within 1–3 h after tumor sampling. The area under the receiver operating characteristic curve reached 0.88 when testing the performance of digital markers on organoids derived from colorectal cancer patient tumors. The algorithm and corresponding graphic user interface were developed to demonstrate the promise of digital markers for extremely rapid drug recommendation.

INTRODUCTION

Cancer is an extremely intractable disease, due to its dynamic, metastatic,[1] and heterogeneric[2] properties. Therefore, there is no standard therapeutic regimen for most cancer types, and even for the same cancer type, effective therapies for different tumors at different developmental stages are highly variable due to inter- and intra-tumor heterogeneity.[2–4] Apart from targeted therapy that can be precisely chosen by detecting known gene mutation(s) or identifiable biomarkers, most chemotherapies are empirical. Precision or personalized drug uses are expected to improve cancer prognosis by increasing the rate of effective treatment, reducing the magnitude of side effects, and avoiding the waste of a therapeutic window.

In recent years, the availability of multi-omics big data and data mining techniques has enabled us to decipher the highly complicated interactive networks among the genomics, transcriptional identities, and underlying therapeutics.[5,6] Several public datasets have been established to include genomic, transcriptomic, and drug responsive cohorts for cancer cell lines, including the Genomics of Drug Sensitivity in Cancer (GDSC)[7–9] and the Cancer Cell Line Encyclopedia (CCLE),[10,11] and for cancer patients, such as The Cancer Genome Atlas (TCGA).[12]

This is an open access article under the CC BY license (http://creativecommons.org/licenses/by/4.0/).

Many computational algorithms and multiplex models have been developed to decipher the connection between multi-omics information and drug sensitivity. For example, linear prediction models, including univariate regression, multivariate regression, and elastic net, have been used to select genomic predictors for anticancer drug sensitivity.[13] Network-based approaches have been attempted to integrate heterogeneous information and learn low-dimensional feature vectors to predict drug-target interactions,[14] as well as identify biomarkers for drug selection using protein-protein interaction networks.[15] Deep neural networks can integrate the information of gene mutations and transcriptional alterations and improve the prediction of drug responses for large clinical cohorts.[16,17] The accessibility to such public datasets has expanded the scope of computation-based precision medicine. Sanchez-Vega et al. first proposed oncogenic signaling pathways as a tumor classification strategy, stratified tumors into 64 subtypes, and suggested new therapeutic opportunities by studying the large cohort in TCGA.[18] Recently, Paull et al. discovered common regulatory knobs in gene networks across different tumor types and proposed a new mechanism of tumor classification that is independent of organ specification.[5] A similar finding was further examined in non-tumor diseases.[6] Computational models have also reported successes in probing drug tolerance transition[19] and synergistic drug effects[20] in cancer cells based on their transcriptional data. It is a fact that most tumors lack specific mutations for targeted therapy; however, they possess transcriptional alternations. The aforementioned studies support the investigation of transcriptional identities as markers for therapeutic prediction and personalized drug use.[21,22]

However, the current RNA sequencing (RNA-seq)-based big data mining has limitations in temporal efficiency. The RNA-seq operation takes 4–6 weeks and requires costly gene sequencers.[23,24] In many clinical practices, chemo-drug administration is expected immediately after patient hospitalization.[25] Therefore, extremely rapid drug evaluation becomes fundamentally important, but it is beyond the capacity of any existing methods.

In this work, we aim to establish an approach that recommends drug use within a few hours after accessing patient tumors. It starts from computational screening of the whole transcriptome to build small gene libraries for prediction of drug response. The transcriptional profiles of screened multiplex gene libraries compose digital (virtual) markers for prediction of chemo-drug sensitivity, which is analogous to the biomarkers used in targeted drug selection (see Table 2). The quantitative assessment of digital markers provides precision treatment choices for a given sample. We model the use of digital markers in clinical practice by quantifying transcriptions of screened digital markers using qRT-PCR. A computational algorithm is provided that converts the transcriptional quantification to drug recommendation (Video S1).

RESULTS

Gene filtering and prediction of drug sensitivity using a KNN model

A computational model was built to establish digital markers by screening genes to compose the multiplex gene library that would predict the response of a given sample to a certain drug (Figure 1A). Bio-data from both cancer cell lines and cancer patients (i.e., tumor tissues) can be applied to this model. We gathered the RNA-seq data and drug sensitivity data, characterized by the half-maximal inhibitory concentration (IC_{50}) (see Table 2), from the GDSC and CCLE datasets to construct the training and testing cohorts for cancer cell lines. For patient tumors, the bio-data were constructed from the RNA-seq data and prognostic information, i.e., the classification of drug responses in TCGA (Table S1).

We first applied different filters for cancer cell lines and patient tumors due to their difference in drug sensitivities (Figure 1A). However, the processing logic was the same: genes beyond a certain transcription level and with a stronger correlation to drug sensitivity were retained after being filtered (Figures 1B and 1C). After gene filtering, the genes were scored, rated, and used to compose the gene candidate list. For cell lines, as the RNA-seq data from CCLE and GDSC do not match well (Figure S1C), in order to make full use of the data and improve robustness, we performed gene filtering on both datasets separately and synthesized the scores of genes as the rating criteria to finalize the list of gene candidates (see experimental procedures for details).

The second step was K-nearest neighbors (KNN) cross-validation and establishment of digital markers (Figure 1A). The KNN algorithm determined the top m_0 genes to compose digital markers and the parameter k_0 to reach the highest area under the receiver operating characteristic (ROC) curve (AUC) values of receiver operating characteristic curves (see experimental procedures for details).

The last step was prediction of drug sensitivity using the KNN model. For cell lines, drug sensitivity data are continuous; KNN regression was therefore used to predict the Z scores of the IC_{50} values. For patient samples, drug sensitivity values were provided as labels according to treatment outcomes; we therefore used the KNN classification here.

In "cross-validation of digital marker-based prediction of drug sensitivity on cancer cell lines and patient tumors," we show the digital markers and cross-validation performances for several cancer type-drug pairs (Table S2) on the datasets of cancer cell lines and patient samples (cancer cell lines, CCLE and GDSC; cancer patient samples, TCGA). In "qPCR-based extremely rapid prediction of drug sensitivity," we describe the gene expression value conversion and experimental validation. In "digital markers accurately predict drug sensitivity of cell lines and patient tumor organoids," we perform experimental validation of the prediction of drug sensitivity using cell lines and patient tumor-derived organoids cultured in the laboratory.

Cross-validation of digital marker-based prediction of drug sensitivity on cancer cell lines and patient tumors

The transcriptional profiles and drug sensitivity of cancer cell lines and patient tumors were shown to be cancer type specific (Figures S1 and S2; Table S2). Correspondingly, we built the prediction models in a cancer type-specific manner. For each cancer type, a set of digital markers was established by computational screening of individual drugs. Figure 2A shows the comparison of the whole transcriptome and the screened digital markers in when evaluating the treatment efficacy of fluorouracil (5-FU) in colorectal cancer cell lines in the GDSC database. The

CellPress
OPEN ACCESS

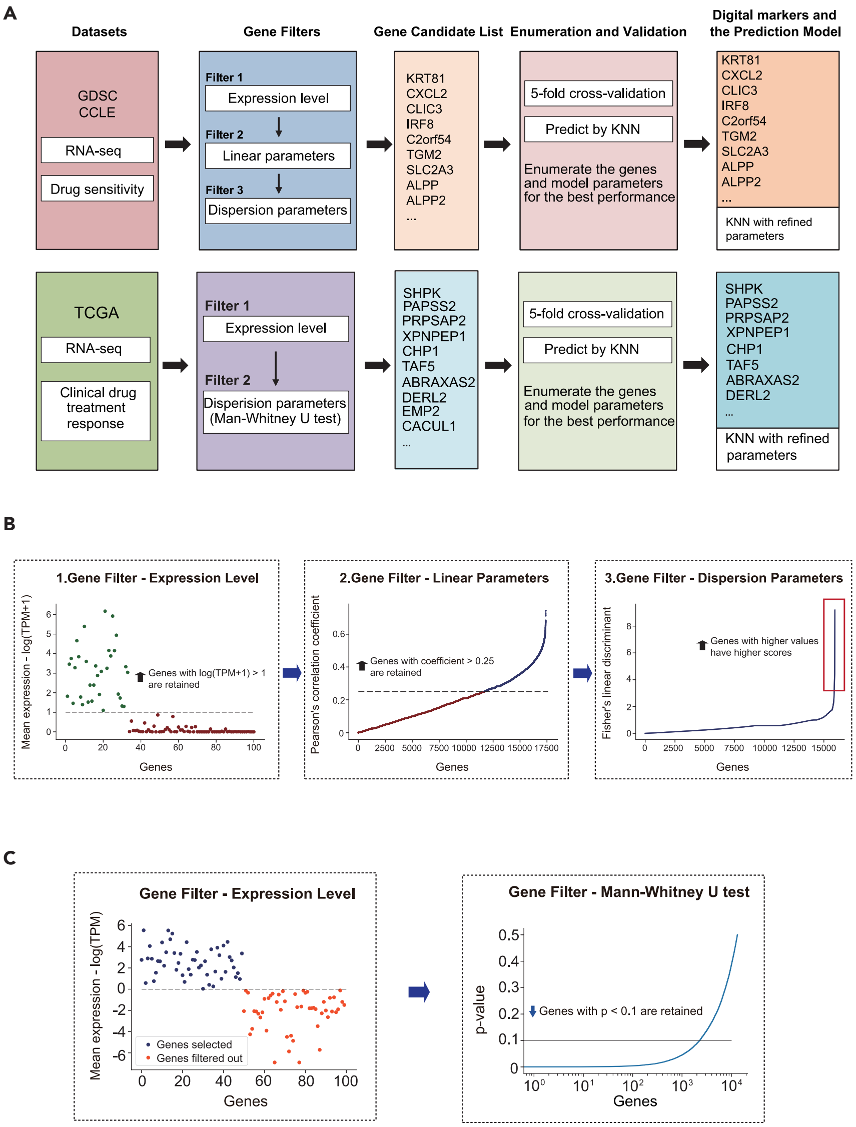

Figure 1. Computational model to establish multiplex gene-based digital markers

(A) The flow charts of the computational approach for prediction of drug sensitivity in cancer cell lines (upper) and patient samples (lower).

(B) The three gene filters used in screening the digital markers for cancer cell lines.

(C) The two gene filters used in screening the digital markers for patient samples.

cell lines are indicated by the yellow dots and mapped to the two-dimensional (2D) space by principal-component analysis (PCA). The background colors represent the drug sensitivity of 5-FU, rendered by KNN classification in the 2D feature space. The prediction using the digital markers aggregates the drug-sensitive and non-sensitive cell lines into two distinct groups in the feature map, whereas the whole transcriptome group (all genes) fails to prove its prediction ability. It suggests that the digital marker-based prediction can better distinguish the drug-sensitive and non-sensitive cell lines.

The ROC curves of cross-validation of prediction of drug sensitivity are shown in Figures 2B and S3–S8 and Figures 2C and S9 for cancer cell lines and patient tumors, respectively. Table 1 summarizes the evaluation scores in Figures 2B and 2C. For both cancer cell lines and patient tumors, the AUC for cross-validation reached scores above 0.9.

To test the generalizability of our model, we performed "cross-prediction" for cell lines by training the KNN model in one dataset (GDSC/CCLE) and predicting drug sensitivity in the other dataset (CCLE/GDSC). We compared the performance of three prediction methods: (1) performing gene filtering on both GDSC and CCLE and predicting drug sensitivity by 5-fold cross-validation in GDSC and CCLE separately, (2) using GDSC/CCLE as the training dataset and predicting drug sensitivity by 5-fold cross-validation in the same dataset, (3) "cross-prediction," using GDSC/CCLE as the training dataset and predicting drug sensitivity by 5-fold cross-validation in CCLE/GDSC. Except for a few instances, the performance of the cross-prediction method was satisfactory, reaching accuracy grades similar to that of the controls (Figures 2B and S3–S8). The digital markers of different cancer type-drug pairs are finalized by method 1 and are listed in Tables S3 and S4 for cancer cell lines and patient samples, respectively. Most digital marker genes were proven essential for the progression in certain cancers. For example, in the digital markers of patient samples (Table S4), the epithelial membrane protein 2 (EMP2) occurred in patient samples of colorectal cancer in response to 5-FU or oxaliplatin administration (Table S4), representing a novel therapeutic target for endometrial cancer stem cells,[26,27] while the fact that the BAG cochaperone 3 (BAG3) occurred in colorectal cancer cell lines exposed to 5-FU or oxaliplatin correlates with apoptosis in treated colon cancer cell lines[28] and chemoresistance.[29]

Venn diagrams of cancer genes[30] and digital markers of cancer cell lines and tumors show both similarity and distinction between digital marker genes and cancer genes (Figures S12 and S13; Table 2). Many screened genes have oncotherapeutic implications. For example, the Fas cell surface death receptor is related to apoptosis,[31] activating T cell killing,[32] and resistance in tumor-immune conflict in colorectal cancer,[33] while the BCL2-associated X, an apoptosis regulator, is key to apoptosis[34] and p53 transcription pathways in lung cancer[35] and also correlates with chemoresistance to cisplatin.[36,37]

We also analyzed the robustness of the digital markers and the top 200 genes filtered by the 2 datasets, respectively. We compared the top 200 genes filtered by CCLE and GDSC,

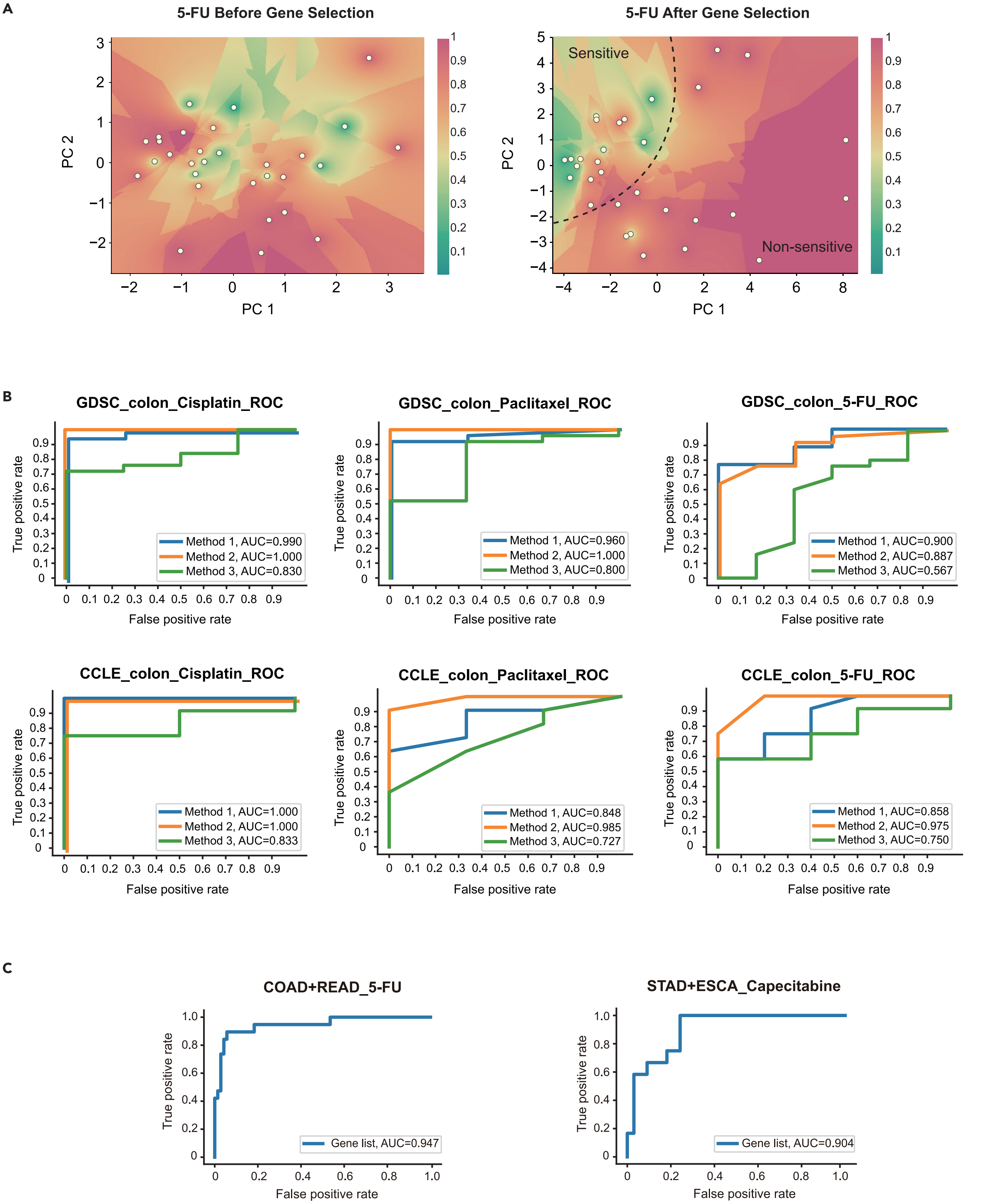

Figure 2. Digital marker-based prediction of drug sensitivity on GDSC and CCLE datasets

(A) The two-dimensional PCA dimensionality reduction maps of colorectal cancer cell lines. The digital marker separates the sensitive and non-sensitive cell lines to 5-FU, whereas the complete RNA-seq data fails to separate the two classes. The color represents the drug sensitivity of 5-FU, rendered by KNN classification

(legend continued on next page)

Table 1. Evaluation scores of the cross-validation performance

Drug and sample	Accuracy	Precision	Recall	Specificity	F1 weighted	AUC
Cisplatin, colorectal cancer cell line in GDSC	0.931	0.960	0.960	0.750	0.931	0.990
5-FU, colorectal cancer cell line in GDSC	0.839	0.833	1.000	0.167	0.788	0.900
Paclitaxel, colorectal cancer cell line in GDSC	0.929	1.000	0.920	1.000	0.936	0.960
5-FU, COAD, and READ in TCGA	0.900	1.000	0.526	1.000	0.887	0.839
Capecitabine, STAD, and ESCA in TCGA	0.756	0.737	0.167	0.970	0.697	0.904

This table lists the evaluation scores of cross-validation of prediction of drug sensitivity for colorectal cancer cell lines and patient tumors (the same instances as in Figures 2B and 2C). For cancer cell lines, the threshold of the Z score was -1.0 to distinguish sensitivity and non-sensitivity.

individually, and confirmed the overlapped genes (Table S5). For example, there were 30 overlapped genes in the top 200 genes for lung cancer cell lines and cisplatin and 26 for lung cancer cell lines and cyclophosphamide. For other cancer type-drug pairs, the overlapped genes were fewer in number, probably due to the limited data availability in the two datasets for training. It also implied the importance of data accessibility in proceeding with computation-based precision medicine.

The feasibility of gene screening as we demonstrated was further proved by analyzing the biological representations of some screened genes. For example, trefoil factor 1 (TFF1), a small cysteine-rich protein that is frequently expressed in breast tumors under estrogen control treatment, is highly related to the migration of breast cancer cell lines;[38,39] trefoil factor 3 (TFF3) is a predictive biomarker of endocrine response in breast cancer[40] and is associated with breast cancer phenotypes.[41] TFF1 and TFF3 overlapped frequently among the top 200 genes for breast cancer cell lines and 8 cancer drugs used in our study. Similarly, BCL9L plays a critical role in stem cell maintenance in epithelial homeostasis and carcinogenesis through the canonical Wnt signaling pathway in colon cancer.[42] It is apparent that the overlapping genes were either known cancer therapeutic targets or their role is otherwise worth further exploration.

qPCR-based extremely rapid prediction of drug sensitivity

qRT-PCR can be used to quantify the transcription levels of small gene libraries (e.g., containing tens to hundreds of genes) within a few hours, thus satisfying the needs of nearly instantaneous or point-of-care evaluation. To prove the concept, we first investigated the conversion of gene transcriptional values acquired by qRT-PCR (as the model testing data) to TPM (transcripts per million, see Table 2) values by RNA-seq (as the model training data). The principles of qRT-PCR[43] and RNA-seq[23] suggest that the $\triangle$Cq values (the difference of quantification cycles between the target genes and the reference gene) in qRT-PCR are linearly correlated to log(TPM).[44]

We first demonstrated this conversion on the colorectal cancer cell line SW620 by using RNA-seq and qRT-PCR data acquired in our laboratory. log(TPM) was replaced with log(TPM + 1) to avoid the error when TPM = 0 and GAPDH was set as the reference gene. Figure 3A shows a linear fit of the screened digital marker genes. Using RNA-seq data from the GDSC database resulted in a nearly identical graph (Figure 3B), demonstrating a robust conversion between qRT-PCR and RNA-seq data. The fitted parameters were then imported to the computational model with an embedded conversion formula $\log(\mathrm{TPM} + 1) = -0.3995 \times \triangle \mathrm{Cq} + 5.6974$ (to convert $\triangle$Cq to TPM).

To experimentally validate the use of digital markers, half of the cells were treated with drugs of serially increased concentrations and their viability rates to obtain the IC_{50} values were tested (Figures S10 and S11). The other half of the cells were lysed and used in the qRT-PCR quantification of the digital marker genes. The gene transcription values were then imported into the computational model. The predicted drug sensitivity values were compared with the experimental values for validation.

Digital markers accurately predict drug sensitivity of cell lines and patient tumor organoids

Seven colorectal cancer cell lines (HCT-116, HT-29, SW620, SW480, Caco-2, LoVo, and DLD-1) were cultured and administered three chemotherapy drugs, cisplatin (Figure 4A), paclitaxel (Figure 4B), and 5-FU (Figure 4C). The experimental and the predicted Z scores of the IC_{50} values presented nearly linear and positive correlations. The wrong prediction is marked by a cross in Figure 4B.

For drug effectiveness in cancer patients, clinical tracking and prognosis of cancer chemotherapy take a few years to accomplish. Therefore, to shorten the course of validation, we used patient-derived organoids to recapitulate gene expression and mimic the responses of their parental tumors.[15] Tumor organoids have been proven to recapitulate tumor heterogeneity and personalized responses to chemotherapies with the prediction accuracy generally higher than 90%.[45]

in the two-dimensional feature space. The color red refers to a higher probability of non-sensitivity; green refers to a higher probability of sensitivity. For KNN classification, k = 7; IC_{50} Z score threshold = -0.6.

(B) The ROC curves for cross-validation of prediction of drug sensitivity for colorectal cancer cell lines to cisplatin, paclitaxel, and 5-FU. The three curves in each plot refer to filtering and scoring genes on both datasets (method 1), filtering and scoring on an identical dataset (method 2), and filtering and scoring on the different dataset (method 3). IC_{50} Z score threshold = -1.0. The upper row shows the cross-validation testing results for GDSC, and the lower row for CCLE. For more cancer type-drug pairs, see Figures S3–S8.

(C) The ROC curves for cross-validation of prediction of drug sensitivity for patient samples. COAD (colon adenocarcinoma), READ (rectum adenocarcinoma) and 5-FU, STAD (stomach adenocarcinoma), ESCA (esophogeal carcinoma), and capecitabine are shown. For more cancer type-drug types, see Figure S9.

Table 2. The terminology table

Terminology	Abbreviation	Meaning
Digital marker	–	the genes (and their transcription quantification) screened to predict drug sensitivity for each cancer type-drug pair
Gene list	–	the list of digital marker genes
Gene library	–	the assembly of gene lists or all drug candidates for a certain cancer type
Chemo-drug	–	the non-targeted cancer drugs
Cancer gene	–	genes in the COSMIC Cancer Gene Census
Gene filter	–	the filters used to screen digital markers
Half maximal inhibitory concentration	IC_{50}	IC_{50} is a measure of antagonist drug potency in pharmacological research; IC_{50} values represent the concentration of a drug that is required for 50% inhibition *in vitro*
Transcripts per million	TPM	A normalized quantification of transcription of RNA-seq results

Therefore, we used tumor organoids to approximate patient tumor gene expression and drug responses. After *in vitro* culture for 4–5 days, a group of tumor organoids were treated with different concentrations of 5-FU for 72 h to obtain the IC_{50} values (Figure S11). Another group was lysed and used for qRT-PCR. The experimentally measured drug sensitivity, represented by the IC_{50} values, and the predicted responses by the computational model, were compared.

Figure 4D shows the 2D PCA dimensionality reduction map of the training data and the experimental data. The squares and circles are the training data possessing the effective and ineffective treatment outcomes. The blue and red stars are the experimental testing data of the effective and ineffective cases. The background colors are filled by the probability estimation (the probability to be ineffective) of KNN classification in the 2D feature space, green for effectiveness and red for ineffectiveness. From left to right, the black dashed lines separate the space into "effective zone," "intermediate zone," and "ineffective zone."

From the organoids derived from ten patient tumors we observed that 5-FU administration proved effective (IC_{50} < 100 μM) in four cases and ineffective (IC_{50} > 100 μM) in six cases. Figure 4E shows the experimental and predicted values for the ten tumor samples. The prediction probability was positively correlated with the experimentally measured IC_{50} values (in log form), except for two false-positive predictions (Figures 4D and 4E); the AUC score of the experimental validation reached 0.88 (Figure 4F).

DISCUSSION

In conclusion, we have proposed and validated a multiplex gene-based digital marker system to realize extremely rapid evaluation of drug sensitivity. For potential clinical practice, we have suggested a pipeline of standard uses involving tumor sampling, RNA extraction, qRT-PCR (or potentially gene chip) quantification, and intelligent readout (i.e., drug recommendation).

Based on our model validation on organoids derived from patient tumors, we propose a clinical application pipeline for extremely rapid and personalized drug recommendation (Figure 5). We will screen gene libraries, establish digital markers for all the common cancers and their clinical therapeutic drugs, and construct a user-friendly algorithm. When a patient is hospitalized, the doctor should obtain a sample of the tumor (e.g., via surgery, gastrointestinal endoscopy, or biopsy) to extract the transcriptomic materials. The transcriptional profile of the predictive digital marker should be then quantified using qRT-PCR and input to the prediction algorithm to be processed. The algorithm then outputs the predicted drug recommendation. The whole process can be completed within 1–3 h post sampling, satisfying the clinical need for immediate administration of chemotherapy following hospitalization. The process can be further improved by developing customized multiplex gene microfluidic chips that can quickly quantify transcriptomics in one small and portable device.

By using our customized computational model comprised of gene filters and a KNN algorithm, we have demonstrated the construction of small gene libraries containing digital markers that can best predict drug sensitivity. For a given cancer type, a small group (e.g., 10–20) of genes can predict a patient's response to a common chemotherapy drug; a larger library (e.g., 50–200) of genes can predict the effects of several common drugs used in clinical practice to a specific cancer type. Therefore, it may be feasible to establish gene libraries that cover most clinical chemotherapy drugs and use them to construct cancer type-specific digital markers.

Our model screened many genes possessing important drug predictive abilities (Tables S6 and S7). Most of these digital marker genes are not yet included in the recognized cancer gene list[30] but have been repeatedly reported in the literature because of their importance in cancer progress or therapy. For example, the screened genes, such as EMP2 and BAG3, have become prevalent targets for cancer therapeutic studies, which have been discussed in the Results, but they were not included in the current cancer gene list.[30] The double check from both molecular biology study and computational study may guide the exploration of unspecific genes as targets for drug development and personalized therapy.

Unlike traditional biomarkers that have defined biological functions, (e.g., proteins, mRNA, or exosomes) digital biomarkers do not have a defined biological significance. However, experimental and computational evaluation support their powerful predictive capacity for applications such as drug sensitivity. Further studies on large datasets may allow us to look into the black box. The strategy of establishing digital virtual markers may also reduce the cost and shorten the time course of new biomarker development.

Limited data accessibility, high diversity, and lack of standardization in biomedicine are common issues for computational-based diagnosis and prognosis. Although this panorama is quickly changing, learning how to extract useful information from "small" data remains a valuable goal to achieve in the

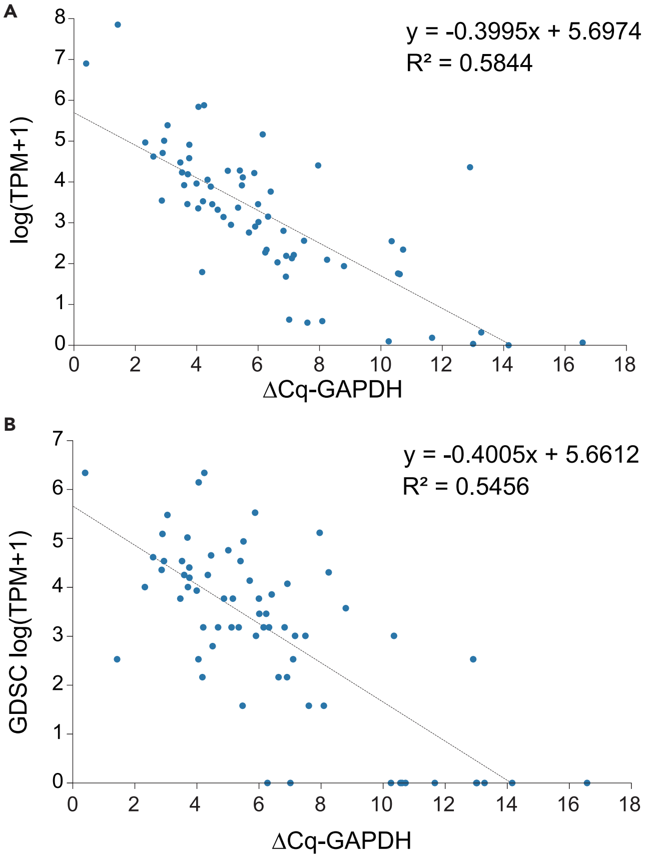

Figure 3. Comparison between △Cq and TPM for gene transcription quantification

The fitted linear relationship between log(TPM + 1), derived from RNA-seq of the cancer cell line SW620 from (A) laboratory experiments and (B) the GDSC dataset, and △Cq measured by laboratory qRT-PCR. The reference gene for qRT-PCR was GAPDH.

medium term. This would allow us extract information from much of the published literature on cancer biology to pretrain algorithms on the resultant public datasets.

We now discuss the limitations of this study. First, the training datasets from either cancer cell lines or patient tumors were limited and inconsistent. This is because these data are compiled from multiple different laboratories and patients. In a future where biobank data are standardized and available for most common cancer types, the predictive power of digital markers will be expanded and its accuracy improved. Second, the algorithm we used could be further improved by inputting prior knowledge (e.g., known biological pathways and drug-protein interactions) which increases the complexity of the model but is expected to increase its accuracy as well. Third, a recent report[5] classified tumors by master regulator proteins in their pathways after finding their similarities across different cancer types. This classification strategy may be integrated into the current digital marker approach for improved prediction scope and accuracy. In all, this study introduces the concept of a "digital marker" for drug sensitivity, but its practical uses require further studies to improve and validate.

This study aims to shorten the time from patient tumor sampling to the start of administration of an effective treatment. We therefore adopted a computational-based strategy to reduce the library of genes to construct the predictive digital markers. Other reported state-of-the-art methods can predict drug sensitivity using RNA-seq data or the whole transcriptome as the input for the algorithm. However, this results in a longer time before obtaining a prediction. Therefore, the direct comparison of this study with those different algorithms was not performed, as it can be misleading. The main focus of our method was on providing rapid recommendations. Nonetheless, our method provides a delightful solution toward extremely rapid evaluation of personalized drug sensitivity, which might inspire more computational work to serve this purpose with improved robustness and accuracy.

EXPERIMENTAL PROCEDURES

Resource availability

Lead contact

Further information and requests should be directed to and will be fulfilled by the lead contact, Shaohua Ma (ma.shaohua@sz.tsinghua.edu.cn).

Materials availability

This study did not generate new unique reagents.

Data and code availability

Data and python codes of prediction of drug sensitivity and the graphic interface are available from the GitHub repository at https://github.com/jqfan77/cancer_digital_marker.git.

Any additional information required to reanalyze the data reported in this paper is available from the lead contact upon request.

Tumor sampling

Patient tissues for tumor fabrication of organoids were collected under the approval of The First Affiliated Hospital of Zhengzhou University Ethics Committee and The Affiliated Cancer Hospital & Institute of Guangzhou Medical University Ethics Committee, following both national and local regulations. Tissues were stored in "ready-to-use" solution (Aqix, UK) with 100 μg/mL primocin (InvivoGen, USA) and transported to the laboratory under incubation on ice.

Culture of cell lines

The colorectal cancer cell lines presented in this study, including HCT-116, HT-29, SW620, SW480, Caco-2, LoVo, and DLD-1, were provided by the Peter E. Lobie Group at the Tsinghua Shenzhen International Graduate School (SIGS). Cell lines SW620 and SW480 were grown in DMEM medium supplemented with 10% FBS (v/v) (Gibco, USA) and 1% penicillin/streptavidin (v/v) (Gibco). DLD-1 was grown in RPMI medium with 10% FBS and 1% penicillin/streptavidin. HCT-116 and HT-29 were grown in McCoy's 5A medium with 10% FBS and 1% penicillin/streptavidin. LoVo was grown in DMEM/F12 medium with 10% FBS and 1% penicillin/streptavidin. All cell lines were maintained at 37°C in a humidified incubator at 5% CO_2.

Tumor dissociation and organoid culture

Tumor tissues were washed with cold 1× PBS with 5% penicillin/streptavidin (v/v) (Gibco) and cut into sub-millimeter sized small pieces. Tissues were then digested by 1 mg/mL collagenase IV (Gibco), 0.1 mg/mL DNase I (Sigma-Aldrich, USA), 0.1 mg/mL dispase type II (Sigma-Aldrich), 100 μg/mL primocin (InvivoGen) and 1% FBS (v/v) in DMEM/F12 medium on an orbital shaker at 37°C, incubated for 20–40 min. After tissue digestion, dissociated cells, cell clusters, and microtissues were passed through a 100-μm cell strainer (Corning, USA). Then the dissociated cells and cell clusters were resuspended in DMEM/F12 and centrifuged at 1,000 rpm for 5 min.

The cells dissociated from tumor tissues were suspended in Matrigel (Corning) at a density of 10^8 cells/mL. Organoids were formulated by following the reported protocol developed by Jiang et al.[46] Organoids were cultured in medium containing 20% FBS (v/v), 1% penicillin/streptavidin, 1× GlutaMAX

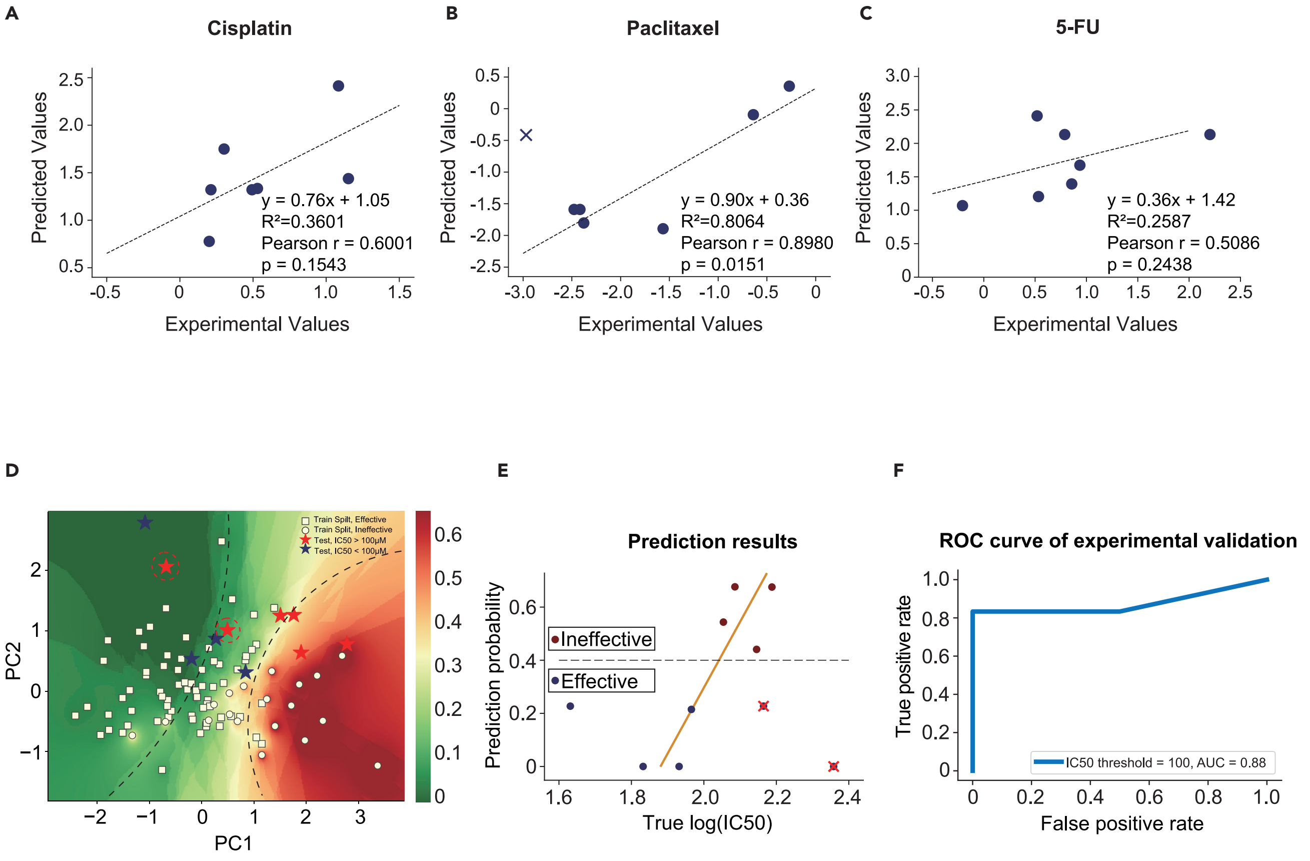

Figure 4. Experimental validation of digital marker-based prediction of drug sensitivity on cell lines and patient sample-derived tumor organoids

(A–C) Comparison of the predicted sensitivity and the measured sensitivity of (A) cisplatin, (B) paclitaxel, and (C) 5-FU, tested on seven colorectal cancer cell lines. The axis values are the Z scores of the IC_{50} values for each drug. The cross in (B) shows the wrong prediction. The seven cell lines are HCT-116, HT-29, SW620, SW480, Caco-2, LoVo, and DLD-1. Functions, R^2, p values of linear regression, and the Pearson correlation coefficient r (the square root of R^2) are shown.

(D) The two-dimensional PCA dimensionality reduction map of the training cohort and the testing cohort. The squares and circles are the training data possessing effective and ineffective treatment outcomes. The blue and red stars are the testing data possessing effective and ineffective cases. The background color is filled by probability estimation of KNN classification in the two-dimensional feature space, green for effectiveness and red for ineffectiveness. For KNN classification, k = 30. Drug used here, 5-FU; cancer type, COAD and READ.

(E) Comparison between the predicted and measured drug effectiveness. The x axis shows the experimentally measured $\log_{10}(IC_{50})$ values for tumor-derived organoids. The y axis values show the probability of drug ineffectiveness, which are expected to correlate positively with the x axis values. The blue dots are effective cases, whereas the red dots are ineffective cases. The wrong predictions are indicated by the red crosses. Drug used here, 5-FU; cancer type, COAD and READ.

(F) The ROC curve for the predictive performance of drug effectiveness using tumor-derived organoids (AUC = 0.88).

supplement (Thermo Fisher Scientific, USA), 1× B-27 supplement (Thermo Fisher Scientific), 1.25 mM N-acetylcysteine (Sigma-Aldrich), 100 ng/mL noggin (MedChemExpress [MCE], USA), 100 mg/ml R-spondin 1 (MCE), 5 ng/mL EGF (R&D, USA), 5 mM nicotinamide (Sigma-Aldrich), 5 μM Y-27632 (R&D), 100 μg/mL primocin (InvivoGen) and 1× FibrOut (CHI Scientific, USA) in DMEM/F12 medium. The medium was changed every 2 days.

Use of datasets GDSC, CCLE, and TCGA

We obtained the transcriptional and drug sensitivity (or prognosis) data from the public datasets. The GDSC data were downloaded from https://www.cancerrxgene.org/. We used data from GDSC2, a newer version of GDSC containing 809 cell lines and 198 compounds, in this study. The CCLE data were downloaded from https://portals.broadinstitute.org/ccle. TCGA data were downloaded from https://portal.gdc.cancer.gov/.

Gene filters

The gene filters were slightly different for cell lines and patient samples, but the essential idea was the same: the genes were first filtered by their transcriptional level, and then by their correlation level, to drug sensitivity. In all the filtering steps, TPM values across all samples were considered to calculate the mean value, Pearson correlation coefficient, or the dispersion scores.

For gene filters applied to cell lines

First, genes with mean expression level log(1 + TPM) > 1 (across all samples) were retained. After this step, about one-third of the genes remained (10,000–20,000 genes, the number was different for different cancer type-drug pairs) (Figure 1B). Second, genes with a Pearson correlation coefficient (between TPM and Z scores of IC_{50}) > 0.25 were retained. After this step, about 3,000 genes were retained. Third, genes were scored and rated by the absolute values of the Fisher's linear discriminant of the gene transcription magnitudes between cell lines with the highest and lowest 15% of the IC_{50} values. Genes were enumerated in the subsequent KNN algorithm by order. KNN algorithm cross-validation determined the top m (m is the number of genes, $m \leq 30$) genes to be selected as digital markers to reach the best performance.

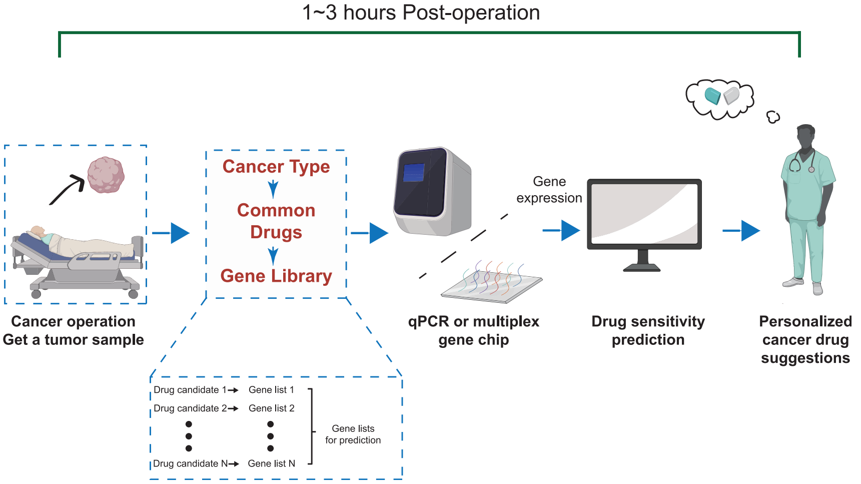

Figure 5. The proposed clinical application pipeline of the digital marker-based rapid drug effectiveness prediction

Figure created with Biorender (https://biorender.com).

For cell lines, we built the model based on the data in both CCLE and GDSC (method 1). We filtered and scored the genes in both datasets separately, and then added the scores of each gene obtained in the two datasets and finally got their scores and ranks. Then we performed 5-fold cross-validation on the ranked genes to compose the digital markers and predicted drug sensitivity values on the two datasets separately. To test the generalization of our model, we performed "cross-prediction" (method 2) for cell lines, i.e., predicting drug sensitivity of one dataset (CCLE/GDSC) using the ranked genes filtered by the other dataset (GDSC/CCLE). Training and testing the model in the same dataset was also performed for comparison (method 3).

For patient samples, we performed 5-fold cross-validation on the ranked genes and obtained the prediction models and digital markers when reaching the best performance. Six cancer type-drug pairs were analyzed (Table S2), and the prediction outcome of COAD, READ, and 5-FU was validated by experiment.

For gene filters applied to patient samples

First, genes with mean expression level log(TPM) > 0 (across all samples) were retained. After this step, about 10,000–20,000 genes were retained (the number was different for different cancer type-drug pairs) (Figure 1C). Second, genes were scored and rated by the p values from the Mann-Whitney U test. According to the drug sensitivity label, patients were divided into two groups for each cancer type-drug pair. Mann-Whitney U test was performed on the TPM of each gene across the two groups of patients. Scored and rated genes were enumerated in the subsequent KNN algorithm with order. KNN algorithm cross-validation determined the top m (m is the number of genes, $m \leq 30$) genes to be selected as digital markers to reach the best performance (Figures 1C and S14).

Gene selection and KNN enumeration

After applying the gene filters, the remaining genes were scored and rated according to their importance for prediction of drug sensitivity. The KNN algorithm was used to finalize the gene list for each drug. The top m (m = 1–30) genes were enumerated in the subsequent KNN 5-fold cross-validation with order. For each m, the parameter k of KNN was also enumerated to reach the best cross-validation performance. Finally, the composition of the digital markers and parameter k_0 were determined with the desirable AUC score in the 5-fold cross-validation process.

Gene selection was performed for each cancer type-drug pairs in most of the situations. For cancer cell lines, to finalize the digital markers, we performed gene filtering on both datasets separately and synthesized the scores of genes as the rating criterion to finalize the gene candidate list. Then we performed KNN cross-validation on CCLE/GDSC datasets according to the gene candidate list to determine the digital markers for a certain cancer type-drug pair. To test the robustness of the digital markers, we also screened digital markers separately on CCLE and GDSC.

The wet laboratory validation adopted a different procedure for gene selection. For experimental validation of prediction of drug efficacy for cell lines, to select the genes with the most stable prediction ability and reduce the impact of errors (inconsistency) in the databases, the screened genes for all 198 drugs in the GDSC dataset were calculated and the number of occurrences of each gene was counted. Genes with more than 20 occurrences were considered to have stable predictive ability in a specified tissue and were preferentially selected in the gene list to compose the digital marker and for qRT-PCR quantification. The genes were screened on the data from GDSC and CCLE separately, and only genes screened from both databases were further analyzed.

Prediction of drug sensitivity

We performed the gene filtering process to screen gene candidates (which will feed into the KNN model to generate the final digital markers) for each cancer type-drug pair, which means that, for each cancer type-drug pair, we established a set of digital markers. In total, we studied three cancer types and eight drugs for cancer cell lines, and six cancer type-drug pairs for patient samples, listed in Table S2.

Graphic user interface for prediction of drug sensitivity of cancer cell lines

The Tkinter package in python was used to set up the graphic user interface (GUI) to show the possible process of prediction of drug sensitivity as well as drug recommendation. The codes can be downloaded in GitHub. By entering "python GUI.py" in the command line window, you can run the GUI program. In the GUI, first, a file containing digital marker genes and gene transcription levels (in TPM form) is required. After uploading the file and selecting cancer type, the prediction result is shown by clicking the "Predict" button. The IC_{50} values and Z scores of the IC_{50} values of the four drugs are shown. Relative Z scores of IC_{50} are computed by normalizing the IC_{50} values among the cell lines of the certain cancer type, and are considered to determine whether the drug is effective or not. Drugs with relative Z scores below -1 are considered "sensitive" and shown in green, and those with relative Z scores above 1 are considered "non-sensitive" and shown in red, and others shown in black represent "neutral" outcomes.

Experimental evaluation of drug sensitivity

For evaluation of the drug sensitivity of cancer cell lines, cells were seeded in 96-well plates at a density of 4,000 cells per well. Drugs (cisplatin, paclitaxel, and 5-FU, purchased from MCE) of seven different concentrations (Figure S10) were tested. The drugs were first solubilized in DMSO. Each dosing condition was tested with four replicates (i.e., four wells containing 2D cultured cells). After 72 h treatment, a cell viability assay, using the Cell Counting Kit 8 (HY-K0301, MCE), was conducted according to the manufacturer's instructions.

For evaluation of the drug sensitivity of tumor organoids, we adopted the protocol from Jiang et al.[46] Tumor organoids were cultured in the 96-well plates (two organoids per well). 5-FU of seven different concentrations (0, 1, 10, 50, 100, 200, 500 μM in DMSO) were tested (Figure S11). Each dosing condition was tested with five replicates (i.e., five organoid-containing wells). After 72 h treatment, a CellTiter-Glo 3D cell viability assay (Promega, USA) was employed to detect cell viability according to the manufacturer's instructions. The cell viability rates were calculated. The drug response curves were fitted by GraphPad, and IC_{50} values were calculated.

RNA-seq and cDNA preparation

RNA-seq data for cell lines were acquired using GENEWIZ (Suzhou, China). The cell lines in culture were sent to GENEWIZ to perform standard RNA-seq

analysis. Total RNA of both cell lines and tumor organoids were extracted using the Eastep Super Total RNA Extraction Kit (Promega)/RNeasy Mini Kit (-QIAGEN, Germany), following the manufacturer's instructions. The purity and concentration of RNA were determined by using a NanoDrop 2000 spectrophotometer (Thermo Fisher Scientific). RNA (1–2 μg) were used to prepare cDNA using the PrimeScript RT Reagent Kit (TaKaRa, Japan) with random primers and oligo dT primers.

qRT-PCR assay

qRT-PCR were performed in 96-well plates (Sangon, China) on a Real-Time PCR Detection system (Bio-Rad, USA). Primer sequences and characteristics are described in Tables S8 and S9. The qRT-PCR reaction mix was composed of 12.5 μL GoTaq Green Master Mix, 2X (Promega), 10 μM upstream primer, 10 μM downstream primer, 2 μL DNA template, and 25 μL nuclease-free water. Cycling reaction conditions were 95°C for 10 min, 40 cycles of 95°C for 15 s, and 60°C for 1 min. All qRT-PCR experiments were performed with three biological replicates. All qRT-PCR data are shown in the Figures S15 and S16.

SUPPLEMENTAL INFORMATION

Supplemental information can be found online at https://doi.org/10.1016/j.patter.2021.100360.

ACKNOWLEDGMENTS

The work was supported by the National Natural Science Foundation of China (grant no. 61971255), the Natural Science Foundation of Guangdong Province (grant no. 2021B1515020092), the Shenzhen Science and Technology Innovation Commission (grant nos. RCYX20200714114736146 and WDZC20200821141349001), and the Shenzhen Bay Laboratory Fund (grant no. SZBL2020090501014).

AUTHOR CONTRIBUTIONS

J.F. and Y.F. contributed equally to this study. J.F. contributed to the algorithms and codes, drug screening, and manuscript drafting. Y.F. contributed to tumor organoid sampling and gene transcription quantification. Y.C. provided help on figure editing and paper revision. Z.W. and H.Z. provided guidance on biological experiments. E.A.G. provided help on text editing. Q.L., S.C., and W.Z. provided patient samples. S.M. conceived and advised on this work. All authors edited and approved the final version of the manuscript.

DECLARATION OF INTERESTS

The authors declare no competing interests.

INCLUSION AND DIVERSITY

We worked to ensure gender balance in the recruitment of human subjects.

While citing references scientifically relevant for this work, we also actively worked to promote gender balance in our reference list.

The author list of this paper includes contributors from the location where the research was conducted who participated in the data collection, design, analysis, and/or interpretation of the work.

Received: May 24, 2021
Revised: June 29, 2021
Accepted: September 8, 2021
Published: September 29, 2021

REFERENCES

1. Lambert, A.W., Pattabiraman, D.R., and Weinberg, R.A. (2017). Emerging biological principles of metastasis. Cell *168*, 670–691. https://doi.org/10.1016/j.cell.2016.11.037.
2. Dagogo-Jack, I., and Shaw, A.T. (2018). Tumour heterogeneity and resistance to cancer therapies. Nat. Rev. Clin. Oncol. *15*, 81–94. https://doi.org/10.1038/nrclinonc.2017.166.
3. Reiter, J.G., Baretti, M., Gerold, J.M., Makohon-Moore, A.P., Daud, A., Iacobuzio-Donahue, C.A., Azad, N.S., Kinzler, K.W., Nowak, M.A., and Vogelstein, B. (2019). An analysis of genetic heterogeneity in untreated cancers. Nat. Rev. Cancer *19*, 639–650. https://doi.org/10.1038/s41568-019-0185-x.
4. Keller, L., and Pantel, K. (2019). Unravelling tumour heterogeneity by single-cell profiling of circulating tumour cells. Nat. Rev. Cancer *19*, 553–567. https://doi.org/10.1038/s41568-019-0180-2.
5. Paull, E.O., Aytes, A., Jones, S.J., Subramaniam, P.S., Giorgi, F.M., Douglass, E.F., Tagore, S., Chu, B., Vasciaveo, A., and Zheng, S. (2021). A modular master regulator landscape controls cancer transcriptional identity. Cell *184*, 334–351.e20. https://doi.org/10.1016/j.cell.2020.11.045.
6. Chen, X., Zhou, J., Zhang, R., Wong, A.K., Park, C.Y., Theesfeld, C.L., and Troyanskaya, O.G. (2021). Tissue-specific enhancer functional networks for associating distal regulatory regions to disease. Cell Syst. *12*, 353–362.e56. https://doi.org/10.1016/j.cels.2021.02.002.
7. Garnett, M.J., Edelman, E.J., Heidorn, S.J., Greenman, C.D., Dastur, A., Lau, K.W., Greninger, P., Thompson, I.R., Luo, X., and Soares, J. (2012). Systematic identification of genomic markers of drug sensitivity in cancer cells. Nature *483*, 570–575. https://doi.org/10.1038/nature11005.
8. Yang, W., Soares, J., Greninger, P., Edelman, E.J., Lightfoot, H., Forbes, S., Bindal, N., Beare, D., Smith, J.A., and Thompson, I.R. (2012). Genomics of Drug Sensitivity in Cancer (GDSC): a resource for therapeutic biomarker discovery in cancer cells. Nucleic Acids Res. *41*, D955–D961. https://doi.org/10.1093/nar/gks1111.
9. Iorio, F., Knijnenburg, T.A., Vis, D.J., Bignell, G.R., Menden, M.P., Schubert, M., Aben, N., Gonçalves, E., Barthorpe, S., and Lightfoot, H. (2016). A landscape of pharmacogenomic interactions in cancer. Cell *166*, 740–754. https://doi.org/10.1016/j.cell.2016.06.017.
10. Barretina, J., Caponigro, G., Stransky, N., Venkatesan, K., Margolin, A.A., Kim, S., Wilson, C.J., Lehár, J., Kryukov, G.V., and Sonkin, D. (2012). The Cancer Cell Line Encyclopedia enables predictive modelling of anticancer drug sensitivity. Nature *483*, 603–607. https://doi.org/10.1038/nature11003.
11. Ghandi, M., Huang, F.W., Jané-Valbuena, J., Kryukov, G.V., Lo, C.C., McDonald, E.R., Barretina, J., Gelfand, E.T., Bielski, C.M., and Li, H. (2019). Next-generation characterization of the Cancer Cell Line Encyclopedia. Nature *569*, 503–508. https://doi.org/10.1038/s41586-019-1186-3.
12. Liu, J., Lichtenberg, T., Hoadley, K.A., Poisson, L.M., Lazar, A.J., Cherniack, A.D., Kovatich, A.J., Benz, C.C., Levine, D.A., and Lee, A.V. (2018). An integrated TCGA pan-cancer clinical data resource to drive high-quality survival outcome analytics. Cell *173*, 400–416.e11. https://doi.org/10.1016/j.cell.2018.02.052.
13. Papillon-Cavanagh, S., De Jay, N., Hachem, N., Olsen, C., Bontempi, G., Aerts, H.J., Quackenbush, J., and Haibe-Kains, B. (2013). Comparison and validation of genomic predictors for anticancer drug sensitivity. J. Am. Med. Inform. Assoc. *20*, 597–602. https://doi.org/10.1136/amiajnl-2012-001442.
14. Luo, Y., Zhao, X., Zhou, J., Yang, J., Zhang, Y., Kuang, W., Peng, J., Chen, L., and Zeng, J. (2017). A network integration approach for drug-target interaction prediction and computational drug repositioning from heterogeneous information. Nat. Commun. *8*, 1–13. https://doi.org/10.1038/s41467-017-00680-8.
15. Kong, J., Lee, H., Kim, D., Han, S.K., Ha, D., Shin, K., and Kim, S. (2020). Network-based machine learning in colorectal and bladder organoid models predicts anti-cancer drug efficacy in patients. Nat. Commun. *11*, 1–13. https://doi.org/10.1038/s41467-020-19313-8.
16. Chiu, Y.-C., Chen, H.-I.H., Zhang, T., Zhang, S., Gorthi, A., Wang, L.-J., Huang, Y., and Chen, Y. (2019). Predicting drug response of tumors from integrated genomic profiles by deep neural networks. BMC Med. Genomics *12*, 143–155. https://doi.org/10.1186/s12920-018-0460-9.
17. Sakellaropoulos, T., Vougas, K., Narang, S., Koinis, F., Kotsinas, A., Polyzos, A., Moss, T.J., Piha-Paul, S., Zhou, H., and Kardala, E. (2019).

CellPress
OPEN ACCESS

A deep learning framework for predicting response to therapy in cancer. Cell Rep. *29*, 3367–3373.e4. https://doi.org/10.1016/j.celrep.2019.11.017.

18. Sanchez-Vega, F., Mina, M., Armenia, J., Chatila, W.K., Luna, A., La, K.C., Dimitriadoy, S., Liu, D.L., Kantheti, H.S., and Saghafinia, S. (2018). Oncogenic signaling pathways in The Cancer Genome Atlas. Cell *173*, 321–337.e10. https://doi.org/10.1016/j.cell.2018.03.035.
19. Lee, M.-C.W., Lopez-Diaz, F.J., Khan, S.Y., Tariq, M.A., Dayn, Y., Vaske, C.J., Radenbaugh, A.J., Kim, H.J., Emerson, B.M., and Pourmand, N. (2014). Single-cell analyses of transcriptional heterogeneity during drug tolerance transition in cancer cells by RNA sequencing. Proc. Natl. Acad. Sci. U S A *111*, E4726–E4735. https://doi.org/10.1073/pnas.1404656111.
20. Zimmer, A., Katzir, I., Dekel, E., Mayo, A.E., and Alon, U. (2016). Prediction of multidimensional drug dose responses based on measurements of drug pairs. Proc. Natl. Acad. Sci. U S A *113*, 10442–10447. https://doi.org/10.1073/pnas.1606301113.
21. Trédan, O., Galmarini, C.M., Patel, K., and Tannock, I.F. (2007). Drug resistance and the solid tumor microenvironment. J. Natl. Cancer Inst. *99*, 1441–1454. https://doi.org/10.1093/jnci/djm135.
22. Hirata, E., and Sahai, E. (2017). Tumor microenvironment and differential responses to therapy. Cold Spring Harbor Perspect. Med. *7*, a026781. https://doi.org/10.1101/cshperspect.a026781.
23. Stark, R., Grzelak, M., and Hadfield, J. (2019). RNA sequencing: the teenage years. Nat. Rev. Genet. *20*, 631–656. https://doi.org/10.1038/s41576-019-0150-2.
24. Schurch, N.J., Schofield, P., Gierliński, M., Cole, C., Sherstnev, A., Singh, V., Wrobel, N., Gharbi, K., Simpson, G.G., and Owen-Hughes, T. (2016). How many biological replicates are needed in an RNA-seq experiment and which differential expression tool should you use? RNA *22*, 839–851. https://doi.org/10.1261/rna.053959.115.
25. Alexander, M., Blum, R., Burbury, K., Coutsouvelis, J., Dooley, M., Fazil, O., Griffiths, T., Ismail, H., Joshi, S., and Love, N. (2017). Timely initiation of chemotherapy: a systematic literature review of six priority cancers–results and recommendations for clinical practice. Intern. Med. J. *47*, 16–34. https://doi.org/10.1111/imj.13190.
26. Fu, M., Rao, R., Sudhakar, D., Hogue, C.P., Rutta, Z., Morales, S., Gordon, L.K., Braun, J., Goodglick, L., and Wadehra, M. (2011). Epithelial membrane protein-2 promotes endometrial tumor formation through activation of FAK and Src. PLoS One *6*, e19945. https://doi.org/10.1371/journal.pone.0019945.
27. Kiyohara, M.H., Dillard, C., Tsui, J., Kim, S.R., Lu, J., Sachdev, D., Goodglick, L., Tong, M., Torous, V.F., and Aryasomayajula, C. (2017). EMP2 is a novel therapeutic target for endometrial cancer stem cells. Oncogene *36*, 5793–5807. https://doi.org/10.1038/onc.2017.142.
28. Jacobs, A.T., and Marnett, L.J. (2009). HSF1-mediated BAG3 expression attenuates apoptosis in 4-hydroxynonenal-treated colon cancer cells via stabilization of anti-apoptotic Bcl-2 proteins. J. Biol. Chem. *284*, 9176–9183. https://doi.org/10.1074/jbc.M808656200.
29. Li, N., Chen, M., Cao, Y., Li, H., Zhao, J., Zhai, Z., Ren, F., and Li, K. (2018). Bcl-2-associated athanogene 3 (BAG3) is associated with tumor cell proliferation, migration, invasion and chemoresistance in colorectal cancer. BMC Cancer *18*, 1–15. https://doi.org/10.1186/s12885-018-4657-2.
30. Sondka, Z., Bamford, S., Cole, C.G., Ward, S.A., Dunham, I., and Forbes, S.A. (2018). The COSMIC Cancer Gene Census: describing genetic dysfunction across all human cancers. Nat. Rev. Cancer *18*, 696–705. https://doi.org/10.1038/s41568-018-0060-1.
31. O'connell, J., O'sullivan, G.C., Collins, J.K., and Shanahan, F. (1996). The Fas counterattack: Fas-mediated T cell killing by colon cancer cells expressing Fas ligand. J. Exp. Med. *184*, 1075–1082. https://doi.org/10.1084/jem.184.3.1075.
32. Pitti, R.M., Marsters, S.A., Lawrence, D.A., Roy, M., Kischkel, F.C., Dowd, P., Huang, A., Donahue, C.J., Sherwood, S.W., and Baldwin, D.T. (1998). Genomic amplification of a decoy receptor for Fas ligand in lung and colon cancer. Nature *396*, 699–703. https://doi.org/10.1038/25387.
33. O'CONNELL, J., Bennett, M.W., Nally, K., Houston, A., O'SULLIVAN, G.C., and Shanahan, F. (2000). Altered mechanisms of apoptosis in colon cancer: Fas resistance and counterattack in the tumor-immune conflict. Ann. New York Acad. Sci. *910*, 178–195. https://doi.org/10.1111/j.1749-6632.2000.tb06708.x.
34. Gao, J., Morgan, W.A., Sanchez-Medina, A., and Corcoran, O. (2011). The ethanol extract of *Scutellaria baicalensis* and the active compounds induce cell cycle arrest and apoptosis including upregulation of p53 and Bax in human lung cancer cells. Toxicol. Appl. Pharmacol. *254*, 221–228. https://doi.org/10.1016/j.taap.2011.03.016.
35. Brambilla, E., Gazzeri, S., Lantuejoul, S., Coll, J.L., Moro, D., Negoescu, A., and Brambilla, C. (1998). p53 mutant immunophenotype and deregulation of p53 transcription pathway (Bcl2, Bax, and Waf1) in precursor bronchial lesions of lung cancer. Clin. Cancer Res. *4*, 1609–1618.
36. Perego, P., Giarola, M., Righetti, S.C., Supino, R., Caserini, C., Delia, D., Pierotti, M.A., Miyashita, T., Reed, J.C., and Zunino, F. (1996). Association between cisplatin resistance and mutation of p53 gene and reduced Bax expression in ovarian carcinoma cell systems. Cancer Res. *56*, 556–562.
37. Tajeddine, N., Galluzzi, L., Kepp, O., Hangen, E., Morselli, E., Senovilla, L., Araujo, N., Pinna, G., Larochette, N., and Zamzami, N. (2008). Hierarchical involvement of Bak, VDAC1 and Bax in cisplatin-induced cell death. Oncogene *27*, 4221–4232. https://doi.org/10.1038/onc.2008.63.
38. Prest, S.J., May, F.E., and Westley, B.R. (2002). The estrogen-regulated protein, TFF1, stimulates migration of human breast cancer cells. FASEB J. *16*, 592–594. https://doi.org/10.1096/fj.01-0498fje.
39. Buache, E., Etique, N., Alpy, F., Stoll, I., Muckensturm, M., Reina-San-Martin, B., Chenard, M., Tomasetto, C., and Rio, M. (2011). Deficiency in trefoil factor 1 (TFF1) increases tumorigenicity of human breast cancer cells and mammary tumor development in TFF1-knockout mice. Oncogene *30*, 3261–3273. https://doi.org/10.1038/onc.2011.41.
40. May, F.E., and Westley, B.R. (2015). TFF3 is a valuable predictive biomarker of endocrine response in metastatic breast cancer. Endocrine Related Cancer *22*, 465. https://doi.org/10.1530/ERC-15-0129.
41. Ahmed, A.R., Griffiths, A.B., Tilby, M.T., Westley, B.R., and May, F.E. (2012). TFF3 is a normal breast epithelial protein and is associated with differentiated phenotype in early breast cancer but predisposes to invasion and metastasis in advanced disease. Am. J. Pathol. *180*, 904–916. https://doi.org/10.1016/j.ajpath.2011.11.022.
42. Deka, J., Wiedemann, N., Anderle, P., Murphy-Seiler, F., Bultinck, J., Eyckerman, S., Stehle, J.-C., André, S., Vilain, N., and Zilian, O. (2010). Bcl9/Bcl9l are critical for Wnt-mediated regulation of stem cell traits in colon epithelium and adenocarcinomas. Cancer Res. *70*, 6619–6628. https://doi.org/10.1158/0008-5472.CAN-10-0148.
43. Arya, M., Shergill, I.S., Williamson, M., Gommersall, L., Arya, N., and Patel, H.R. (2005). Basic principles of real-time quantitative PCR. Expert Rev. Mol. Diagn. *5*, 209–219. https://doi.org/10.1586/14737159.5.2.209.
44. Everaert, C., Luypaert, M., Maag, J.L., Cheng, Q.X., Dinger, M.E., Hellemans, J., and Mestdagh, P. (2017). Benchmarking of RNA-sequencing analysis workflows using whole-transcriptome RT-qPCR expression data. Sci. Rep. *7*, 1–11. https://doi.org/10.1038/s41598-017-01617-3.
45. Vlachogiannis, G., Hedayat, S., Vatsiou, A., Jamin, Y., Fernández-Mateos, J., Khan, K., Lampis, A., Eason, K., Huntingford, I., and Burke, R. (2018). Patient-derived organoids model treatment response of metastatic gastrointestinal cancers. Science *359*, 920–926. https://doi.org/10.1126/science.aao2774.
46. Jiang, S., Zhao, H., Zhang, W., Wang, J., Liu, Y., Cao, Y., Zheng, H., Hu, Z., Wang, S., and Zhu, Y. (2020). An automated organoid platform with inter-organoid homogeneity and inter-patient heterogeneity. Cell Rep. Med. *1*, 100161. https://doi.org/10.1016/j.xcrm.2020.100161.

Patterns, Volume *2*

Supplemental information

Multiplex gene quantification as digital markers for extremely rapid evaluation of chemo-drug sensitivity

Jiaqi Fan, Yilin Feng, Yifan Cheng, Zitian Wang, Haoran Zhao, Edgar A. Galan, Quanxing Liao, Shuzhong Cui, Weijie Zhang, and Shaohua Ma

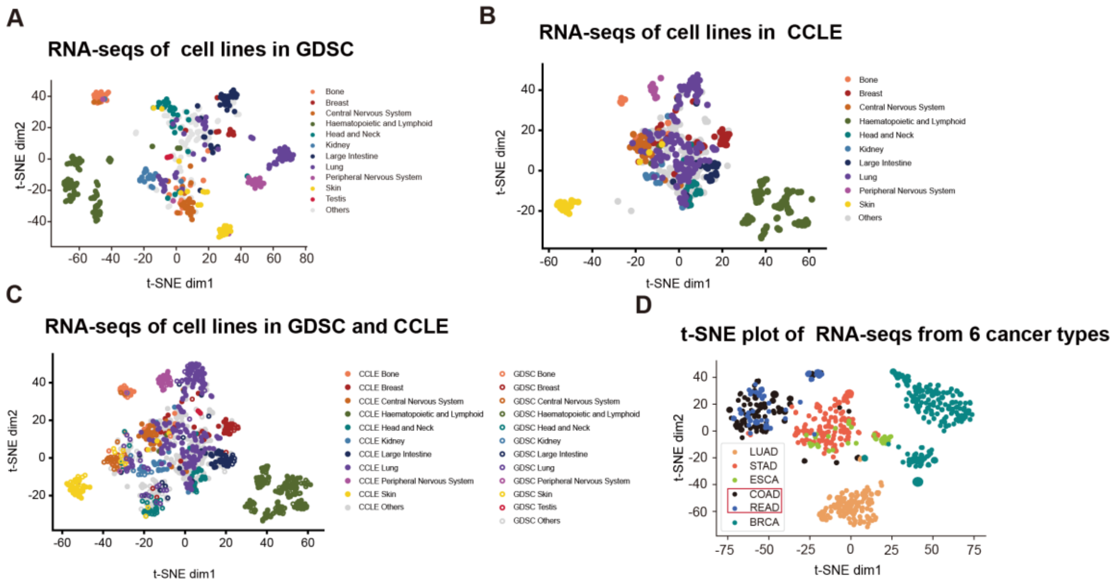

Figure S1. Two-dimensional t-SNE plot of the RNA-seq data of cancer cell lines in CCLE and GDSC, and patient samples in TCGA. (A, B) Two-dimensional t-SNE plot of the RNA-seq data of cancer cell lines in GDSC (A) and CCLE (B). Colors denote cancer types. Each dot represents a cell line. (C) Two-dimensional t-SNE plot of the RNA-seq data of cancer cell lines in CCLE and GDSC. Colors denote cancer types. Each dot represents a cell line. The filled circles denote cell lines from CCLE, and the hollow circles denote cell lines from GDSC. (D) Two-dimensional t-SNE plot of the RNA-seq data of 6 types of tumors. Each color represents a tumor type. Each dot represents a tumor sample.

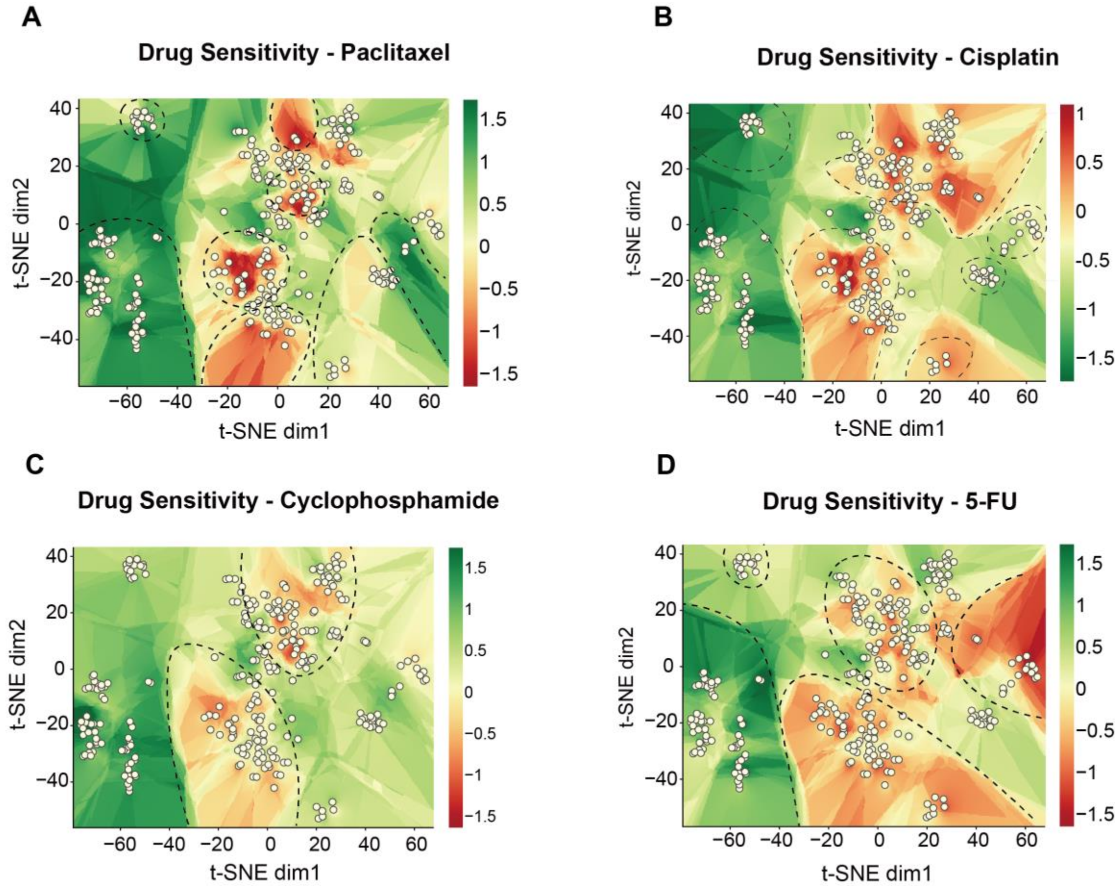

Figure S2. Cancer type-dependent drug sensitivity distribution. Drug sensitivity distribution toward Paclitaxel(A), Cisplatin(B), Cyclophosphamide(C) and 5-FU(D). The dots represent cancer cell lines mapped by t-SNE as in **Supplementary Fig. 1A**. Colors indicate the sensitivity magnitude (z-score of IC50; green, sensitive; red, non-sensitive) predicted by KNN regression in the two-dimensional area. For KNN regression, k=7.

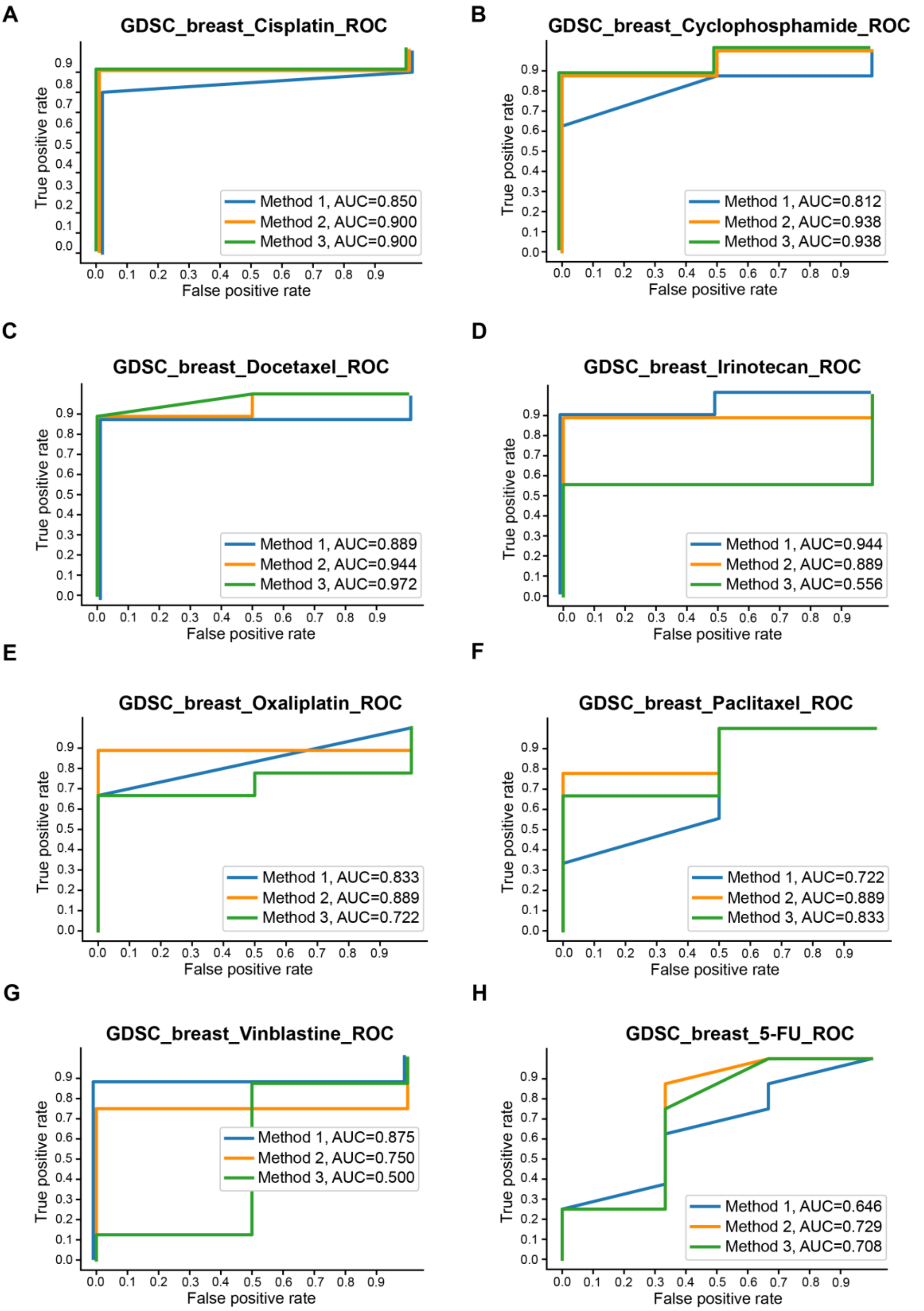

Figure S3. ROC curves for cross-validation of drug sensitivity prediction on breast cancer cell lines in GDSC. The three curves in each plot refer to filtering and scoring genes on both datasets (Method 1), on single but one same dataset (Method 2), and on single but a different dataset (Method 3). IC50 z-score threshold = -1.0. Results for breast cancer and Cisplatin (A), Cyclophosphamide (B), Docetaxel (C), Irinotecan (D), Oxaliplatin (E), Paclitaxel (F), Vinblastine (G) and 5-FU (H) are shown.

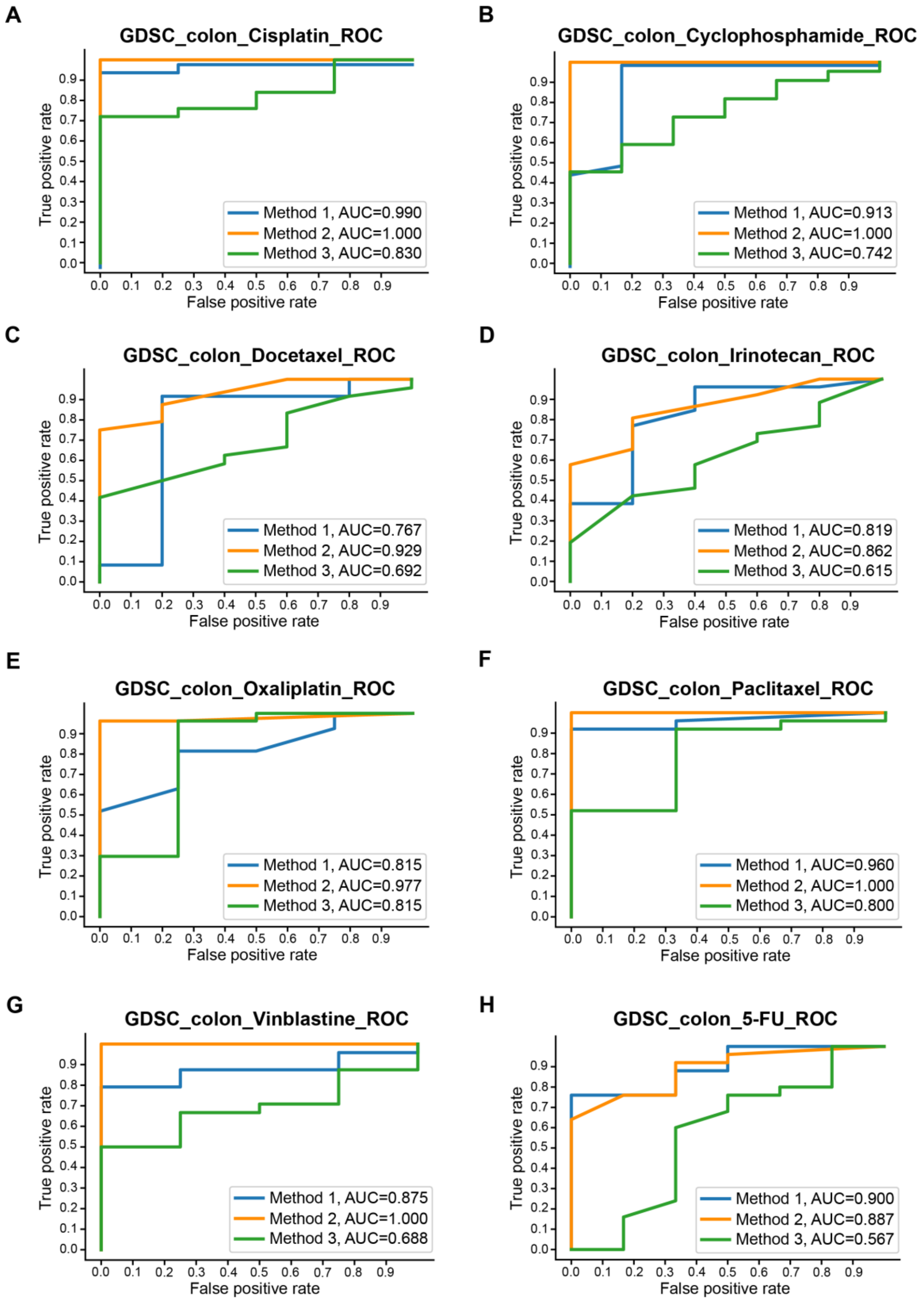

Figure S4. ROC curves for cross-validation of drug sensitivity prediction on colorectal cancer cell lines in GDSC. The three curves in each plot refer to filtering and scoring genes on both datasets (Method 1), on single but one same dataset (Method 2), and on single but a different dataset (Method 3). IC50 z-score threshold = -1.0. Results for colorectal cancer and Cisplatin (A), Cyclophosphamide (B), Docetaxel (C), Irinotecan (D), Oxaliplatin (E), Paclitaxel (F), Vinblastine (G) and 5-FU (H) are shown.

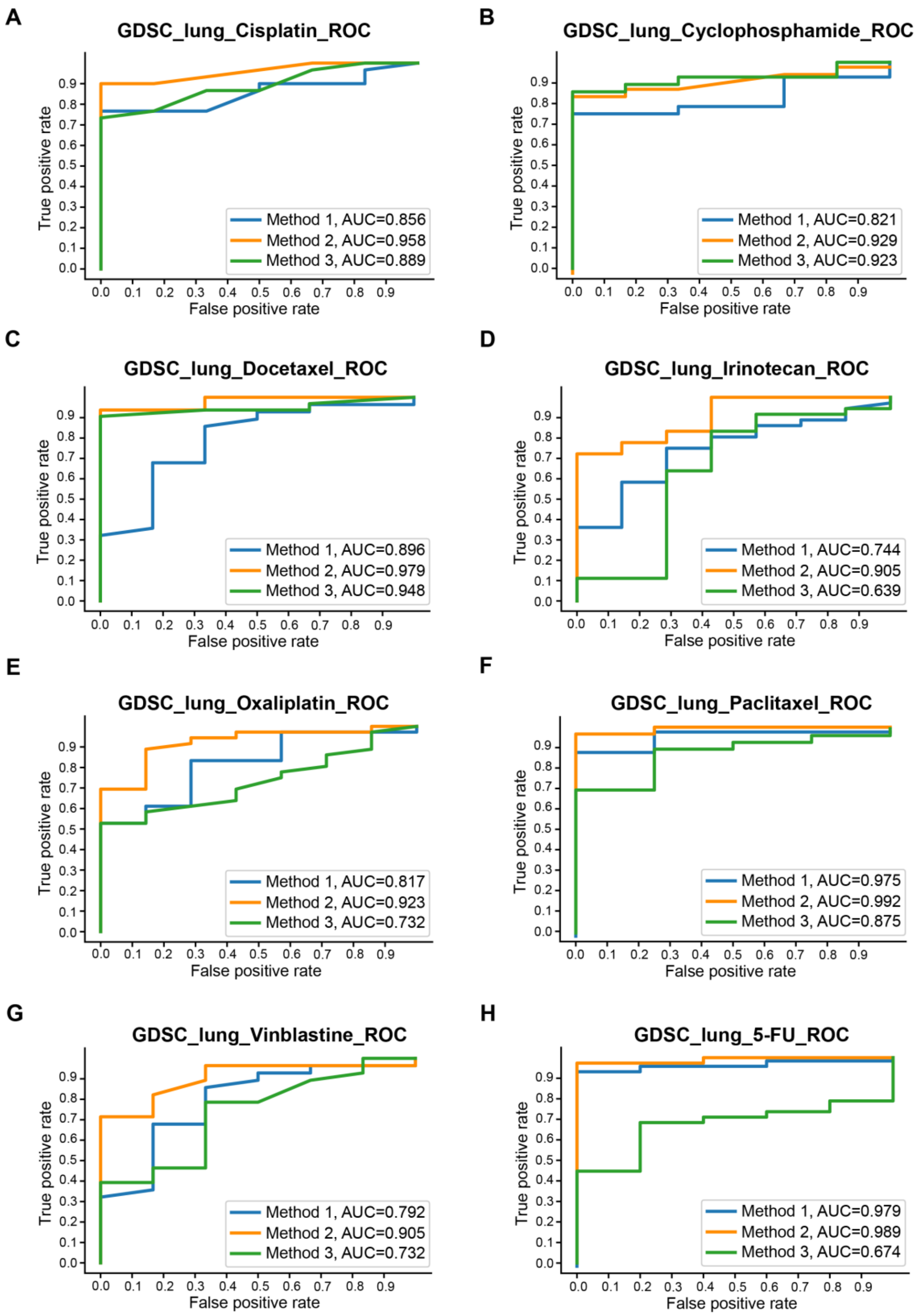

Figure S5. ROC curves for cross-validation of drug sensitivity prediction on lung cancer cell lines in GDSC. The three curves in each plot refer to filtering and scoring genes on both datasets (Method 1), on single but one same dataset (Method 2), and on single but a different dataset (Method 3). IC50 z-score threshold = -1.0. Results for lung cancer and Cisplatin (A), Cyclophosphamide (B), Docetaxel (C), Irinotecan (D), Oxaliplatin (E), Paclitaxel (F), Vinblastine (G) and 5-FU (H) are shown.

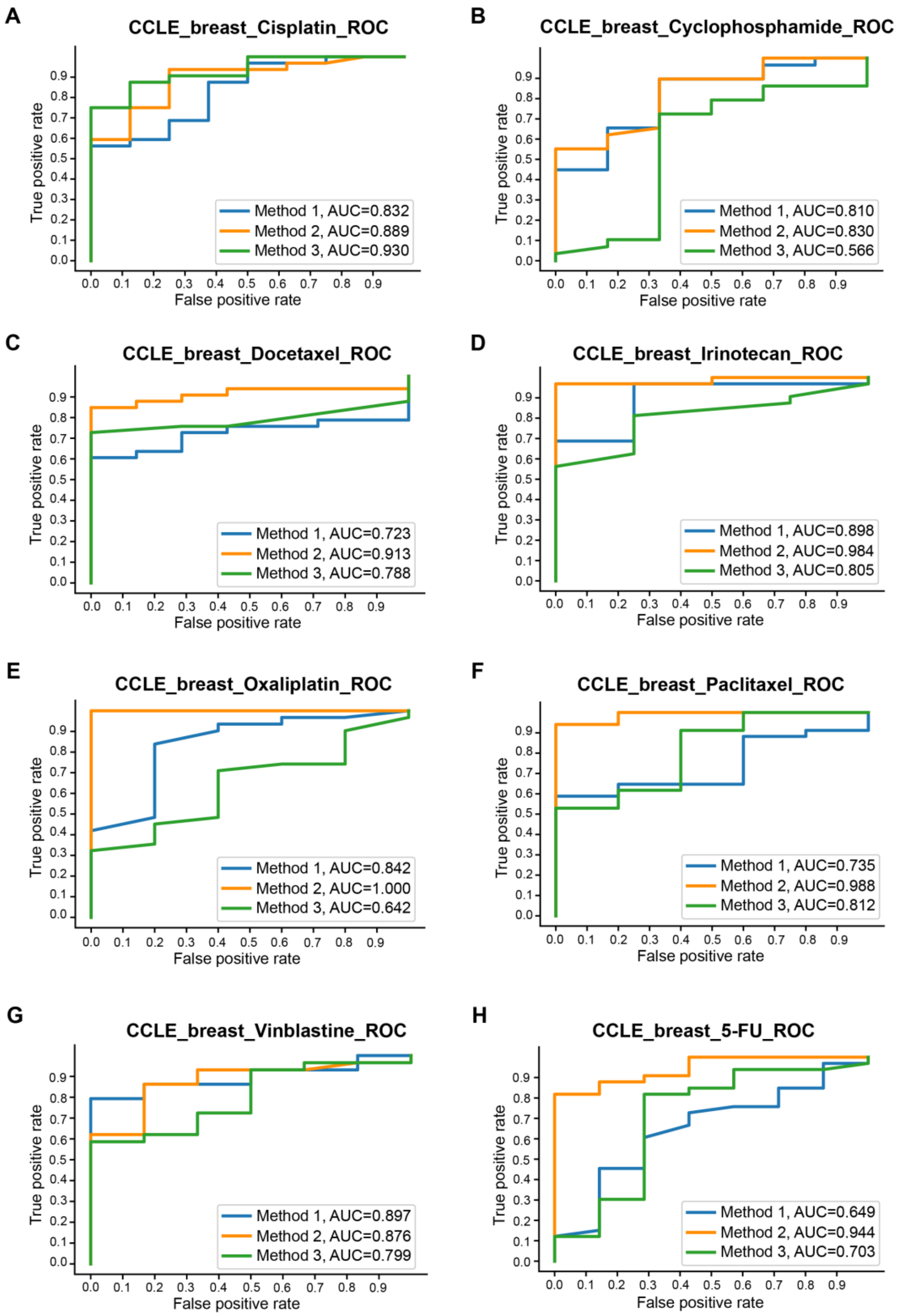

Figure S6. ROC curves for cross-validation of drug sensitivity prediction on breast cancer cell lines in CCLE. The three curves in each plot refer to filtering and scoring genes on both datasets (Method 1), on single but one same dataset (Method 2), and on single but a different dataset (Method 3). IC50 z-score threshold = -1.0. Results for breast cancer and Cisplatin (A), Cyclophosphamide (B), Docetaxel (C), Irinotecan (D), Oxaliplatin (E), Paclitaxel (F), Vinblastine (G) and 5-FU (H) are shown.

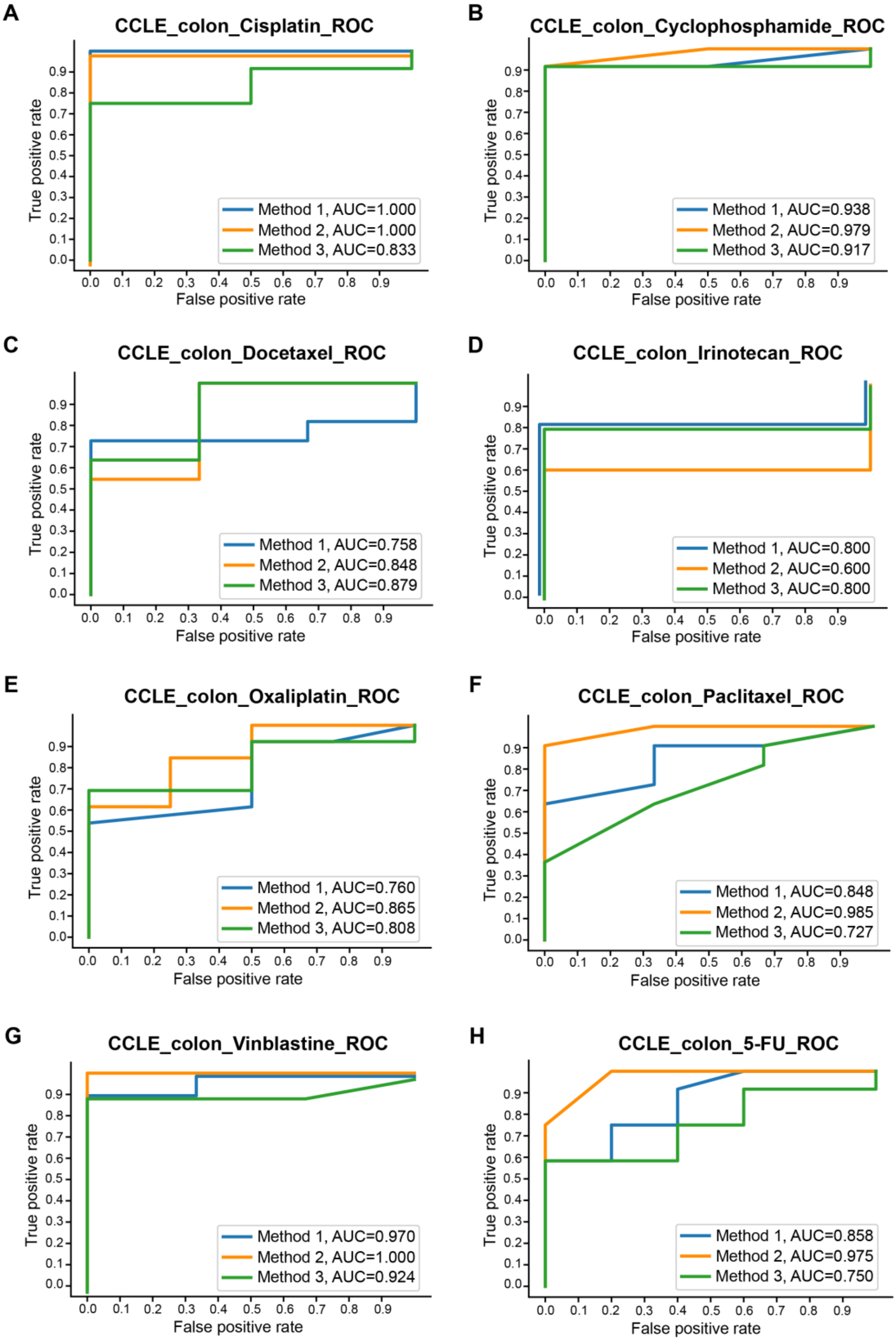

Figure S7. ROC curves for cross-validation of drug sensitivity prediction on colorectal cancer cell lines in CCLE. The three curves in each plot refer to filtering and scoring genes on both datasets (Method 1), on single but one same dataset (Method 2), and on single but a different dataset (Method 3). IC50 z-score threshold = -1.0. Results for colorectal cancer and Cisplatin (A), Cyclophosphamide (B), Docetaxel (C), Irinotecan (D), Oxaliplatin (E), Paclitaxel (F), Vinblastine (G) and 5-FU (H) are shown.

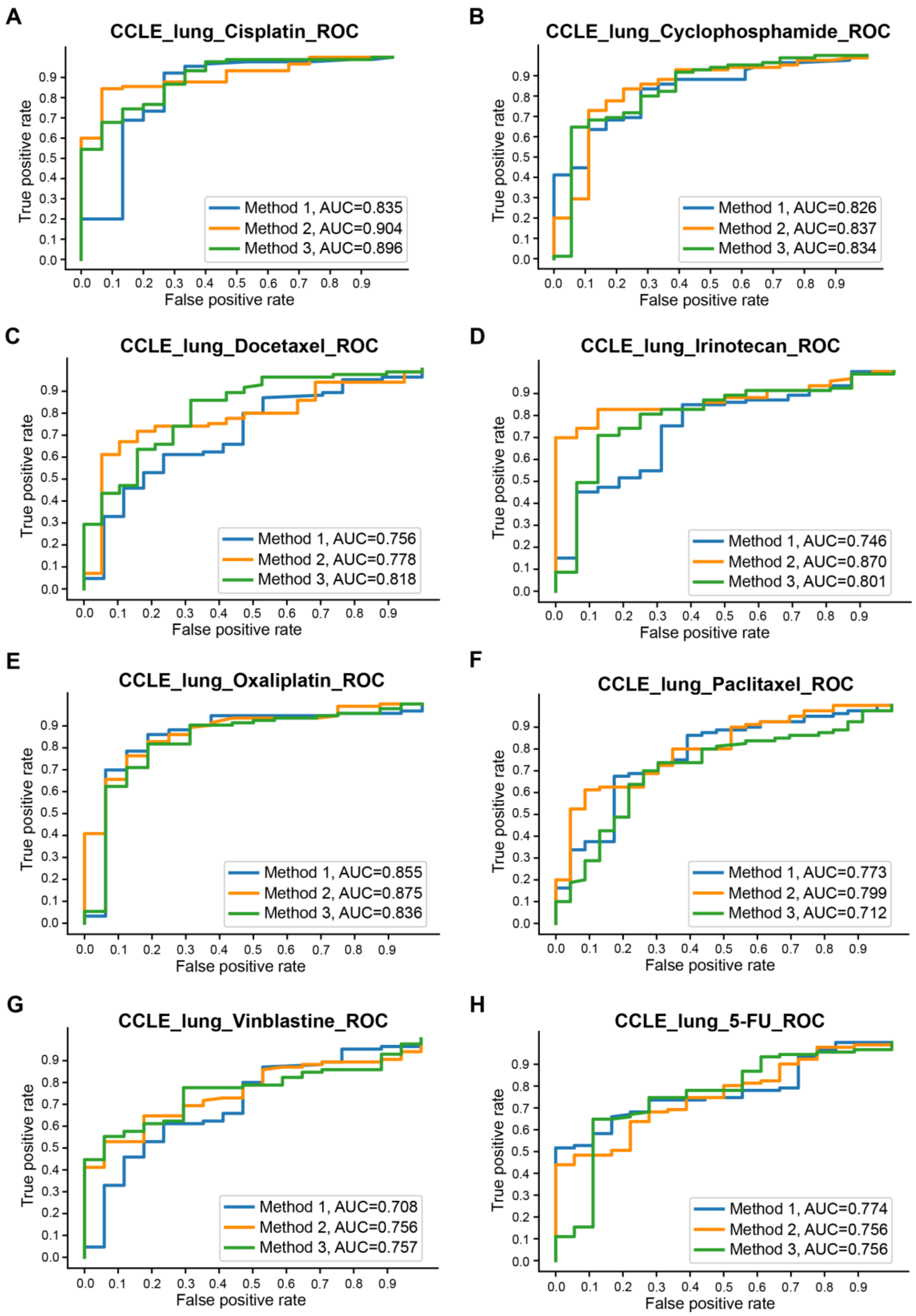

Figure S8. ROC curves for cross-validation of drug sensitivity prediction on lung cancer cell lines in CCLE. The three curves in each plot refer to filtering and scoring genes on both datasets (Method 1), on single but one same dataset (Method 2), and on single but a different dataset (Method 3). IC50 z-score threshold = -1.0. Results for lung cancer and Cisplatin (A), Cyclophosphamide (B), Docetaxel (C), Irinotecan (D), Oxaliplatin (E), Paclitaxel (F), Vinblastine (G) and 5-FU (H) are shown.

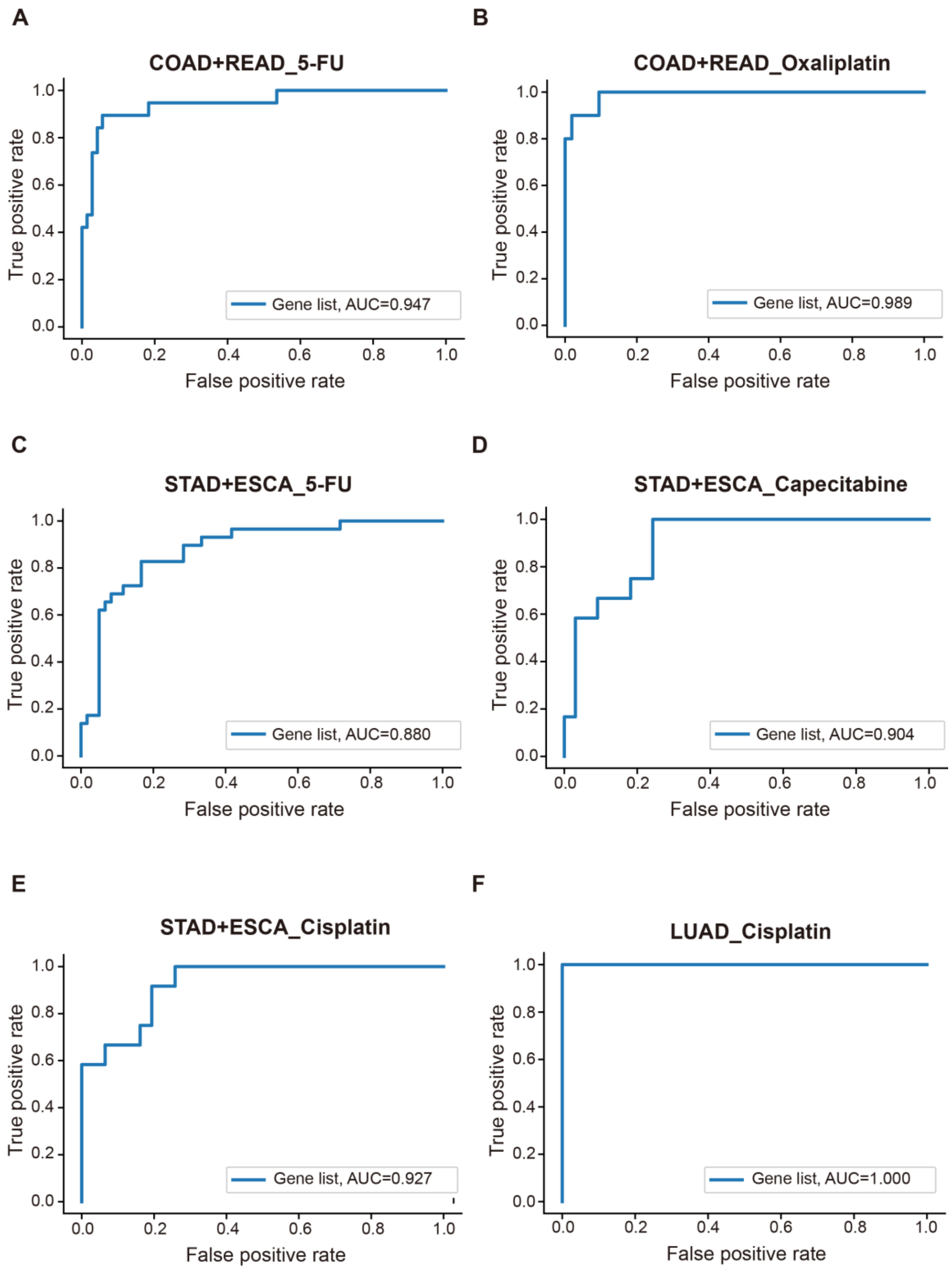

Figure S9. ROC curves for cross-validation of drug sensitivity prediction on 6 cancer type-drug pairs in TCGA. (A) COAD, READ and 5-FU. (B) COAD, READ and Oxaliplatin. (C) STAD, ESCA and 5-FU. (D) STAD, ESCA and Capecitabine. (E) STAD, ESCA and Cisplatin. (F) LUAD and Cisplatin.

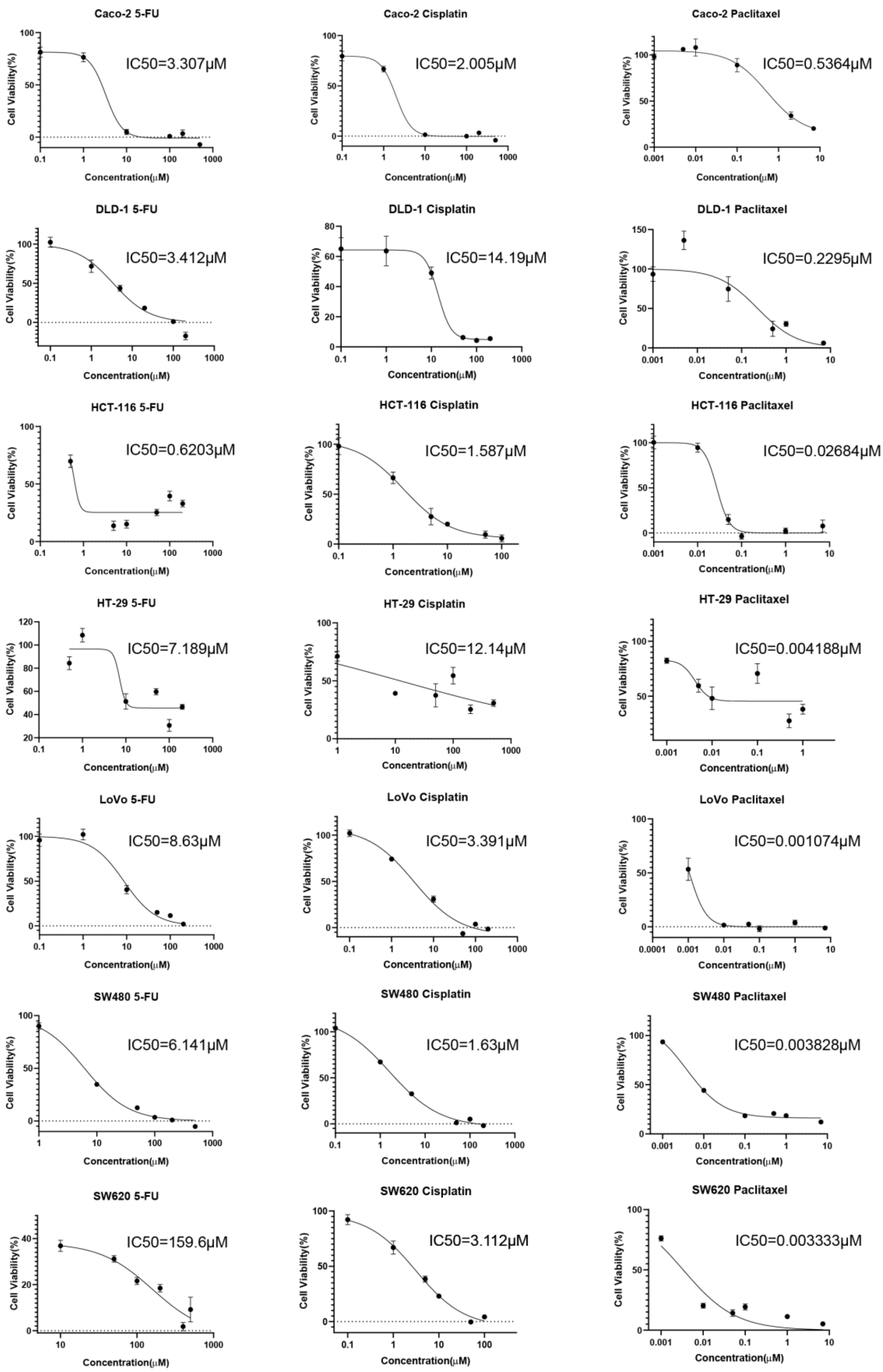

Figure S10. Drug response curves of the cancer cell lines towards 5-FU, Cisplatin and Paclitaxel. The drug response curves and IC50 values of the experimentally tested 7 colorectal cancer cell lines are shown, towards 5-FU, Cisplatin and Paclitaxel.

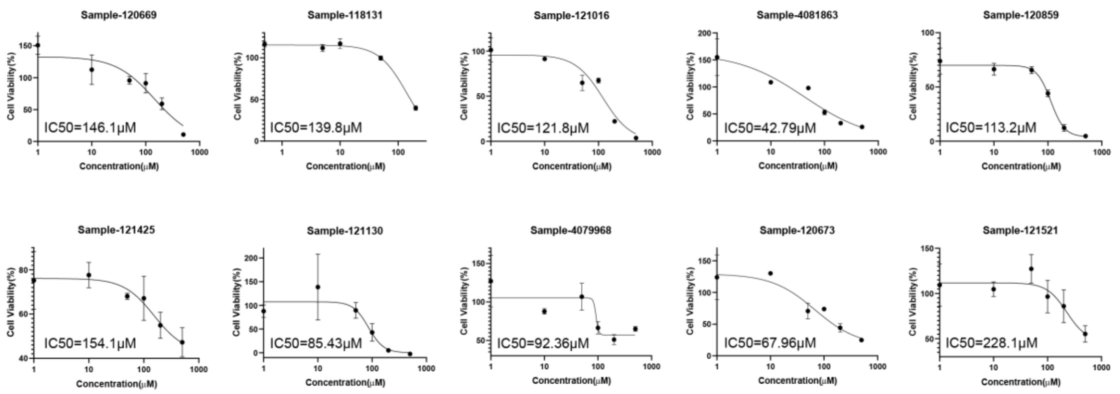

Figure S11. Drug response curves of patient tumor derived organoids towards 5-FU. The drug response curves and IC50 values towards 5-FU of the experimentally tested 10 patient tumor derived organoids, representing responses of their parental tumors, are shown.

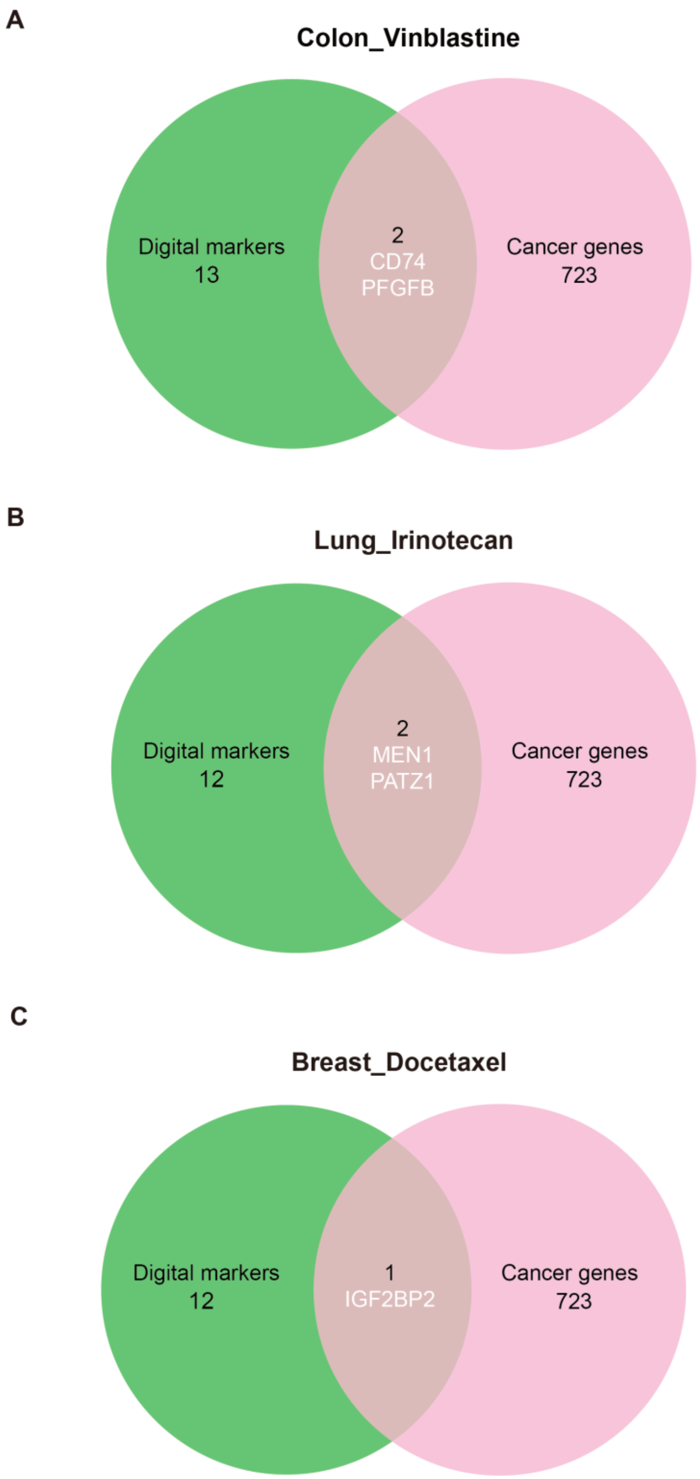

Figure S12. The Venn diagrams showing overlapping of the digital markers of cell lines and cancer genes. The overlapping of digital markers of several cancer type-drug pairs, including colon cancer and Vinblastine(A), lung cancer and Irinotecan(B), and breast cancer and Irinotecan(C) and cancer genes are shown.

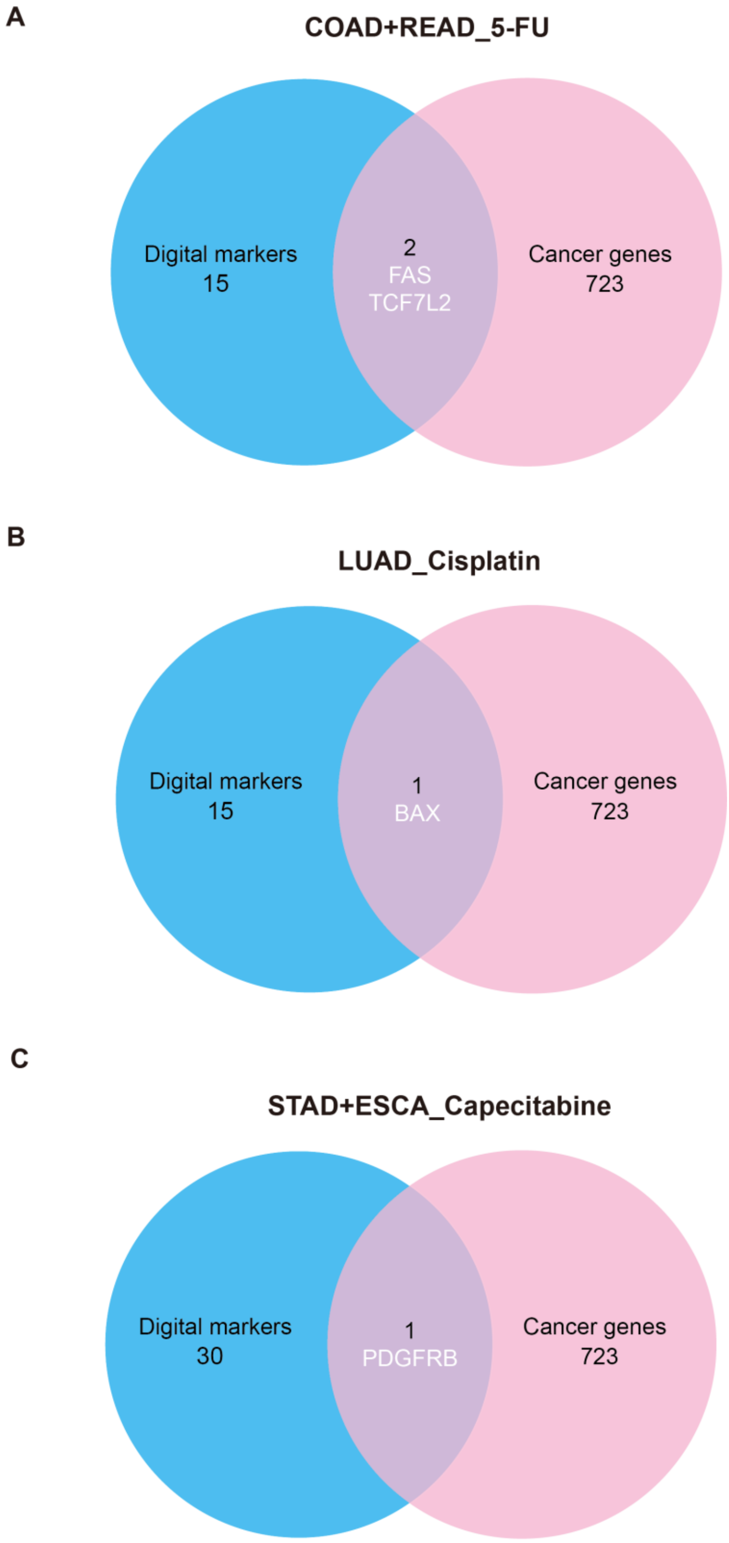

Figure S13. The Venn diagrams showing overlapping of the digital markers of patient samples and cancer genes. The overlapping of digital markers of several cancer type-drug pairs, including COAD, READ and 5-FU(A), LUAD and Ciaplatin (B), and STAD, ESCA and Capecitabine(C) and cancer genes are shown.

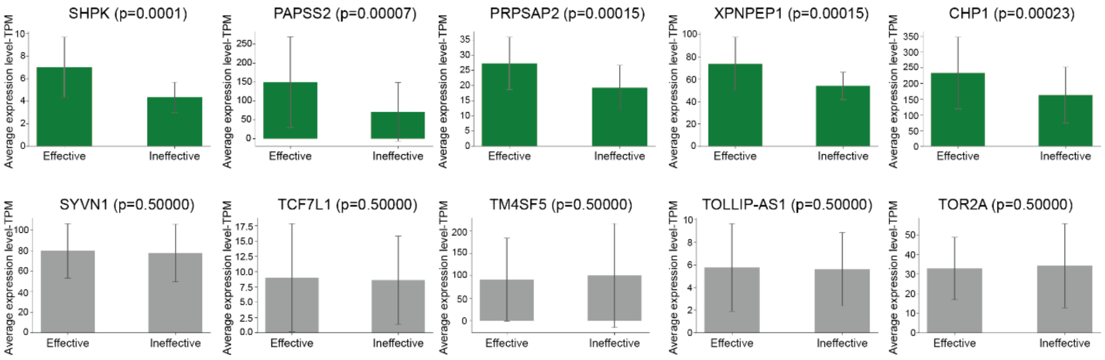

Figure S14. The retained and filtered-out genes of the Mann-Whitney U test. The illustration of Mann-Whitney U test filter. The transcription levels of the genes across all drug-sensitive and non-sensitive samples are tested by Mann-Whitney U test. Genes with p-values less than 0.1 are retained and rated (by the p-values). The top 5 figures characterize the retained genes, and the bottom 5 figures characterize the filtered-out genes. (Drug: 5-FU; cancer type: COAD and READ)

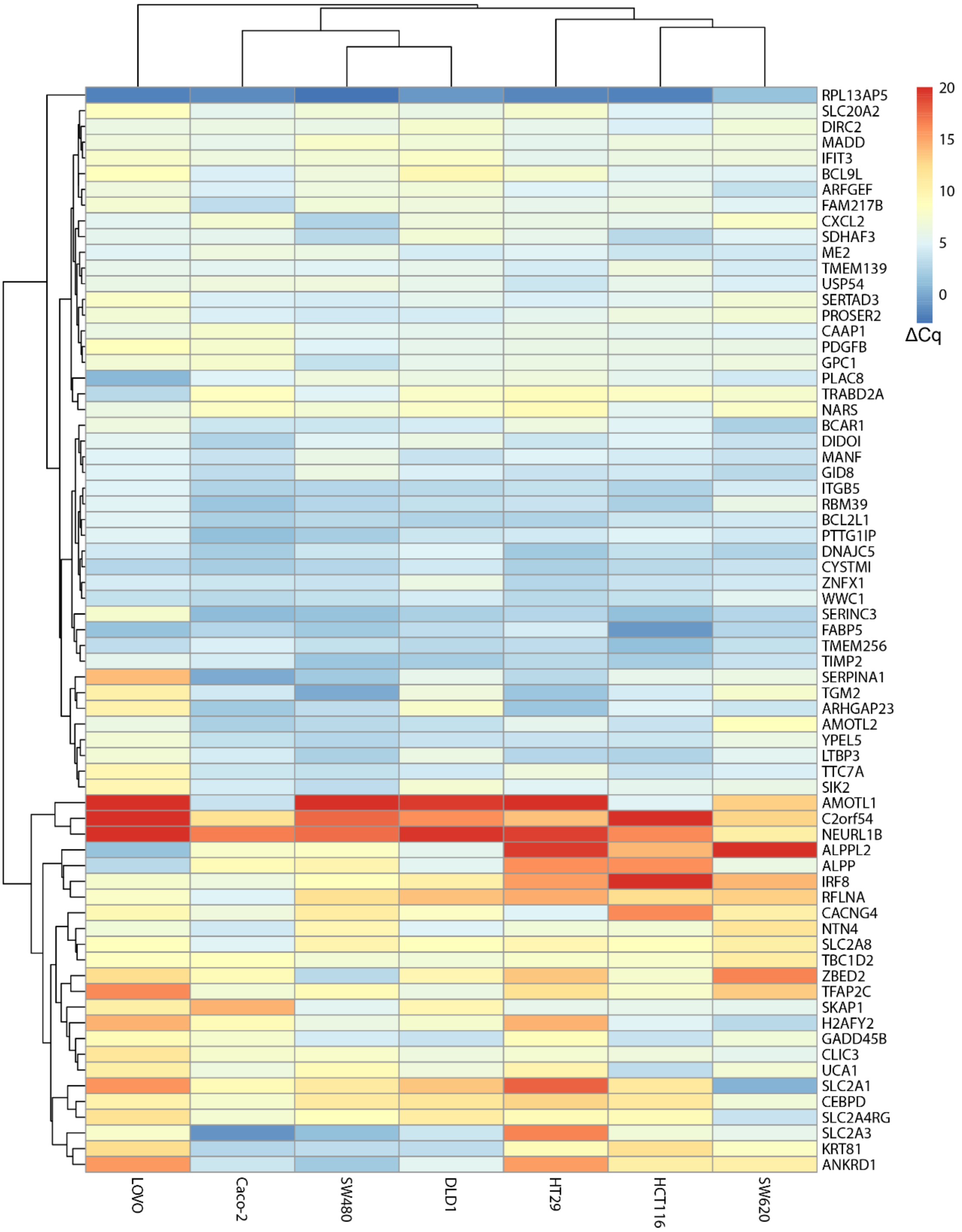

Figure S15. Gene transcription levels of the digital marker genes of cancer cell lines. The columns represent the samples (cell lines). The rows represent the digital markers (for Cisplatin, 5-FU and Paclitaxel and colorectal cancer cell line). The colors denote the transcription levels.

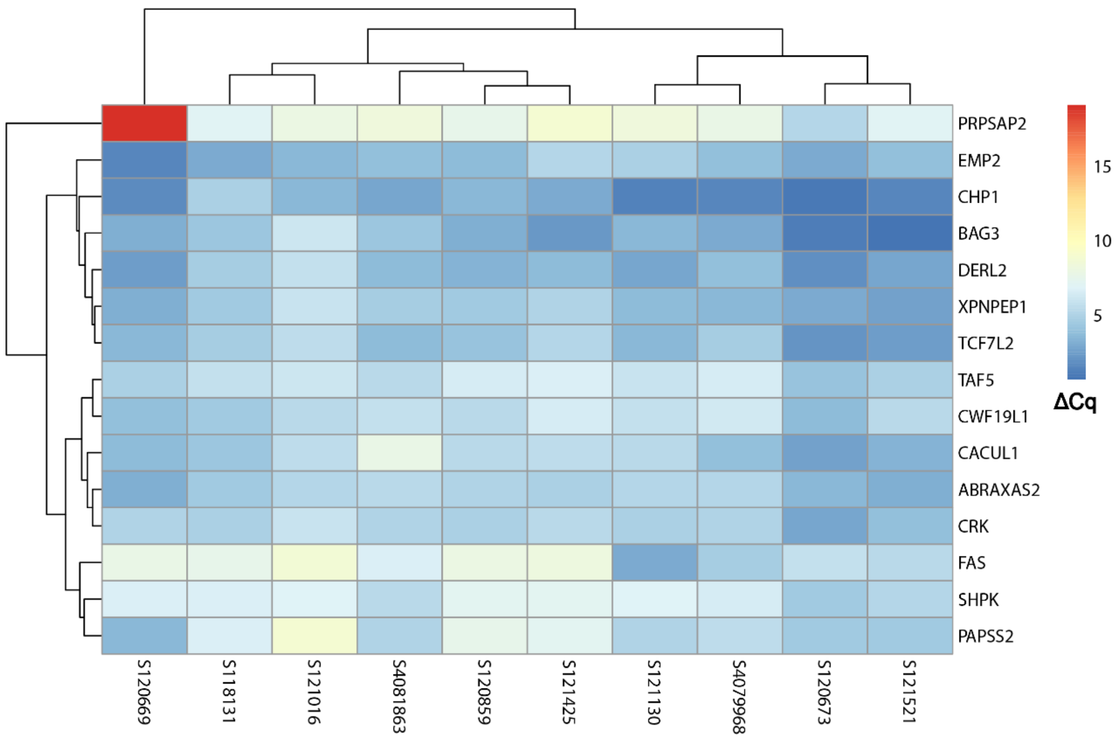

Figure S16. Gene transcription levels of the digital marker genes of patient derived tumors for effectiveness prediction of 5-FU. The columns represent the samples (tumor organoids). The rows represent the digital marker (for 5-FU and colorectal cancer tumor). The colors denote the transcription levels.

Table S1. Drug efficacy classification of prognostic data in TCGA.

In TCGA, prognostic information has four classes, CR, PR, SD and PD. In this study, we group CR, PR and SD as "effective" treatment, and PD as "ineffective" treatment.

**Considered as Effective/Ineffective**	**Treatment best response**	**Explanation**
Effective	Complete response(CR)	All target lesions disappeared without new lesions, and tumor markers were normal for at least 4 weeks.
	Partial response(PR)	The sum of the maximum diameter of the target lesion decreased by at least 30% for at least 4 weeks.
	Stable disease(SD)	The sum of the maximum diameter of the target lesion was not reduced to PR or increased to PD.
Ineffective	Progressive disease(PD)	The sum of the maximum diameter of the target lesion increased by at least 20%, or new lesions appear.

Table S2. The analyzed cancer type-drug pairs in this study.

Cell line/Patient sample	Cancer type	Drug	Dataset
Cell line	Colorectal cancer	5-FU, Cisplatin, Cyclophosphamide, Paclitaxel, Vinblastine, Docetaxel, Oxaliplatin, Irinotecan	CCLE & GDSC
	Lung cancer		
	Breast cancer		
Patient sample	Colon Adenocarcinoma (COAD), Rectum Adenocarcinoma (READ)	5-FU, Oxaliplatin	TCGA
	Lung Adenocarcinoma (LUAD)	Cisplatin	
	Stomach Adenocarcinoma (STAD)	5-FU, Capecitabine, Cisplatin	

Table S3. The digital markers for cell line drug sensitivity prediction. For most cases, the digital markers are screened by using both datasets, CCLE and GDSC. For breast cancer cell lines, the digital markers of 5-FU and Paclitaxel are screened by using CCLE only, due to a sharp decrease in performance of cross-validation using both datasets.

**Cancer type**	**Drug**	**Digital markers**
Colon	5-FU	ARHGDIB, TRAF5, CDC42EP3, RFLNA, CNNM4, SHC1, PAM16, CHID1, ZBED2, PAK4, CSRNP1, L1CAM, SFXN4, ZNF200, NPIPB15, C6orf203, SLC35F5, SLC18B1, SERTAD3, UPK2, CACNG4 (21 genes)
	Cisplatin	PLBD1-AS1, NOP9, MANF, IRF8, PIWIL4, GID8, SLC20A2, TSPAN3, USP54, TMEM164, KIAA0232, ATR, SLC30A1, SENP2, LINC01133, TAF4, TMEM139, CLIC3, YTHDF1, U2SURP, TMEM256, PTTG1IP, SLC2A1, DNAJC5, WWC1, KRT81,HEPH, PNKD (28 genes)
	Paclitaxel	MPP1, ALPPL2, FAM81A, PLAC8, RFLNA, POFUT1, DYNLRB1, SLC2A3, FOXP1, SBK1 (10 genes)
	Cyclophosphamide	CLDN2, SYT13, SLC6A9, ATP5MPL, CEBPD, DYNLRB1, SLC2A4RG, CNN2, ABHD12, SERPINA1, TRABD2A, RALY, BCL9L, STAU1 (14 genes)
	Vinblastine	FOLR1, PLXND1, EIF4BP6, CD74, PDGFB, PDGFA, SLC2A3, LINC00909, INTS6L, PFAS, MPP1, CTDNEP1, EGR1 (13 genes)
	Docetaxel	MPP1, SFXN5, POFUT1, GID8, DYNLRB1, SLC30A1, PGM2, SLC2A3, OSBPL2, NTN4, KLHDC2 (11 genes)
	Oxaliplatin	CEBPA-DT, CEBPD, GAD1, FBXL18, RC3H2, PDZD11, DSN1, IFIT3, CIZ1, PLBD1-AS1, FKBP4, MARCKS, DNAJC5, ABCD1, CTNNBL1, TLE1, PRKD2, CSTF1, EIF4G1, ADNP, ZNF106, FBXL8, PIK3R4, HPS5, ACTR1A, WNK4 (26 genes)
	Irinotecan	RPL34, PALD1, UBE2V1, CXorf56, TSC22D2, EEF1A2, POLR1C, ZFP64,

		HCG11, MPP1, HIKESHI, PXDC1, KLHDC2, TFAP2C, A4GALT, SPATA2 (16 genes)
Lung	5-FU	FAM3A, PDZD11, PLEKHM1, NEU1, TNFAIP2, SMPD1, MEA1, SNX2, BRPF3, NAGK, C17orf51, ACTR1A, AGK, RCN3, SPAG9, FAM50A, DENND4A, MMGT1, CYC1, PDE6D, DHX40, TMEM178B (22 genes)
	Cisplatin	IL18, MIR4435-2HG, ORAI3, NT5E, CPNE8, SQOR, CYTOR, GNG4, LIF, CPSF6 (10 genes)
	Paclitaxel	CTSZ, IL18, C19orf33, LIF, GPRC5A, FAM214B, NT5E, OSMR, SP100, MET, KRT80, GBP3, KRT18, CAV2, NCEH1, VSIR, PPARG, AHNAK, LAPTM4A, TTLL12, ALDH3B1 (21 genes)
	Cyclophosphamide	C19orf33, IL18, GPRC5A, NT5E, SYT11, CAV2, KRT80, OSMR, DFFB, VSIR, ADIRF-AS1, MYH9, AHNAK, UBE2H, MYOF, ALDH3B1, CAST, CAVIN1, ATP2A1-AS1, CTSZ, CCDC28B, MARK1, BEX1 (23 genes)
	Vinblastine	OIP5, CBX2, CAPN2, VSIR, GCDH, COQ3, NABP1, HERC2, TEDC2, BUB1B, GPRC5A (11 genes)
	Docetaxel	LIF, RUNX1, SP100, C19orf33, CAVIN3, TMCO4, GBP3, CBX2, CAV2, TTLL12, NT5E (11 genes)
	Oxaliplatin	GADD45B, RRAS, HERC2, LGALS1, MARK1, AHNAK, CAV2, SLC10A3, S100A10, 1-Mar, FAN1, RMND5A, FLII, FAM117B, MIR22HG, PXN, DRAP1, CALU, MYADM, TMEM200B (20 genes)
	Irinotecan	HKDC1, MEN1, PATZ1, PLA2G16, PDGFC, HEXB, SRSF7, PSAP, SCAF4, HNRNPUL2, MTA2, ABCC3 (12 genes)
Breast	5-FU	INTS7, CENPF, SIRPA, ZNF229, SLC25A12, SS18L2, RFX5, RAB18, GSN, JAG1, CHST14 (11 genes)
	Cisplatin	TFF3, KRT5, TFF1, CACNG4, CBR1, JAG1, TSPYL5, GREB1, MPP7, PRRT3, SOAT1, COL12A1, ACSL5, TMEM51, STC2, ABAT, ESR1, PPM1K, HOXC8 (19 genes)

	Paclitaxel	TFF1, CRYBG3, ESR1, TFF3, ABAT, GHR, RGL1, TSC1, FTLP14, STAC, TMEM116, ING1, HIST1H2BF, RACK1 (14 genes)
	Cyclophosphamide	MGST1, C19orf33, ZSCAN31, GNB4, PTK7, ZNF185, MOV10, CACNA1H, RIN2, EVC, MIR4458HG, EEF1A2, NHLRC1, HIST1H2BG, TLE1, GALC, APBB1, SLC7A2 (18 genes)
	Vinblastine	AREG, ELMO3, CDH1, ABCA12, PARD6A, POF1B, FGD1, CRABP2, EPPK1, ZNF513, TMEM191A, SLC29A4, HR, MGP, GAL, DGKE, ENPP5, SHISA2, EGLN2, RNU1-106P (20 genes)
	Docetaxel	IGFBP5, TFF3, FXYD5, FSCN1, CA12, TFF1, IGF2BP2, TRIM29, CACNG4, SYCP2, AGR3, PRR15L(12 genes)
	Oxaliplatin	TRPS1, TFF1, CACNG4, SYCP2, PRLR, GREB1, MPP7, ATF3, GAL, APOE, ABAT, ENG, PPM1K, ESR1, HOXC8, ZNF512, OPTN, PREX1, NECTIN1, LMCD1, ST6GALNAC4 (21 genes)
	Irinotecan	TFF1, CACNG4, PRLR, PPARG, JAG1, PROM1, SH3BP1, TRIL, AR, CENPV, HSPB8, NEBL, C3orf14 (13 genes)

Table S4. The digital markers for patient drug effectiveness prediction. The genes colored red are digital markers selected by different drugs for a certain cancer type. The genes colored blue are digital markers that are also cancer genes.

Cancer type	Drug	Digital markers
COAD, READ	5-FU	SHPK, PAPSS2, PRPSAP2, XPNPEP1, CHP1, TAF5, ABRAXAS2, DERL2, EMP2, CACUL1, CWF19L1, FAS, BAG3, TCF7L2, CRK, MYEOV, CTDNEP1, MICU1, SCO1, DUT, SEMA4G, MCU, ALKBH5, EXOC6, GLOD4, GLUD1 **(26 genes)**
COAD, READ	Oxaliplatin	SHPK, MYEOV, DGKA, GPRC5A, KCTD5, LINC01273, MCU, SH3PXD2A-AS1, RASL10B, EMP2, LPAR5, XXYLT1, SLCO1B3, BAG3, IER3-AS1, RHOQP1, CHM, DPY19L2P2, FUNDC2P1, CLCF1, ZXDA, NBAT1, AZGP1P1, BCAS4, XRRA1, CASP7, LINC02012, ALKBH5 **(28 genes)**
LUAD	Cisplatin	MAMSTR, DBP, C2orf70, MGARP, THADA, BAX, CNPY3, FOXO6, ISYNA1, SSBP4, CNN3, RPIA, CYP2F2P, KCND1, TEAD1 , ZMAT2, PCP4 **(17 genes)**
STAD, ESCA	5-FU	HIST3H2A, FAHD1, VCX2, SEPHS2, FIBP, ZSWIM1, PRKACB, PRR7, PQBP1, ASPHD1, PAM16, GNPTG, SCAMP3, RPP25, LINC00605, VCX3A, SEZ6L2, TSPAN5, NAXE, PFDN2, CFL1, LINC01979, KLF2P4, NMRAL1, MPV17L, RPUSD1, TUFM **(27 genes)**
STAD, ESCA	Capecitabine	GNRHR2, GRIN2D, PGM2L1, PAIP2B, HOTTIP, BCAP31P1, CETP, ABAT, DAB2, GJA4, GJA5 **(11 genes)**
STAD, ESCA	Cisplatin	IDUA, SNHG5, GSPT1, ACHE, MBD2, UBE2L3, BBOX1, LINC02041, ATP5F1A, SHROOM1 **(10 genes)**

Table S5. Overlapped genes among the 1~200 ranked genes screened independently by CCLE and GDSC. Numbers in parentheses index the genes in the lists screened by CCLE and GDSC, respectively. The genes colored red are digital markers overlapped.

Cancer type	Drug	Overlapped genes
Colon	5-FU	ARHGDIB(1,80), RFLNA(97,1), ZBED2(107,132), PAM16(109,39), CSRNP1(126,167), ZNF200(197,116)
	Cisplatin	MANF(61,102), GID8(90,65), PIWIL4(97,51), SLC20A2(101,185), USP54(122,140), TMEM164(124,165), TSPAN3(126,87), ATR(178,72)
	Paclitaxel	DYNLRB1(66,43), FOXP1(75,99), RAB11B(96,151), CEBPD(97,37), ALPP(111,16), EIF3I(124,103), FGD4(132,46), PODXL(173,68)
	Cyclophosphamide	CLDN2(1,41), SYT13(24,160), SLC2A4RG(115,70), BCL9L(196,39)
	Vinblastine	FOLR1(11,157), CD74(30,16), SLC2A3(60,128), LINC00909(93,13), RPL6P27(180,189), KRT81(185,188)
	Docetaxel	MPP1(12,49), POFUT1(50,45), GID8(62,190), SLC30A1(64,192), DYNLRB1(66,119), OSBPL2(78,115), KLHDC2(82,98), PGM2(90,16), MED22(136,146)
	Oxaliplatin	CEBPD(34,12), DSN1(118,180), FBXL18(125,5), IFIT3(139,94)
	Irinotecan	PALD1(60,193), UBE2V1(63,157), EEF1A2(118,5), MPP1(165,133)
Lung	5-FU	FAM3A(58,24), PDZD11(89,44), TNFAIP2(103,154)
	Cisplatin	KIF5C(2,91), ORAI3(9,4), SQOR(19,22), MARCKSL1(25,65), CAV2(28,66), CAV1(31,94), CTSA(34,43), MYOF(41,61), CPSF6(47,30), H3F3A(53,80), OSMR(56,38), SP100(68,166), RRAS(96,131), FOSL1(97,143), CAVIN1(104,37), GNG4(106,18),

		CSTF3(115,55), CAST(117,123), ADAMTSL5(121,108), RAB3A(123,104), NT5E(124,2), VMP1(130,127), AXL(143,100), SP140L(145,29), LGALS1(150,158), MARK1(169,48), RPS6KL1(172,60), CYTOR (181,14), CBX2(184,76), LINC00857(198,172)
	Paclitaxel	TTLL12(13,152), CBX2(29,188), GBP3(38,47), DNAJC12(41,185), CAPN2(59,190), SGMS2(181,173), IL18(199,2)
	Cyclophosphamide	CAV2(4,33), C19orf33(6,2), AHNAK(12,70), ATP2A1-AS1(16,92), MYOF(22,79), CAVIN1(32,81), AHNAK2(39,178), SGMS2(45,116), CCDC28B(46,106), CTSZ(53,97), ALDH3B1(67,75), SYT11(72,13), ADIRF-AS1 (84,59), ZNFX1(86,141), VSIR(91,52), NCEH1(94,136), NT5E(125,7), MEX3A (136,126), BEX1(140,94), CAV1(156,135), SP100(159,118), CAPN2(169,123), OSMR(175,35), MAST1(184,183), IL18(185,3), ITGA3(198,95)
	Vinblastine	CBX2(9,135), CAPN2(22,125), GCDH(40,137), BUB1B(41,184), GPRC5A(102,160), PHLDA3(152,147), NABP1(165,84), ADSL(167,170), VSIR(173,72)
	Docetaxel	CBX2(1,47), CYR61(12,192), CAPN2(30,140), TTLL12(75,63), MIR17HG(76,110), CTSZ(86,86), CTSA(109,122), SAMM50(111,170), IL6ST(113,182), MIR221(127,82), CAV2(180,56)
	Oxaliplatin	GADD45B(45,22), MARK1(48,120), LGALS1(50 ,115), CALU(124,166), PXN(139,137), DRAP1(147,136), 1-Mar(167,78), MAML3 (173,145), MYADM(183,126)
	Irinotecan	PLA2G16(95,138), MEN1(193,82)
Breast	5-FU	ESR1(12,170), INPP4B(41,100), MPP7(45,79), TFF1(48,10), TMPRSS3(70,185), RANGRF(96,166),

		TFF3(146,3)
	Cisplatin	KRT5(19,4), TFF1(23,25), ACSL5(69,198), PRRT3(125,151), GREB1(167,127), CBR1(178,69)
	Paclitaxel	TFF1(0,18), TFF3(3,3), FXYD5(17,74), TMC4(42,71), SEC63(61,135), AGR3(67,46), ABCC11(85,98), GREB1(100,99), UST(131,157), MINDY1(140,162)
	Cyclophosphamide	C19orf33(176,47)
	Vinblastine	TMEM191A(24,124), POF1B(41,68), ABCA12(44,42), SLC29A4(47,164), HR(58,185), ELMO3(76,12), EPPK1(183,103), CDH1(197,28)
	Docetaxel	TFF1(0,26), TFF3(5,3), FXYD5(9,8), ABCC11(26,121), SOAT1(56,184), CA12(58,25), AR(66,168), DEGS2(84,169), SPATS2L(97,185), SYCP2(99,53), FKBP10(136,89), IGF2BP2(149,31), LYN(158,181), ALDH3B2(165,66), CACNG4(180,52)
	Oxaliplatin	ATF3(64,155), MPP7(65,153)
	Irinotecan	PROM1(4,136), PPARG(65,111), CACNG4(142,51), SH3BP1(144,150), PRLR(153,68)

Table S6. Top 20 important genes for drug sensitivity prediction on Colorectal, Lung and Breast cancer cell lines screened by gene filters across all clinical drugs available in GDSC and CCLE.

We screened and rated the genes important for drug sensitivity prediction on Colorectal, Lung and Breast cancer cell lines toward all clinical drugs. We found some genes with high predictive importance but not contained in the oncogene list(Thomas R K et al. Nature genetics, 2007, 39(3): 347-351). The examples are TGM2, SLC2A1, NT5E, ACKR3 and TFF1.

**Colorectal cancer**	**Lung cancer**	**Breast cancer**
TGM2	NT5E	ACKR3
SLC2A1	RGS16	TFF1
UPK2	C19orf33	ESR1
CEBPD	DPYSL5	TFF3
CD74	KIF5C	GREB1
BCL2L1	KIF1A	ELF5
DYNLRB1	AXL	EMB
ARHGAP23	MIR221	DSCAM-AS1
SLC2A4RG	FZD3	CYP2T1P
TMEM139	EYA2	C19orf57
DNAJC5	NAV3	DEGS2
LBH	INSM1	SHISA2
PTTG1IP	SYP	PROM1
CLIC3	GBP3	CCDC113
BLCAP	SAMD4A	RGL1
ME2	OXTR	CACNA1H
SLC20A2	HRH1	ELOVL2
MANF	SNAP25	WASF3
ITGB5	RAB3A	ABAT
TM9SF4	SLFN12	SLC7A2

Table S7. Cancer genes among the 1~200 ranked genes screened by the gene filters (using both datasets CCLE and GDSC). Numbers in parentheses index the genes in the lists. The genes colored red are cancer genes among digital markers.

Cancer type	Drug	Cancer genes
Colon	5-FU	LATS2(34), SH3GL1(41), POT1(54), MLLT3(81), HNF1A(105), LEPROTL1(148), MECOM(175), PDGFB(176), PRKAR1A(181), TSC2(196)
	Cisplatin	ATR(11), GATA3(56), RPL5(92), NCOA1(98), CREB3L1(112), FOXP1(116), CHD4(135)
	Paclitaxel	FOXP1(8), SDC4(37), FGFR1(68), GATA3(83), MECOM(94), CREB3L1(95), MGMT(134), MNX1(173)
	Cyclophosphamide	BCL9L(12), TMEM127(35), ASPSCR1(104), POU5F1(121), FOXP1(124), RPL5(153), CHD4(166), TNC(169), CDKN2A(194)
	Vinblastine	CD74(3), PDGFB(4), SDC4(28), FGFR1(83), CHEK2(87), FOXP1(95), VTI1A(135)
	Docetaxel	SDC4(27), FGFR1(59), GATA3(75), MECOM(84), CREB3L1(85), FOXP1(114), MGMT(124), MNX1(166)
	Oxaliplatin	KAT6A(28), RBM15(32), MECOM(74), FBXO11(111), MNX1(118), NCOA1(139)
	Irinotecan	CXCR4(85), MNX1(116), NUP214(190), EWSR1(196)
Lung	5-FU	BRAF(32), MYO5A(49), NCKIPSD(52), CRNKL1(56), PTPN11(83), FLI1(120), CYLD(129)
	Cisplatin	H3F3A(25), CAMTA1(35), CXCR4(56), EGFR(92), EZH2(95), BCOR(102), BCL9L(121), ACVR1(139), MYH9(150), CNTNAP2(153), TFE3(162), IL6ST(170), TMEM127(179)
	Paclitaxel	MET(9), PPARG(16), EPAS1(31), LASP1(33), IL6ST(44), SDC4(77), EGFR(92), WNK2(93), PDGFB (105), RUNX1(112), CARD11(139), TFE3(193)
	Cyclophosphamide	MYH9(11), CAMTA1(47), EGFR(48), IDH2(62), MYB(66), EPAS1(72), IL6ST(88),

		PATZ1(101), CARD11(135), TBX3(144), SBDS(161), PPARG(167), MET(178), TFE3(183), NT5C2(196)
	Vinblastine	BUB1B(9), NTHL1(18), WDCP(32), LASP1(37), MAML2(51), TFE3(57), CAMTA1(58), PATZ1(62), TSC1(104), IL6ST(106), SBDS(118), KNL1(119), ERCC3(132), AFF4(137), PMS2(155), DDB2(158), CLIP1(183)
	Docetaxel	RUNX1(1), IL6ST(28), EGFR(37), LASP1(46), WNK2(63), MET(79), PPARG (83), CASP8(87), CBFA2T3(93), CARD11(105), TFE3(115), TNFAIP3(127), PATZ1(156), ACVR1(166), NT5C2(167), POLE(168), PDGFB (169), CAMTA1(196)
	Oxaliplatin	LMNA(40), LASP1(41), PATZ1(49), RUNX1(100), TFE3(117), WNK2(132), ACVR1(140), SBDS(151), MNX1(152), CARD11(164), IL7R(168)
	Irinotecan	MEN1(1), PATZ1(2), PBRM1(37), NUMA1(68), TGFBR2(74), LATS2(76), VHL(86), PIK3CB(92), CHD4(94), NCOA2(103), GPHN(114)
Breast	5-FU	ESR1(13), CBLC(119), MLLT6(160), FOXO3(173), PTPRK(176)
	Cisplatin	ESR1(16), SIRPA(25), CD74(62), IGF2BP2(63), MET(66), EGFR(71), MSN(80), FGFR3(102), PIM1(108), CREB3L1(117), PPARG(138), CBLC(152), ERBB2(158), AR(196)
	Paclitaxel	ESR1(23), RET(33), CXCR4(60), CREB3L1(128), ERBB2(163), GATA2(175), CBLC(176)
	Cyclophosphamide	CHCHD7(28), FGFR2(33), FAT1(99), SYK(101), ETV4(119), STAG2(126), SIRPA(152), ALDH2(168), FBLN2(181), MYB(190), ETV5(199)
	Vinblastine	CDH1(2), FGFR3(89), ACKR3(169)
	Docetaxel	IGF2BP2(6), PIM1(13), TGFBR2(20), AR(26), ACKR3(37), ESR1(43), MAML2(49), SIRPA(56), MET(110), EGFR(115), MSN(124), FGFR3(142), CREB3L1(156), CBLC(184), ERBB2(192)
	Oxaliplatin	ESR1(13), CD74(55), IGF2BP2(56),

		MET(59), EGFR(64), MSN(73), FGFR3(96), PIM1(102), CREB3L1(112), PPARG(137), CBLC(154), TGFBR2(159), ERBB2(162)
	Irinotecan	PPARG(3), AR(8), CHST11(16), ESR1(19), MAML2(27), SIRPA(31), PPM1D (33), CD74(77), IGF2BP2(79), MET(82), EGFR(86), FGFR3(116), PIM1(122), CREB3L1(132), CBLC(168)
